# Global burden trends of tension-type headache, 1990–2021: socio-demographic patterns, age-period-cohort effects, and frontier analysis from the GBD 2021 study

**DOI:** 10.3389/fneur.2025.1629025

**Published:** 2025-07-16

**Authors:** Can Wang, Chao Liao, Yuyin Liu, Peng Chen, Yuanlun Xie, Li Tian

**Affiliations:** ^1^Hospital of Chengdu University of Traditional Chinese Medicine, Chengdu, China; ^2^Chengdu University of Traditional Chinese Medicine, Chengdu, China; ^3^Affiliated Hospital of Panzhihua University, Panzhihua, China; ^4^School of Electronic Information and Electrical Engineering, Chengdu University, Chengdu, China

**Keywords:** tension-type headache, Global Burden of Disease study, Socio-Demographic Index, age-period-cohort analysis, frontier analysis

## Abstract

**Background:**

Tension-type headache (TTH) is one of the most prevalent neurological disorders globally. While previous studies have examined TTH epidemiology across different regions, few have comprehensively analyzed how socio-demographic factors influence its patterns and trends. This study aimed to analyze TTH burden trends from 1990 to 2021 and their relationship with socio-demographic development.

**Methods:**

Data on prevalence, incidence, and years lived with disability (YLDs) for TTH were extracted from the Global Burden of Disease study (GBD) 2021, covering global, 5 SDI regions, 21 regions, and 204 countries. We used absolute numbers and age-standardized rates from 1990 to 2021. Spearman correlation assessed the relationship between rates and the Socio-Demographic Index (SDI). An age-period-cohort model disentangled temporal effects across different SDI levels, while frontier analysis evaluated improvement potential relative to SDI.

**Results:**

Despite modest decreases in age-standardized rates (prevalence: −0.56%, incidence: −0.32%, YLDs: −2.27%), absolute TTH cases reached 2.01 billion in 2021, increasing 56.4% from 1990. TTH burden showed moderate positive correlations with SDI, with High SDI regions having highest rates. Contrasting trends emerged: globally and in High and High-middle SDI regions rates decreased, while Middle SDI regions showed significant increases (prevalence: +5.83%). APC analysis revealed global peak prevalence at age 32.5 years, with period effects showing decreases in High SDI regions but increases in Middle SDI regions. Cohort effects indicated decreasing risks in recent High/High-middle SDI cohorts but increasing risks in Middle SDI regions.

**Conclusion:**

Our research reveals that TTH remains a substantial global health challenge, with its burden increasingly shifting toward younger populations in middle-income countries. This epidemiological transition, coupled with significant untapped potential for burden reduction even in high-SDI nations, demands the urgent development of context-specific public health strategies to mitigate the growing impact of this highly prevalent disorder.

## Introduction

Headache disorders represent a major global public health concern, ranking among the leading causes of disability worldwide and imposing substantial socioeconomic costs due to reduced productivity and impaired quality of life (1, 2). Among primary headache disorders, tension-type headache (TTH) is the most common, affecting a significant proportion of the global population across all ages (3, 4). While often perceived as benign, its high prevalence and association with risk factors common in modern society—including psychological stress, sleep disturbances, and potential genetic predispositions—make it a significant public health issue (5). Characterized typically by mild to moderate bilateral headache, TTH, particularly in its chronic form, can lead to considerable personal suffering and functional impairment (6). Understanding the complex interplay between TTH burden and societal development is crucial for tailoring effective public health strategies globally.

Previous epidemiological studies, including prior GBD analyses, have established TTH as a ubiquitous condition but also highlighted significant variations in its burden across different settings (7). While factors like age and sex are known contributors—with higher rates often observed in females and middle-aged adults (3, 8). Growing evidence suggests that the burden of headache, including TTH, may increase with rising socioeconomic status, possibly linked to lifestyle changes, increased stress, or improved diagnostic ascertainment (9). However, the drivers behind these observed trends remain insufficiently characterized. A key conceptual gap is that conventional trend analyses cannot disentangle the independent contributions of population aging (age effects), period-specific environmental or healthcare factors (period effects), and generational shifts in risk (cohort effects). This makes it difficult to determine whether observed changes in TTH burden stem from population aging, contemporary environmental shifts, or genuine generational differences in disease risk.

Therefore, this study leverages data from GBD 2021 to provide a comprehensive assessment of the global TTH burden from 1990 to 2021, with a particular focus on its relationship with socio-demographic development. To address the methodological challenge of disentangling temporal influences, we employ age-period-cohort (APC) analysis stratified by SDI level, allowing us to explore how age, period, and cohort effects differ across varying levels of socioeconomic development (10, 11). Additionally, to identify exemplary societal models, we apply frontier analysis to benchmark countries against the best performers at their respective SDI levels, thereby revealing achievable targets and quantifying improvement potential (12, 13).

Specifically, our objectives were to: (1) assess global, regional, and national trends in TTH prevalence, incidence, and YLDs from 1990 to 2021; (2) explore the relationship between TTH burden and socio-demographic development; (3) disentangle the effects of age, period, and birth cohort on TTH trends across different SDI regions; and (4) quantify the potential improvement space for TTH burden relative to SDI.

## Methods

### Study population and data collection

We analyzed data from the Global Burden of Disease (GBD) 2021 database to assess the global disease burden of TTH from 1990 to 2021. The GBD study systematically collects and synthesizes epidemiological data from multiple sources, including published literature, population-based surveys, and administrative records. To generate consistent estimates for each disease across all locations, the GBD project utilizes a Bayesian meta-regression tool, DisMod-MR 2.1. This modeling process allows for the integration of disparate data points and addresses data scarcity, resulting in the model-based estimates used for this analysis (1). The GBD 2021 database includes comprehensive estimates of disease burden across 204 countries and territories for 371 diseases and injuries (14, 15). For this analysis, we extracted data on TTH, which was defined based on the reference case definitions satisfying the International Classification of Headache Disorders-3 (ICHD-3) criteria, as specified by the GBD framework.

We extracted the following TTH burden metrics from the GBD 2021 database: prevalence, incidence, and years lived with disability (YLDs). For each metric, we obtained both absolute numbers and age-standardized rates (expressed per 100,000 population). We extracted data at three geographic levels: global, 5 SDI regions, 21 GBD regions, and 204 countries. All data were further stratified by age group and sex.

### Socio-Demographic Index

GBD 2021 categorizes countries according to the Socio-Demographic Index (SDI), a composite measure that captures sociocultural and macroeconomic factors influencing health outcomes across locations. The SDI incorporates three key measures: absolute fertility rate (<25 years), mean education (≥15 years), and income per capita, representing their geometric average.

### Statistical analysis

First, we examined the numbers and age-standardized rates (ASRs) of TTH prevalence, incidence, and YLDs at global, regional, and national levels for 1990 and 2021, along with the percentage changes between these time points.

Second, we examined the relationship between TTH burden and socio-demographic development using Spearman’s rank correlation coefficient (rho) between ASRs and SDI values across countries. Spearman’s correlation was chosen over Pearson’s coefficient due to the non-normal distribution of TTH burden indicators and because we aimed to detect monotonic relationships rather than strictly linear associations. To further explore and visualize the non-linear relationship patterns between TTH burden and SDI, we applied locally weighted regression (LOESS) with a smoothing span of 0.5. Sensitivity analyses were performed using different smoothing parameters (0.3 and 0.7) to ensure robustness of the fitted curves.

Third, we conducted age-period-cohort (APC) analysis to decompose temporal trends into age effects, period effects, and cohort effects. The APC identifiability problem was addressed using the intrinsic estimator method, which provides a unique solution by constraining the sum of period and cohort effects to zero (11). Reference categories were set as the median period (2002–2006) and median cohort (born in 1957) to facilitate interpretation. Age groups were categorized into 5-year intervals from 5–9 to 95 + years (excluding <5 years), time periods into 5-year intervals from 1992–1996 to 2017–2021, and birth cohorts were derived accordingly from these age and period combinations. By performing this analysis at both the global level and across the 5 SDI regions, we elucidated the differential patterns and magnitudes of these temporal effects across varying socio-demographic development levels.

Finally, we performed frontier analysis to identify the theoretical minimum burden of TTH achievable at each SDI level. “Improvement potential” is defined as the difference between a country’s observed burden and the frontier value (minimum achievable burden) at its corresponding SDI level. To ensure robust frontier estimation, we employed a bootstrap approach with 100 iterations, using sampling with replacement. In each iteration, we identified super-efficient observations and excluded them to avoid outlier influence. The frontier was then constructed by identifying the minimum burden value at each SDI level among the remaining observations. The final frontier curve was derived using LOESS regression with a span parameter of 0.2, which provided optimal balance between capturing local variations and maintaining smoothness across the SDI spectrum. This approach represents the best-performing countries at each development level while accounting for sampling variability (13).

All analyses were performed using R version 4.4.2. Statistical significance was defined as *p* < 0.001 to control for multiple comparisons and reduce the risk of false positive findings given the large number of countries and time periods analyzed. Following GBD convention, we report uncertainty intervals (UIs) which capture model uncertainty, while confidence intervals (CIs) are used specifically for percentage change estimates were noted in table legends.

## Results

### Global burden and trends of TTH from 1990 to 2021

TTH represents a significant global health burden. In the context of all detailed causes assessed by GBD 2021, TTH ranked as the second most prevalent condition globally in 2021, even holding the top prevalence rank in high-SDI regions (Supplementary Figure S1A). While its global rank for age-standardized incidence was lower at eighth (Supplementary Figure S1B), the sheer scale of its prevalence is underscored by the substantial increase in absolute cases. From 1990 to 2021, the absolute burden of TTH increased substantially, with prevalent cases rising by more than half to reach over 2 billion individuals globally. Similar increases were observed for incidence cases and YLDs. Consistent with established patterns, TTH burden was markedly higher in females than males and peaked during middle age (Tables 1–3).

**Table 1:** Prevalence of TTH in 1990 and 2021.

Location	1990		2021		Percentage change in ASR from 1990 to 2021 (95% CI)
	Number (millions, 95% UI)	ASR (per 100,000, 95% UI)	Number (millions, 95% UI)	ASR (per 100,000, 95% UI)	
**Global**	1286.37 (1122.5, 1467.16)	24904.85 (21960.05, 28038.8)	2011.61 (1776.54, 2270.86)	24764.77 (21863.62, 27954.74)	−0.56 (−1.27, 0.18)
**Sex**					
Male	616.82 (537.23, 705.26)	23782.48 (20933.54, 26745.2)	969.38 (851.72, 1095.95)	23880.79 (21046.24, 26935.06)	0.41 (−0.54, 1.4)
Female	669.54 (586.17, 763.31)	25998.32 (22971.7, 29282.6)	1042.24 (923.03, 1176.69)	25634.41 (22631.48, 28974.28)	−1.4 (−2.37, -0.52)
**SDI**					
High SDI	292.36 (260.36, 328.16)	31223.39 (27664.95, 35156.3)	362.97 (323.19, 402.24)	30603.28 (27152.87, 34513.4)	−1.99 (−3.12, −0.85)
High-middle SDI	263.34 (231.79, 298.8)	24057.94 (21242.94, 27054.31)	336.59 (298.43, 376.78)	23862.61 (21031.88, 26904.35)	−0.81 (−2.42, 0.64)
Middle SDI	368.97 (319.01, 424.64)	22090.8 (19400.82, 24, 813)	599.79 (529.38, 676.82)	23378.72 (20608.16, 26365.06)	5.83 (4.63, 7.13)
Low-middle SDI	263.02 (225.6, 303.51)	25000.69 (21974.21, 28276.83)	481.76 (419.78, 552.03)	25022.79 (22033.7, 28284.15)	0.09 (−0.26, 0.45)
Low SDI	97.35 (83, 113.44)	23026.92 (20181.56, 26269.14)	228.82 (195.2, 266.94)	22780.22 (19950.88, 25943.94)	−1.07 (−1.42, -0.74)
**Region**					
High-income Asia Pacific	55.46 (49.12, 62.08)	29648.46 (26174.41, 33, 305)	60.74 (54.07, 67.51)	29867.61 (26437.5, 33548.63)	0.74 (−1.8, 3.54)
High-income North America	104.16 (93.23, 116.25)	35056.31 (31332.89, 39267.47)	135.18 (121.04, 149.99)	34180.26 (30603.85, 38251.42)	−2.5 (−5.11, 0.32)
Western Europe	135.2 (120.41, 152.43)	32654.11 (28897.95, 36899.33)	153.92 (136.29, 171.72)	32770.54 (28999.01, 37, 028)	0.36 (−0.56, 1.28)
Australasia	5.82 (5.08, 6.64)	27223.05 (23758.03, 31083.38)	8.99 (7.91, 10.15)	27215.52 (23764.26, 31068.68)	−0.03 (−0.36, 0.27)
Andean Latin America	7.33 (6.21, 8.6)	20434.79 (17625.22, 23, 491)	13.91 (11.8, 16.16)	20715.91 (17622.56, 23911.58)	1.38 (−1.65, 4.55)
Tropical Latin America	43.15 (37.57, 49.47)	28921.64 (25606.06, 32627.54)	68.93 (61.16, 77.28)	29005.61 (25648.55, 32612.92)	0.29 (−1.98, 2.45)
Central Latin America	37.4 (32.07, 43.76)	24426.6 (21383.91, 27946.68)	64.2 (56.25, 73.52)	24421.55 (21365.98, 27955.62)	−0.02 (−0.17, 0.12)
Southern Latin America	12.8 (11.07, 14.71)	26063.87 (22667.27, 30010.09)	18.86 (16.36, 21.59)	26264.27 (22771.94, 30205.1)	0.77 (−0.46, 1.94)
Caribbean	8.2 (6.92, 9.69)	23734.94 (20295.34, 27705.05)	11.66 (10.07, 13.54)	23730.24 (20, 293, 27687.86)	−0.02 (−0.22, 0.18)
Central Europe	39.87 (35.19, 45.18)	30462.47 (26744.42, 34714.05)	38.18 (33.81, 42.72)	30488.91 (26793.28, 34697.76)	0.09 (−0.22, 0.37)
Eastern Europe	73.73 (65.65, 82.41)	31140.41 (27760.76, 34998.16)	69.48 (62.21, 76.92)	31243.12 (27766.56, 34948.02)	0.33 (−1.55, 2.18)
Central Asia	19.74 (16.84, 22.94)	30290.56 (26245.04, 34563.38)	29.1 (25.14, 33.29)	30274.55 (26183.72, 34528.86)	−0.05 (−0.25, 0.15)
North Africa and Middle East	72.33 (61.44, 84.38)	23891.52 (20719.3, 27511.97)	150.06 (129.3, 172.13)	24090.29 (20941.08, 27533.32)	0.83 (−0.31, 2.21)
South Asia	254.97 (220.93, 293.15)	25390.94 (22398.62, 28584.43)	483.24 (423.7, 550.14)	25405.47 (22430.09, 28583.39)	0.06 (−0.12, 0.21)
Southeast Asia	110.72 (94.61, 127.7)	25237.84 (22037.46, 28546.86)	183.41 (159.92, 207.82)	25256.68 (22085.09, 28547.33)	0.07 (−0.18, 0.33)
East Asia	211.34 (182.99, 242.02)	17188.29 (15101.02, 19389.89)	293.5 (259.91, 331.48)	18489.51 (16320.68, 20929.5)	7.57 (3.84, 11.7)
Oceania	1.3 (1.09, 1.53)	22134.8 (19003.81, 25553.61)	2.88 (2.43, 3.39)	22142.5 (18985.45, 25544.46)	0.03 (−0.25, 0.33)
Western Sub-Saharan Africa	40.32 (34.58, 46.57)	25105.32 (22070.41, 28482.82)	105.71 (90.45, 122.43)	25108.38 (22066.05, 28468.24)	0.01 (−0.21, 0.24)
Eastern Sub-Saharan Africa	30.32 (25.59, 36.04)	19169.85 (16634.01, 22083.92)	72.01 (60.93, 85.23)	18801.27 (16305.57, 21690.09)	−1.92 (−2.74, -1.11)
Central Sub-Saharan Africa	10.66 (8.95, 12.65)	23234.13 (20059.74, 27026.03)	28.08 (23.62, 33.29)	23, 232 (20084.38, 27079.59)	−0.01 (−0.28, 0.3)
Southern Sub-Saharan Africa	11.56 (9.92, 13.36)	24201.85 (21272.63, 27431.83)	19.58 (17.11, 22.45)	24193.08 (21262.85, 27462.85)	−0.04 (−0.25, 0.17)

UI, uncertainty interval; CI, confidence interval; ASR, age-standardized rate; SDI, Socio-Demographic Index.

**Table 2:** Incidence of TTH in 1990 and 2021.

Location	1990		2021		Percentage change in ASR from 1990 to 2021 (95% CI)
	Number (millions, 95% UI)	ASR (per 100,000, 95% UI)	Number (millions, 95% UI)	ASR (per 100,000, 95% UI)	
**Global**	470.3 (408.47, 527.85)	8960.34 (7815.06, 10074.35)	719.04 (629.22, 804.95)	8931.31 (7788.21, 10020.83)	−0.32 (−0.93, 0.31)
**Sex**					
Male	227.36 (197.2, 255.92)	8614.88 (7510.19, 9699.73)	349.19 (304.27, 390.35)	8660.4 (7542.2, 9677.13)	0.53 (−0.34, 1.32)
Female	242.94 (211.83, 272.39)	9298.86 (8129.93, 10434.81)	369.86 (324.5, 413.11)	9199.48 (8038.49, 10339.58)	−1.07 (−1.87, -0.15)
**SDI**					
High SDI	100 (87.58, 112.45)	10923.85 (9499.79, 12272.04)	122.31 (107.37, 135.88)	10775.96 (9398.95, 12094.03)	−1.35 (−2.59, -0.23)
High-middle SDI	94.79 (82.34, 106.64)	8698.51 (7586.46, 9762.81)	117.66 (102.78, 131.49)	8626.73 (7552.45, 9693.02)	−0.83 (−2, 0.45)
Middle SDI	137.82 (119.04, 155.65)	8088.06 (7057.12, 9076.82)	215.01 (187.76, 240.67)	8510.45 (7422.02, 9527.46)	5.22 (4.17, 6.32)
Low-middle SDI	99.68 (86.03, 112.02)	9095.81 (7930.71, 10164.2)	176.24 (153.2, 197.65)	9080.29 (7909.39, 10152.84)	−0.17 (−0.47, 0.07)
Low SDI	37.52 (32.34, 42.57)	8406.13 (7279.49, 9434.99)	87.23 (75.2, 98.46)	8320.37 (7204.9, 9339.03)	−1.02 (−1.29, -0.75)
**Region**					
High-income Asia Pacific	19.46 (17.1, 21.76)	10640.01 (9339.36, 11861.06)	20.38 (17.86, 22.68)	10575.36 (9238.77, 11884.61)	−0.61 (−2.71, 1.79)
High-income North America	35.28 (30.72, 39.8)	12140.85 (10504.52, 13663.26)	45.78 (40.27, 50.99)	12015.58 (10479.54, 13411.67)	−1.03 (−3.6, 1.93)
Western Europe	45.22 (39.58, 51.07)	11254.24 (9721.06, 12722.4)	50.56 (44.45, 56.49)	11261.96 (9744.87, 12737.18)	0.07 (−0.72, 0.79)
Australasia	2.06 (1.8, 2.31)	9803.19 (8493.69, 10987.28)	3.13 (2.72, 3.49)	9800.1 (8492.14, 10984.67)	−0.03 (−0.38, 0.26)
Andean Latin America	2.81 (2.43, 3.19)	7583.03 (6644.96, 8480.47)	5.06 (4.36, 5.69)	7549.76 (6525.47, 8445.39)	−0.44 (−2.74, 1.76)
Tropical Latin America	15.53 (13.49, 17.5)	10112.03 (8946.24, 11300.85)	23.97 (21.08, 26.83)	10315.47 (9058.22, 11520.72)	2.01 (−0.08, 3.93)
Central Latin America	14.04 (12.07, 15.83)	8835.26 (7701.28, 9932.7)	23.02 (20.07, 25.9)	8831.59 (7697.35, 9924.72)	−0.04 (−0.17, 0.1)
Southern Latin America	4.67 (4.08, 5.25)	9456.95 (8269.49, 10633.1)	6.68 (5.84, 7.51)	9484.47 (8269.25, 10692.38)	0.29 (−0.67, 1.18)
Caribbean	2.99 (2.58, 3.38)	8528.66 (7404.19, 9631.87)	4.13 (3.59, 4.65)	8527.23 (7397.5, 9623.3)	−0.02 (−0.23, 0.18)
Central Europe	13.95 (12.11, 15.68)	10822.76 (9359.56, 12179.13)	12.98 (11.4, 14.47)	10842.09 (9385.16, 12204.69)	0.18 (−0.06, 0.43)
Eastern Europe	25.94 (22.68, 29.09)	11159.48 (9747.68, 12473.22)	23.93 (20.94, 26.64)	11191.39 (9761.98, 12541.1)	0.29 (−1.57, 2.28)
Central Asia	7.14 (6.12, 8.11)	10670.52 (9180.2, 12087.97)	10.23 (8.76, 11.63)	10666.08 (9176.18, 12079.49)	−0.04 (−0.25, 0.15)
North Africa and Middle East	27.27 (23.41, 31.06)	8618.35 (7509.82, 9640.78)	54.06 (46.99, 60.87)	8640.5 (7519.47, 9705.39)	0.26 (−0.56, 1.17)
South Asia	97.21 (83.94, 109.4)	9324.66 (8143.39, 10409.46)	177.58 (155.11, 200.07)	9331.32 (8145.93, 10413.58)	0.07 (−0.09, 0.22)
Southeast Asia	40.93 (35.23, 46.26)	9039.33 (7858.96, 10154.77)	65.09 (56.78, 73.58)	9053.62 (7870.48, 10169.91)	0.16 (−0.06, 0.36)
East Asia	79.65 (68.69, 90.84)	6470.23 (5608.05, 7296.25)	105.51 (91.97, 118.96)	6839.78 (5943.52, 7714.05)	5.71 (2.92, 8.9)
Oceania	0.49 (0.42, 0.56)	7994.58 (6926.42, 8981.36)	1.06 (0.92, 1.21)	7997.15 (6928.66, 8988.07)	0.03 (−0.2, 0.32)
Western Sub-Saharan Africa	15.26 (13.12, 17.27)	8991.35 (7832.75, 10121.19)	39.95 (34.25, 45.35)	8987.78 (7841.19, 10111.95)	−0.04 (−0.24, 0.16)
Eastern Sub-Saharan Africa	11.98 (10.26, 13.73)	7158.63 (6220.15, 8071.48)	28.16 (24.06, 32.16)	7047.11 (6125.37, 7948.03)	−1.56 (−2.23, -0.91)
Central Sub-Saharan Africa	4.05 (3.45, 4.63)	8, 348 (7238.08, 9397.06)	10.61 (9.04, 12.11)	8347.57 (7236.3, 9, 393)	−0.01 (−0.32, 0.27)
Southern Sub-Saharan Africa	4.36 (3.75, 4.92)	8777.24 (7621.01, 9890.11)	7.15 (6.22, 8.14)	8772.62 (7622.37, 9875.33)	−0.05 (−0.24, 0.13)

UI, uncertainty interval; CI, confidence interval; ASR, age-standardized rate; SDI, Socio-Demographic Index.

**Table 3:** YLDs of TTH in 1990 and 2021.

Location	1990		2021		Percentage change in ASR from 1990 to 2021 (95% CI)
	Number (millions, 95% UI)	ASR (per 100,000, 95% UI)	Number(millions, 95% UI)	ASR (per 100,000, 95% UI)	
**Global**	2.85 (0.82, 9.56)	56.99 (16.79, 186.13)	4.6 (1.35, 15.01)	55.69 (16.13, 185.07)	−2.27 (−4.87, 0.97)
**Sex**					
Male	1.25 (0.34, 4.48)	49.75 (13.78, 171.18)	2.03 (0.56, 7.05)	49.35 (13.44, 172.38)	−0.82 (−3.68, 2.3)
Female	1.6 (0.48, 5)	64.13 (19.76, 193.9)	2.57 (0.79, 7.67)	62 (18.8, 189.03)	−3.32 (−6.18, 0.24)
**SDI**					
High SDI	0.65 (0.18, 2.22)	67.81 (18.37, 233.69)	0.84 (0.25, 2.81)	66.79 (18.15, 235.79)	−1.5 (−6.7, 1.45)
High-middle SDI	0.69 (0.22, 2)	63.1 (19.99, 182.76)	0.91 (0.29, 2.66)	59.93 (18.33, 179.03)	−5.02 (−9.86, -0.79)
Middle SDI	0.79 (0.22, 2.77)	49.28 (14.55, 166.34)	1.36 (0.4, 4.46)	51.54 (14.95, 174.76)	4.6 (−2.39, 14.09)
Low-middle SDI	0.52 (0.14, 1.93)	52.48 (14.78, 183.54)	1 (0.27, 3.52)	52.99 (14.84, 182.64)	0.98 (−5.88, 7.42)
Low SDI	0.2 (0.06, 0.74)	51.69 (15.34, 178.87)	0.48 (0.13, 1.71)	51.76 (15.45, 171.97)	0.13 (−4.78, 4.84)
**Region**					
High-income Asia Pacific	0.12 (0.03, 0.41)	63.52 (17.37, 216.49)	0.14 (0.04, 0.44)	63.72 (17.35, 216.52)	0.31 (−4.4, 5.23)
High-income North America	0.22 (0.06, 0.79)	71.29 (18.11, 265.03)	0.29 (0.08, 1.01)	70.01 (17.89, 257.69)	−1.79 (−6.4, 2.32)
Western Europe	0.31 (0.09, 1.08)	71.66 (19.18, 260.25)	0.36 (0.1, 1.26)	71.93 (19.05, 261.97)	0.39 (−5.82, 3.9)
Australasia	0.01 (0, 0.04)	60.62 (17.34, 196.88)	0.02 (0.01, 0.07)	60.99 (17.51, 200.38)	0.61 (−7.39, 8.13)
Andean Latin America	0.02 (0, 0.06)	47.63 (14.67, 178.77)	0.03 (0.01, 0.12)	48.09 (14.42, 173.97)	0.97 (−8.14, 10.5)
Tropical Latin America	0.08 (0.02, 0.31)	55.68 (14.75, 203.91)	0.14 (0.04, 0.5)	55.97 (14.51, 205.79)	0.53 (−4.78, 4.94)
Central Latin America	0.08 (0.02, 0.29)	53.13 (15.52, 186.54)	0.14 (0.04, 0.48)	53.44 (15.36, 180.62)	0.58 (−4.03, 5.18)
Southern Latin America	0.03 (0.01, 0.1)	59.68 (16.81, 193.31)	0.04 (0.01, 0.14)	59.96 (16.44, 196.75)	0.47 (−7.92, 8.25)
Caribbean	0.02 (0, 0.06)	51.22 (14.33, 180.48)	0.03 (0.01, 0.09)	51.21 (14.83, 177.77)	−0.02 (−5.26, 4.98)
Central Europe	0.1 (0.03, 0.32)	74.28 (22.46, 241.42)	0.1 (0.03, 0.31)	74.7 (22.49, 248.04)	0.56 (−6.22, 4.36)
Eastern Europe	0.24 (0.08, 0.69)	96.62 (32.94, 283.79)	0.24 (0.09, 0.66)	96.83 (33.12, 286.15)	0.22 (−2.56, 2.55)
Central Asia	0.04 (0.01, 0.15)	66.95 (18.46, 235.41)	0.06 (0.02, 0.23)	66.89 (18.09, 236.36)	−0.09 (−5.06, 3.85)
North Africa and Middle East	0.19 (0.06, 0.58)	67.58 (22.24, 195.24)	0.42 (0.14, 1.25)	67.78 (22.25, 202.06)	0.31 (−9.1, 6.47)
South Asia	0.48 (0.12, 1.88)	50.78 (13.8, 187.01)	0.95 (0.25, 3.47)	50.98 (13.59, 180.49)	0.41 (−5.83, 7.33)
Southeast Asia	0.21 (0.05, 0.82)	50.61 (13.77, 182.95)	0.38 (0.1, 1.32)	51.03 (13.89, 181.26)	0.82 (−4.4, 6.05)
East Asia	0.51 (0.16, 1.74)	41.74 (13.05, 141.21)	0.74 (0.23, 2.25)	43.4 (13.06, 141.04)	3.98 (−3.24, 18.52)
Oceania	0 (0, 0.01)	46.01 (12.99, 167.96)	0.01 (0, 0.02)	46.21 (13.06, 163.83)	0.42 (−8.76, 8.67)
Western Sub-Saharan Africa	0.08 (0.02, 0.31)	56.62 (16.98, 197.34)	0.22 (0.06, 0.79)	57.49 (17.21, 191.19)	1.55 (−3.85, 6.26)
Eastern Sub-Saharan Africa	0.07 (0.02, 0.24)	48.58 (15.71, 159.36)	0.17 (0.05, 0.56)	48.12 (15.48, 151.06)	−0.96 (−5.46, 3.76)
Central Sub-Saharan Africa	0.02 (0.01, 0.08)	53.53 (16.02, 176.2)	0.06 (0.02, 0.21)	53.98 (16.14, 173.91)	0.85 (−6.22, 8.2)
Southern Sub-Saharan Africa	0.03 (0.01, 0.09)	57.49 (17.82, 181.2)	0.05 (0.01, 0.14)	57.04 (17.38, 179.73)	−0.78 (−4.64, 2.54)

UI, uncertainty interval; CI, confidence interval; ASR, age-standardized rate; SDI, Socio-Demographic Index; YLDs, years lived with disability.

Despite the substantial increases in absolute numbers, age-standardized rates remained remarkably stable. The global age-standardized prevalence and incidence rates remained virtually unchanged (decreases of less than 1%), while the YLDs rate showed only a modest decline of approximately 2.3% (Tables 1–3).

### Regional and national burden of TTH and its association with socio-demographic patterns

Regional patterns revealed striking disparities in both burden levels and temporal trends. Among the 21 GBD regions, High-income North America showed exceptionally high burden with prevalence rates exceeding 34,000 per 100,000. East Asia demonstrated the most pronounced increases over the study period, with prevalence rising by 7.6% and incidence by 5.7%. Eastern Europe bore the highest YLDs burden at nearly 97 per 100,000, indicating greater disease severity in this region (Tables 1–3).

**Figure 1:**
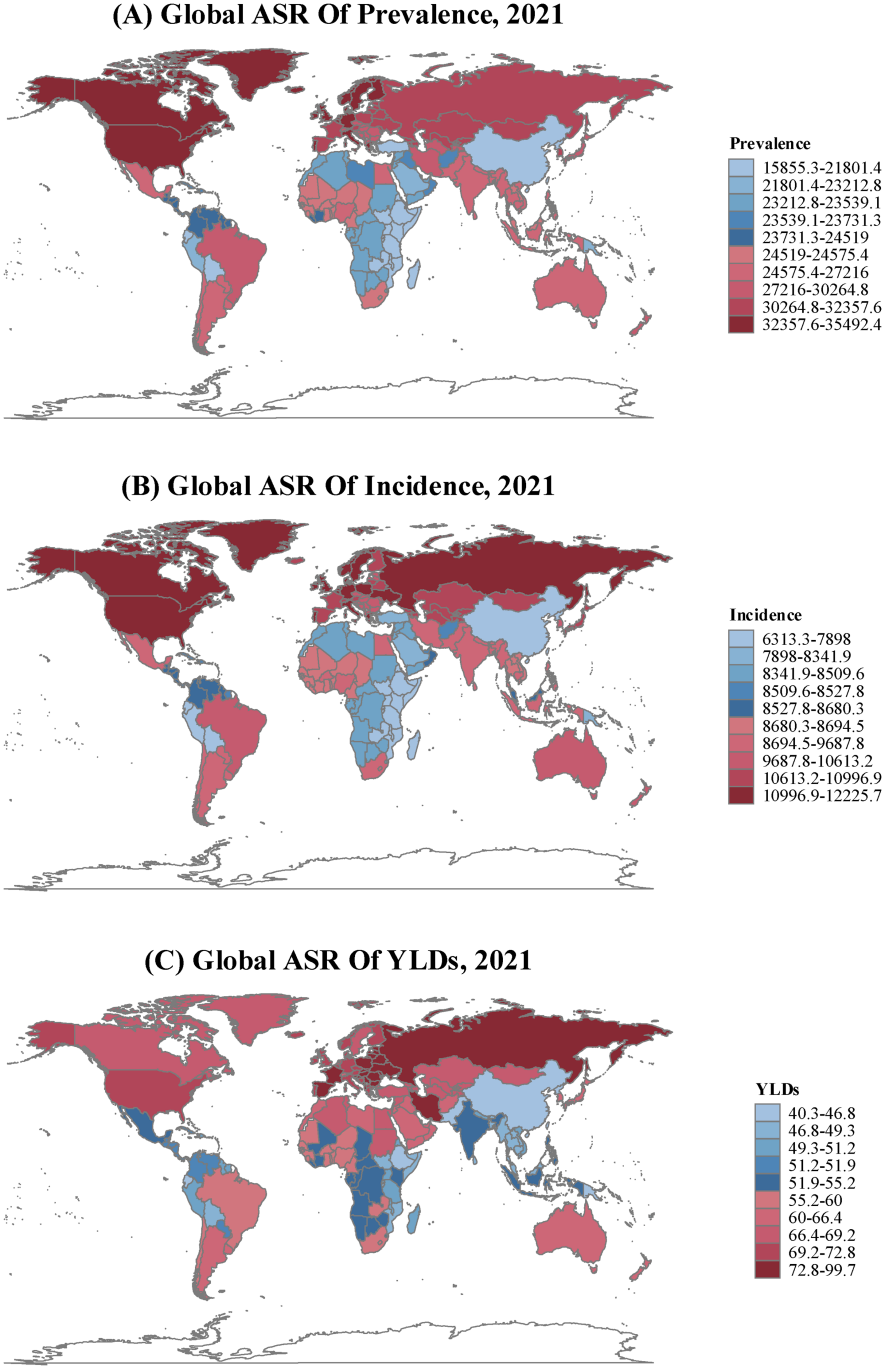
Age-standardized Rate (per 100,000 population) for **(A)** prevalence, **(B)** incidence, and **(C)** years lived with disability (YLDs) of tension-type headache in all age by count and territory during 2021.

At the country level, Nordic countries (particularly Norway) and other high-income Western nations demonstrated the highest TTH burden for both prevalence and incidence, with rates more than double those observed in low-burden countries such as Ethiopia. Eastern European countries, particularly the Russian Federation and Ukraine, showed disproportionately high YLDs rates, suggesting more severe disease impact in these regions (Figure 1; Supplementary Tables S1–S3).

**Figure 2:**
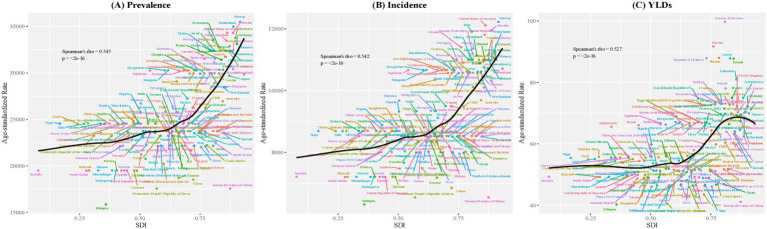
Relationship between ASR of prevalence, incidence, and YLDs and Socio-Demographic Index across 204 countries and territories in 2021. The figure illustrates the relationship between **(A)** age-standardized prevalence rate, **(B)** age-standardized incidence rate, and **(C)** age-standardized YLDs rate per 100,000 population for tension-type headache and the Socio-Demographic Index (SDI) across 204 countries and territories in 2021. The black curves represent LOESS fitting (span = 0.5). Spearman’s correlation coefficient (rho) and corresponding *p*-values are displayed in each panel. Each point represents a country or territory, with different colors used for distinction.

Our analysis across 204 countries revealed moderate positive correlations between SDI and TTH burden indicators: prevalence (*ρ* = 0.545, *p* < 2e-16), incidence (*ρ* = 0.542, *p* < 2e-16), and YLDs (*ρ* = 0.527, *p* < 2e-16). The LOESS curves demonstrated non-linear relationships with burden indicators rising gradually at low SDI levels but accelerating at higher SDI levels, particularly for prevalence and incidence, while YLDs showed a unique pattern with a decline at the highest SDI values (Figure 2).

### Results of age-period-cohort analysis for tension-type headache

**Figure 3:**
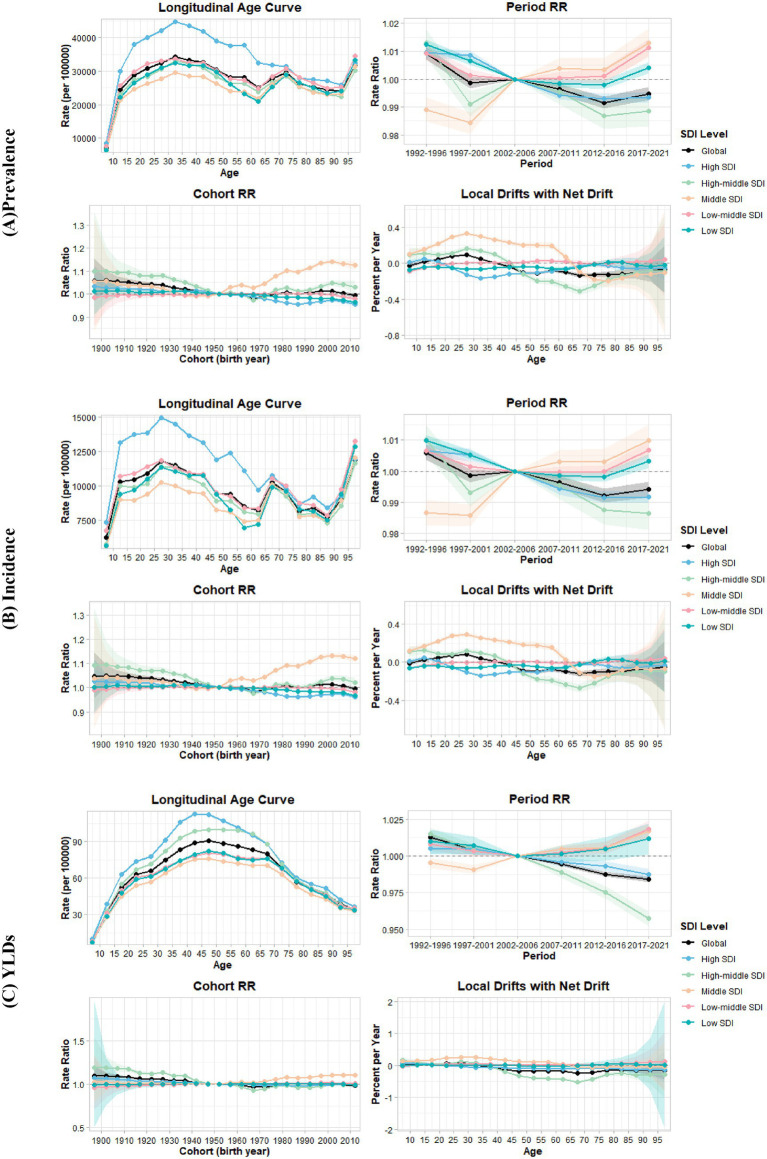
Age-period-cohort analysis of tension-type headache by Socio-Demographic Index (SDI) level, 1990–2021. **(A)** Prevalence. **(B)** Incidence. **(C)** YLDs. Each panel displays longitudinal age curves (top-left), period relative risks (top-right), cohort relative risks (bottom-left), and local drifts with net drift (bottom-right) stratified by SDI level.

The age-period-cohort analysis revealed distinct patterns in the global and different SDI regional burden of TTH from 1990 to 2021. Figure 3 illustrates the longitudinal age curves, period relative risks, cohort relative risks, and local drifts for prevalence, incidence, and YLDs across different SDI levels.

#### Age effects

The longitudinal age curves (Figure 3, top-left panels) revealed distinct age-related patterns of TTH burden. Statistical analysis confirmed significant age effects across all measures and SDI levels (Supplementary Table S6, “All Age Deviations = 0” tests, *p* < 0.001). Globally, TTH prevalence followed a characteristic trajectory: increasing through childhood and early adulthood to peak in the early thirties, declining through middle age, and showing a notable secondary increase in the oldest age groups. This late-life uptick in prevalence is clearly visible in the figure and appears consistent across most SDI regions. Incidence patterns mirrored prevalence but peaked approximately 5 years earlier, while YLDs showed a more gradual increase, peaking in the late forties without the secondary increase observed in the elderly (Supplementary Table S7).

Although all SDI regions exhibited remarkably similar age-related trajectories, High SDI regions consistently demonstrated substantially higher rates compared to other SDI levels for both prevalence and incidence. For prevalence, High SDI regions reached a peak of 44,855 per 100,000 at age 32.5 years. Similarly, incidence in High SDI regions reached approximately 14,500 per 100,000 person-years at age 27.5 years, significantly exceeding the global average and other SDI regions.

#### Period effects

Period effects (Figure 3, top-right panels; Supplementary Table S7) showed subtle but significant temporal variations in disease burden across calendar years from 1992 to 2021. Globally, despite some periodic fluctuations in prevalence and incidence, there was an overall decline in period relative risks for all three measures, with the most pronounced decrease occurring between 1992–1996 and 1997–2001. For prevalence, compared with the reference period 2002–2006, the period RR was 1.009 in 1992–1996, decreasing to 0.999 in 1997–2001, showing minor oscillations in intermediate periods before ultimately declining to 0.995 by 2017–2021. For incidence, a similar pattern was observed, with period RR of 1.006 in 1992–1996, decreasing to 0.999 in 1997–2001, and despite small fluctuations, further declining to by 2017–2021. YLDs demonstrated the most substantial and consistent reductions in period effects, with period RR of 1.013 in 1992–1996, falling sharply to 1.004 in 1997–2001, and steadily continuing its downward trajectory to reach 0.984 by 2017–2021 without the fluctuations seen in prevalence and incidence.

#### Cohort effects

Cohort effects (Figure 3, bottom-left panels; Supplementary Table S7), reflecting variations in disease risk across birth cohorts, showed distinctive patterns across different generations. Globally, earlier birth cohorts (born before 1950) had higher risks of TTH compared to the reference cohort (born in 1952), with cohort RR for prevalence of 1.058 for the 1897 cohort and 1.043 for the 1922 cohort.

The cohort pattern varied substantially across SDI regions. In High and High-middle SDI regions, more recent cohorts (born after 1960) showed progressively lower risks compared to the reference cohort. In contrast, in Middle SDI regions, recent cohorts exhibited higher risks, with cohort RRs increasing from 1.000 in 1952 to 1.125 in 2012 for prevalence. Incidence demonstrated similar patterns to prevalence across all SDI regions, with declining relative risks in High and High-middle SDI regions and increasing relative risks in Middle SDI regions for recent birth cohorts.

The cohort effects were statistically significant across most SDI levels (Supplementary Table S6, “All Cohort Deviations = 0” tests, *p* < 0.001), indicating genuine generational differences in TTH burden. However, in Low-middle and Low SDI regions, cohort effects for YLDs were not statistically significant.

#### Local drift patterns

Local drift patterns (Figure 3, bottom-right panels; Supplementary Table S7), representing age-specific annual percent changes, showed heterogeneity across age groups and SDI levels. Globally, younger age groups (15–35 years) exhibited positive local drifts for prevalence and incidence, while older age groups showed negative local drifts, indicating a shift in the burden toward younger populations.

The most pronounced positive local drifts (>0.2% per year) were observed in Middle SDI regions, with this substantial positive trend persisting across the 20–50 age range, particularly reaching 0.329% per year for prevalence among individuals aged 27.5 years. In contrast, High-middle SDI regions demonstrated substantial negative local drifts (<−0.2% per year) for older age groups, with the trend persisting across the 55–75 age range, where the 67.5-year age group showed the most pronounced decrease, with prevalence declining at −0.309% per year. Similar trends were observed for incidence.

The tests for local drifts (Supplementary Table S6, “All Local Drifts = Net Drift” tests) were statistically significant across all SDI levels (*p* < 0.001 for global and most regions), confirming that the annual changes in TTH burden varied meaningfully by age.

#### Net drift and overall trends

Overall temporal trends confirmed a modest global decline in TTH burden, with annual decreases of approximately 0.05% for prevalence and incidence, and 0.11% for YLDs. The statistical tests for net drift (Supplementary Table S6) were highly significant (*p* < 0.001) for all three measures, confirming a real decreasing trend in TTH burden globally. However, this global improvement masked concerning regional disparities: Middle SDI regions showed significant annual increases of approximately 0.1% across all measures, representing a trajectory opposite to global trends.

### Frontier analysis of TTH burden

**Figure 4:**
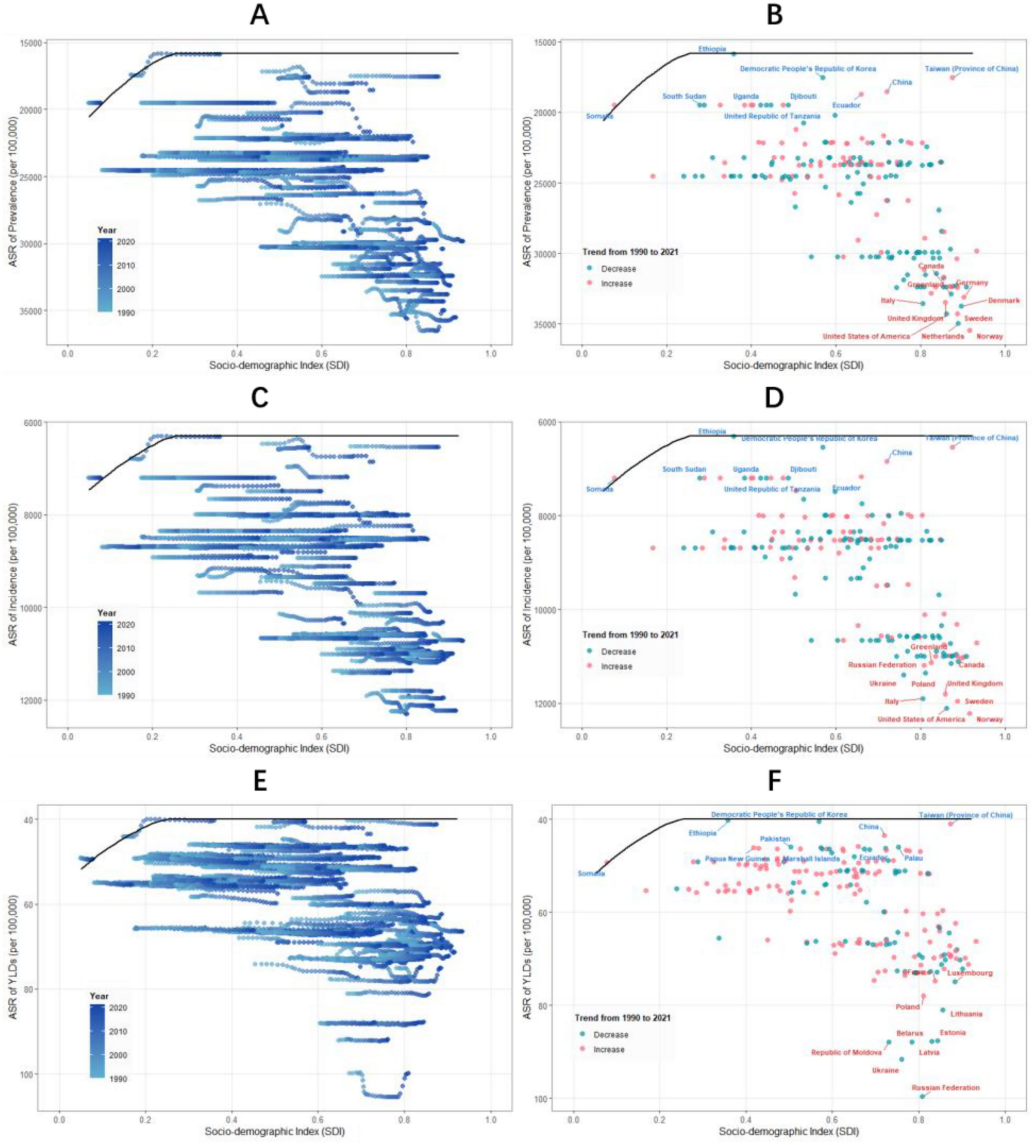
Frontier analysis of global across different Socio-Demographic Index (SDI) levels, 1990–2021. Panels show the frontier analysis results for prevalence **(A,B)**, incidence **(C,D)**, and years lived with disability (YLDs) **(E,F)**. Left panels **(A,C,E)** display temporal transitions of country-specific ASRs from 1990 to 2021, with color gradient indicating the year progression (light blue to dark blue). The frontier line is shown in black, representing the best achievable performance at each SDI level. Right panels **(B,D,F)** illustrate the 2021 status of each country relative to the frontier curve, with dots colored by their directional change since 1990 (blue: decrease; red: increase). Labels highlight countries with the largest gaps from the frontier (red text) and those achieving closest proximity to the frontier (blue text), representing the least and most efficient performers, respectively. All rates are presented per 100,000 population.

Using data from 1990 to 2021, and based on prevalence, incidence, YLDs, and SDI, frontier analysis was conducted to explore the potential improvement space for TTH burden considering national and regional development levels (Figure 4; Supplementary Table S8). The frontier analysis revealed striking disparities in TTH management efficiency. For prevalence, many high-income Western countries, particularly in Northern Europe and North America, showed substantial gaps between their actual burden and the theoretical minimum achievable at their SDI level. In contrast, several East Asian countries and some African nations operated near the efficiency frontier, suggesting more effective prevention or management strategies relative to their development level.

Similar patterns emerged for incidence, with high-income Western nations showing the greatest improvement potential. The YLDs analysis revealed a distinct geographical pattern, with Eastern European countries demonstrating the largest gaps from optimal performance, suggesting particular challenges in managing disease severity in this region. Consistently across all metrics, certain East Asian countries demonstrated remarkable efficiency in minimizing TTH burden relative to their SDI level.

## Discussion

Our study based on the GBD 2021 database comprehensively analyzes the global burden of TTH from 1990 to 2021 and its relationship with socio-demographic factors. Through multi-dimensional analyses across global, regional (5 SDI regions and 21 GBD regions), and national (204 countries and territories) levels, we have derived several important findings that reveal the current status, trends, and complex relationship between TTH burden and socioeconomic development.

### TTH remains a major global public health challenge

Despite modest decreases in age-standardized rates, the absolute number of people with TTH reached 2.01 billion in 2021, representing a 56.4% increase from 1990. Among all detailed disease classifications globally, TTH ranks second in prevalence, and even first in high SDI regions, nearly one-quarter of the global population is affected. From a disease burden perspective, while individual TTH cases typically present with relatively mild symptoms (disability weight of approximately 0.037) (7), the condition’s extraordinarily high prevalence creates a substantial collective impact, generated 4.6 million YLDs in 2021. Beyond this disability burden measured by YLDs, TTH also incurs substantial indirect costs primarily through lost productivity due to absenteeism and, perhaps more significantly, presenteeism (reduced efficiency while working) (16, 17). From a public health perspective, the massive absolute burden suggests that even small improvements in TTH prevention or management could yield substantial population-level benefits, highlighting the urgent need for scalable interventions.

### Complex relationship between socioeconomic development and TTH burden

Our research reveals a moderate positive correlation between TTH burden and SDI. High SDI regions demonstrated substantially higher burden across all metrics compared to global averages. The LOESS curve further illustrates the non-linear nature of this relationship, with TTH burden growth rate accelerating in high SDI regions.

This association likely reflects the combined influence of multiple socioeconomic factors. Rasmussen’s epidemiological research suggests that high headache prevalence in industrialized societies may be related to lifestyle and environmental factors (18). Jensen and Stovner propose that socioeconomic development might influence headache epidemiological patterns through various pathways, including stress levels, healthcare-seeking behaviors, and disease perception changes (9). TTH burden accelerates rather than declines at high SDI levels, suggesting development brings new risk factors.

### Middle-aged adults are at highest risk, with a trend toward younger age groups

Our age-period-cohort analysis reveals critical shifts in TTH epidemiology. Globally, TTH prevalence peaks at approximately 32.5 years, followed by a gradual decline and a secondary increase in elderly populations (90 + years). This age distribution pattern exhibits remarkable consistency across all five SDI regions, suggesting that the fundamental biological mechanisms underlying TTH may transcend socioeconomic, cultural, and developmental boundaries.

Local drift analysis reveals significant age-specific temporal variations in TTH prevalence. At the global level, younger age cohorts (15–35 years) demonstrate positive local drift values, while older groups exhibit negative drift, indicating a progressive shift of the TTH burden toward younger populations. This phenomenon is particularly pronounced in Middle SDI regions, with substantial positive trends (>0.2% per year) persisting across the 20–50 age range, most notably reaching 0.329% per year for prevalence among individuals aged 27.5 years. In contrast, High-middle SDI regions show substantial negative local drifts (<−0.2% per year) for older age groups (55–75 years), with the most pronounced decrease observed in the 67.5-year age group. Statistical analysis confirms these age-specific changes are significant across all SDI levels, validating that TTH burden shifts vary meaningfully by age group.

Cohort effect analysis further corroborates this epidemiological shift toward younger populations. In middle SDI regions, birth cohorts preceding 1950 demonstrate lower TTH risk relative to the 1952 reference cohort, whereas recent cohorts (e.g., 2012 birth cohort) exhibit an elevated risk ratio of 1.125. Conversely, high SDI and high-middle SDI regions display an inverse pattern, characterized by progressively declining risk ratios in recent cohorts. These dynamic age-related patterns align with observations documented by Crystal and Robbins in their comprehensive review of TTH epidemiology (8).

These findings carry significant public health implications, suggesting that TTH prevention and intervention strategies should prioritize middle-aged adults, with particular emphasis on younger populations in middle SDI regions. This targeted approach is consistent with the longitudinal findings reported by Lyngberg et al. (19), highlighting the importance of age-specific intervention strategies in TTH management.

### High SDI countries bear the highest TTH burden

The global TTH burden exhibits a distinctive geographical gradient closely aligned with socioeconomic development. High-income North America exemplified this pattern, with prevalence rates exceeding 34,000 per 100,000—nearly 40% above the global average—and the highest incidence rates among all regions. This stark contrast becomes even more pronounced when compared to sub-Saharan Africa, where prevalence rates were less than 40% of those in North America, highlighting the profound disparities in TTH burden across development levels. Country-level data further support this pattern. The top ten countries in global prevalence are all high SDI countries, with Norway, the Netherlands, and the United States ranking in the top three. For incidence, Norway, the United States, and Sweden similarly occupy the top positions. This highly concentrated burden distribution suggests an intrinsic connection between TTH and highly developed societies.

APC analysis results further confirm this observation. The longitudinal age curves clearly show that for all three indicators (prevalence, incidence, and YLDs), the curves for high SDI regions (blue line) consistently remain in the highest position, significantly above other SDI level regions. This pattern of consistently high burden across all age groups indicates that TTH is a widespread and persistent health issue in high SDI environments.

This high TTH burden in high SDI regions may be associated with multiple factors. Stovner and Andree’s European headache research indicates that lifestyle characteristics in developed countries (such as high work pressure and sleep deprivation) are closely related to high headache prevalence (20). Hagen et al. further found that multiple factors in modern lifestyles, including lack of physical activity, increased psychological stress, and poor sleep habits, are important risk factors for TTH (21). Additionally, more comprehensive medical systems and greater health awareness in high SDI countries may also lead to higher diagnosis and reporting rates.

Notably, significant variations exist within high SDI regions. For example, despite both being high-income regions, East Asian high-income countries (such as Japan and South Korea) have significantly lower TTH burdens than Western high-income countries, a difference that may reflect the importance of cultural factors and social organizational models, as shown by Wang (22). This regional heterogeneity provides important clues for understanding the sociocultural determinants of TTH.

### TTH burden shows an increasing trend in middle SDI regions

A key finding of this study is that while global age-standardized TTH rates show a slight downward trend (net drift −0.056% per year), middle SDI regions exhibit a clear upward trend, with substantial increases across all burden indicators (prevalence, incidence, and YLDs) between 1990 and 2021.

This upward trend is particularly evident in specific regions. East Asia (primarily China) shows the largest growth in prevalence (7.57%), followed by Andean Latin America (1.38%) and Tropical Latin America (0.29%). Yu et al. also documented an increase in headache burden in China (23), indicating that this trend has been confirmed in multiple independent studies.

APC analysis provides multi-dimensional evidence for the increasing TTH burden in middle SDI regions. The period effect analysis reveals a striking contrast: while high SDI regions show declining temporal trends, middle SDI regions demonstrate progressively increasing relative risks over the study period, providing robust evidence for the rising TTH burden in middle SDI regions.

More predictive is the cohort effect analysis which reveals a concerning generational pattern in middle SDI regions: younger birth cohorts show progressively higher TTH risk, contrasting sharply with the declining risk observed in recent cohorts from high SDI regions. This divergent cohort pattern suggests that the TTH burden in middle SDI regions may continue to escalate as these higher-risk younger generations age, consistent with the trend of increasing headache prevalence observed by Mbewe et al. in developing countries (24).

Local drift analysis shows that the annual growth rate of TTH prevalence in young age groups in middle SDI regions is higher than in other age groups and other SDI regions. This age-specific change echoes the phenomenon of increasing headache prevalence among young people in rapidly developing Asian economies reported by Peng et al. (25).

These findings on increasing TTH burden in Middle SDI regions have significant implications for global public health, suggesting that these regions require special attention for TTH prevention and management strategies. The World Health Organization’s Atlas of Headache Disorders (26) similarly emphasizes the need for targeted headache management approaches in developing regions undergoing rapid socioeconomic transitions.

### Frontier analysis provides direction for improving TTH management

To our knowledge, this study is the first to apply frontier analysis at a global level to systematically evaluate the improvement potential of TTH burden relative to the Socio-Demographic Index. This innovative approach quantifies the deviation of each country’s TTH burden from the theoretically achievable minimum level for its SDI level, not only revealing improvement space but also providing insights into optimal social models under different SDI backgrounds.

Prevalence frontier analysis (Figure 4; Supplementary Table S8) shows that high SDI countries such as Norway, the Netherlands, and the United States have the largest room for improvement. In contrast, some East Asian countries such as Taiwan and China, as well as parts of Africa, demonstrate smaller deviations. Incidence frontier analysis results also show a similar pattern, with high SDI countries again having the largest improvement space: Norway, the United States, and Sweden rank in the top three. East Asian and African region countries show minimal deviations, such as China and Somalia. These consistent prevalence and incidence frontier analysis results reinforce our understanding of the improvement potential in high SDI countries. Jacobs et al. propose in their health economics research that such efficiency analysis can provide a basis for optimizing resource allocation (27). YLDs frontier analysis shows that Eastern European countries such as the Russian Federation and Ukraine have the largest YLDs improvement space. Lebedeva et al. also highlighted the specific challenges facing headache management in Russia (28), consistent with our findings.

These frontier analysis results suggest that high SDI does not necessarily lead to high TTH burden; certain social organizational models may achieve lower TTH burden at equivalent development levels. Takeshima et al.’s Japanese study demonstrates the relatively low headache prevalence in East Asia (29), providing clues for exploring protective factors. These findings offer important reference points for TTH prevention and management strategies across different SDI contexts.

### Limitation

Our study has several important limitations. First, while the GBD study employs sophisticated methods including the Bayesian meta-regression tool DisMod-MR 2.1 to address data gaps and generate consistent estimates, the observed differences in TTH burden across SDI levels are likely influenced by both data availability and systematic reporting biases. In high SDI countries, well-developed healthcare systems and greater health awareness may lead to higher diagnosis and reporting rates, potentially overestimating TTH burden. Conversely, low SDI countries may experience substantial underreporting due to limited healthcare access and lower prioritization of non-life-threatening conditions. Additionally, diagnostic challenges inherent to TTH—particularly distinguishing it from mild or chronic migraine—introduce classification errors that vary by healthcare context. Although GBD methodology attempts to adjust for these biases through standardization and modeling approaches, complete elimination of reporting heterogeneity across diverse healthcare contexts remains challenging, and the estimates in data-sparse regions rely more heavily on modeling assumptions that may not fully capture local epidemiological patterns.

Second, our methodological approach has inherent constraints. The disability weights used for YLDs calculations represent population averages and may not adequately capture the heterogeneous impact of TTH on individual patients or distinguish between episodic and chronic subtypes. Additionally, our ecological study design describes epidemiological patterns but cannot establish causal relationships or evaluate specific interventions, limiting our ability to make definitive recommendations about modifiable risk factors or prevention strategies.

## Conclusion

Our comprehensive analysis of global TTH burden from 1990 to 2021 reveals four major findings. First, TTH remains one of the world’s most prevalent conditions, affecting over 2 billion people—a quarter of humanity—with its burden continuing to grow in absolute terms despite modest improvements in age-standardized rates. Second, we identified a complex, non-linear relationship between socioeconomic development and TTH burden, with prevalence paradoxically accelerating at higher SDI levels rather than declining. Third, our age-period-cohort analysis uncovered divergent temporal trajectories: while high SDI regions show encouraging declines, middle SDI regions face escalating burden, particularly among younger cohorts who demonstrate progressively higher risk. Fourth, frontier analysis revealed substantial heterogeneity in performance, with several East Asian nations achieving remarkably low TTH rates despite resource constraints, while many wealthy countries significantly underperform relative to their capabilities.

### Implications for health policy and practice

These findings carry profound implications for global health strategy. The massive prevalence of TTH, combined with its substantial societal impact through lost productivity, suggests that even modest improvements in prevention or management could yield transformative population-level benefits. The paradoxical relationship between development and TTH burden indicates that modernization introduces specific risk factors—chronic stress, sleep disruption, sedentary lifestyles—that require proactive intervention through comprehensive workplace and community programs.

The divergent trajectories between SDI regions represent a critical policy challenge. Middle-income countries face a narrow window for intervention before today’s high-risk youth cohorts age into peak prevalence years. Meanwhile, the success of efficient performers demonstrates that high TTH burden is not an inevitable consequence of development, suggesting that cultural approaches and healthcare delivery innovations may be more important than healthcare expenditure alone. These insights emphasize the necessity of developing differentiated TTH prevention and management strategies for different contexts, consistent with Steiner et al.’s call regarding the global burden of headache (30).

### Future directions

Research priorities should include: (1) investigating specific protective factors in high-performing countries through comparative effectiveness studies; (2) developing and testing scalable interventions tailored to different SDI contexts, as demonstrated by Holroyd et al. in intervention research (31); (3) exploring the mechanisms linking socioeconomic development to TTH burden; and (4) establishing robust surveillance systems in data-sparse regions.

The persistent global burden of TTH, combined with emerging epidemiological transitions and proven potential for improvement, demands coordinated action. By learning from efficient performers and targeting interventions to specific developmental contexts, substantial reductions in the global TTH burden are achievable within existing resource constraints.

## Data Availability

The original contributions presented in the study are included in the article/Supplementary material, further inquiries can be directed to the corresponding author.
