# Supplementary material for: Global burden trends of tension-type headache, 1990–2021: socio-demographic patterns, age-period-cohort effects, and frontier analysis from the GBD 2021 study
Source: Front Neurol. 2025 Jul 16;16:1629025. doi: 10.3389/fneur.2025.1629025 (PMC12308845; doi:10.3389/fneur.2025.1629025)
Supplement: Supplementary file 1 [file Table_1.DOCX]

Catalog

[**Figure S1. Heatmap of the most detailed causes in the GBD 2021 database by (A) prevalence and (B) incidence across global and 5 SDI regions, 2021.** 2](#_Toc196227183)

[**Table S1. ASRs of Prevalence in 204 Countries and Territories, 2021** 3](#_Toc196227184)

[**Table S2. ASR of Incidence in 204 Countries and Territories, 2021** 14](#_Toc196227185)

[**Table S3. ASR of YLDs in 204 Countries and Territories, 2021** 23](#_Toc196227186)

[**Table S4. ASPR, ASIR, APYR and SDI in 204 Countries and Territories, 2021** 32](#_Toc196227187)

[**Table S5. Net Drift values (percent change per year) for prevalence, incidence, and YLDs of tension-type headache by SDI level, 1990-2021.** 40](#_Toc196227188)

[**Table S6. Statistical tests for Age-Period-Cohort effects for prevalence, incidence, and YLDs of tension-type headache by SDI level, 1990-2021.** 41](#_Toc196227189)

[**Table S7. Estimated parameters from Age-Period-Cohort analysis for prevalence, incidence, and YLDs of tension-type headache by SDI level, 1990-2021.** 51](#_Toc196227190)

[**Table S8. Age-Standardized Rates and Frontier Analysis Results for Tension-Type Headache by Country, 2021** 69](#_Toc196227191)

# **Figure S1. Heatmap of the most detailed causes in the GBD 2021 database by (A) prevalence and (B) incidence across global and 5 SDI regions, 2021.**


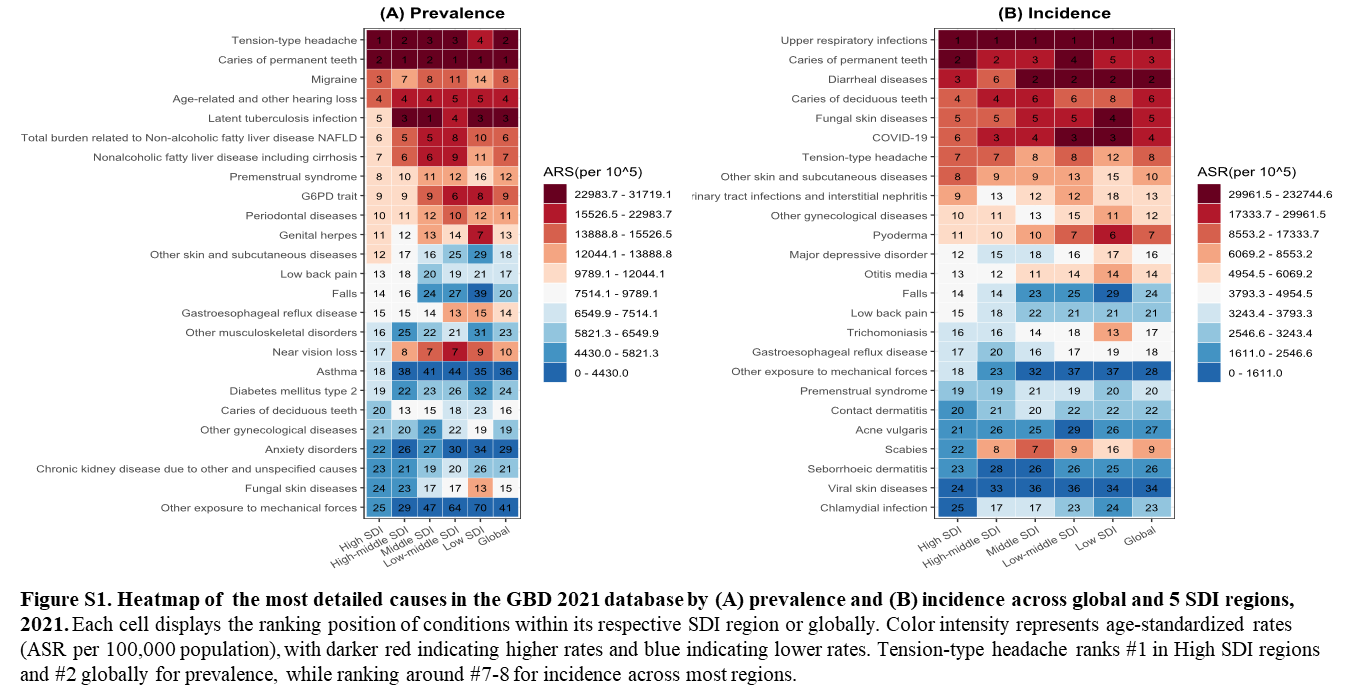


# **Table S1. ASRs of Prevalence in 204 Countries and Territories, 2021**

| **Rank** | **Country** | **Prevalence per 100,000 (95% UI)** |
| --- | --- | --- |
| 1 | Norway | 35492.44 (31718.03, 39481.91) |
| 2 | Netherlands | 34984.91 (30123.30, 39942.65) |
| 3 | United States of America | 34320.58 (30762.93, 38348.91) |
| 4 | Sweden | 34297.27 (30701.03, 38326.02) |
| 5 | Denmark | 33764.78 (29477.82, 38545.83) |
| 6 | Italy | 33572.31 (29964.27, 37532.66) |
| 7 | United Kingdom | 33487.02 (29942.12, 37299.08) |
| 8 | Germany | 33107.73 (28985.82, 37680.86) |
| 9 | Canada | 32911.94 (28790.69, 37247.53) |
| 10 | Greenland | 32837.19 (28691.37, 37179.33) |
| 11 | San Marino | 32430.84 (28280.21, 36962.56) |
| 12 | Portugal | 32429.23 (28267.44, 37011.93) |
| 13 | Cyprus | 32411.95 (28254.01, 37014.80) |
| 14 | Monaco | 32411.21 (28250.66, 36983.37) |
| 15 | Greece | 32408.70 (28249.91, 36985.43) |
| 16 | Ireland | 32406.02 (28242.61, 36967.09) |
| 17 | Israel | 32400.54 (28232.98, 37007.16) |
| 18 | Belgium | 32396.92 (28239.73, 36903.59) |
| 19 | Finland | 32383.20 (28196.63, 36941.96) |
| 20 | Malta | 32376.71 (28207.45, 36976.61) |
| 21 | Iceland | 32368.67 (28203.02, 36964.11) |
| 22 | Andorra | 32352.91 (28179.03, 36861.21) |
| 23 | Luxembourg | 32279.96 (28111.40, 36828.77) |
| 24 | Ukraine | 31872.74 (28432.01, 35612.60) |
| 25 | Austria | 31759.67 (27635.41, 36479.78) |
| 26 | Lithuania | 31729.84 (27557.16, 36200.23) |
| 27 | Spain | 31535.63 (27408.60, 36010.06) |
| 28 | Poland | 31533.24 (28135.30, 35109.35) |
| 29 | France | 31418.48 (27367.37, 36027.39) |
| 30 | Russian Federation | 31147.27 (27732.82, 35055.65) |
| 31 | Republic of Korea | 30412.35 (26422.95, 34445.55) |
| 32 | Belarus | 30340.65 (26426.41, 34728.35) |
| 33 | Latvia | 30338.15 (26401.78, 34724.16) |
| 34 | Estonia | 30335.97 (26372.25, 34759.24) |
| 35 | Republic of Moldova | 30334.65 (26376.91, 34792.29) |
| 36 | Kazakhstan | 30289.04 (26200.20, 34606.08) |
| 37 | Mongolia | 30281.81 (26182.47, 34537.74) |
| 38 | Kyrgyzstan | 30280.71 (26238.16, 34539.85) |
| 39 | Armenia | 30276.13 (26208.46, 34510.57) |
| 40 | Georgia | 30274.88 (26214.32, 34527.44) |
| 41 | Uzbekistan | 30269.39 (26182.01, 34531.50) |
| 42 | Azerbaijan | 30266.52 (26167.63, 34565.73) |
| 43 | Turkmenistan | 30262.16 (26149.75, 34491.20) |
| 44 | Tajikistan | 30255.25 (26151.48, 34514.61) |
| 45 | Hungary | 29976.16 (26046.38, 34344.52) |
| 46 | Slovakia | 29962.05 (26021.42, 34279.58) |
| 47 | Bosnia and Herzegovina | 29958.94 (26050.58, 34343.35) |
| 48 | Romania | 29957.47 (26033.17, 34318.15) |
| 49 | Montenegro | 29955.13 (26063.60, 34324.21) |
| 50 | Bulgaria | 29955.10 (26079.24, 34340.31) |
| 51 | Croatia | 29953.92 (26171.06, 34212.29) |
| 52 | Czechia | 29944.15 (26025.75, 34247.32) |
| 53 | Albania | 29929.00 (25981.73, 34255.02) |
| 54 | Serbia | 29928.17 (26039.72, 34277.48) |
| 55 | Slovenia | 29927.39 (26032.45, 34300.82) |
| 56 | North Macedonia | 29923.19 (25998.04, 34281.34) |
| 57 | Switzerland | 29829.20 (25864.17, 34171.76) |
| 58 | Japan | 29705.70 (26600.44, 33406.16) |
| 59 | Brazil | 29095.41 (25755.93, 32674.17) |
| 60 | Brunei Darussalam | 28928.90 (25259.30, 33374.45) |
| 61 | Singapore | 28481.36 (24802.46, 32730.41) |
| 62 | New Zealand | 28452.74 (25420.01, 31819.62) |
| 63 | Iran (Islamic Republic of) | 27244.00 (24278.11, 30516.37) |
| 64 | Australia | 26964.15 (23424.48, 30875.06) |
| 65 | Pakistan | 26707.03 (23686.83, 30189.12) |
| 66 | Paraguay | 26396.49 (22489.21, 30459.46) |
| 67 | Uruguay | 26281.32 (22811.17, 30202.33) |
| 68 | Argentina | 26270.96 (22765.49, 30229.03) |
| 69 | Chile | 26244.34 (22762.50, 30152.43) |
| 70 | Egypt | 25845.30 (22389.77, 29457.26) |
| 71 | Philippines | 25783.43 (22658.70, 28879.95) |
| 72 | Indonesia | 25774.93 (22651.39, 28888.59) |
| 73 | Nigeria | 25749.89 (22935.55, 28955.65) |
| 74 | India | 25298.04 (22421.85, 28418.03) |
| 75 | Mexico | 25068.15 (22145.35, 28379.13) |
| 76 | Bhutan | 24804.70 (21191.89, 28828.32) |
| 77 | Bangladesh | 24771.44 (21160.00, 28698.10) |
| 78 | Myanmar | 24636.82 (21101.14, 28221.48) |
| 79 | Cambodia | 24628.97 (21118.66, 28298.40) |
| 80 | Sri Lanka | 24617.15 (21076.29, 28209.67) |
| 81 | Nepal | 24613.27 (20956.66, 28522.19) |
| 82 | Thailand | 24604.29 (21090.89, 28170.64) |
| 83 | Viet Nam | 24581.87 (21070.37, 28211.88) |
| 84 | Mauritius | 24573.74 (21101.82, 28230.33) |
| 85 | Lao People's Democratic Republic | 24566.31 (21043.86, 28192.99) |
| 86 | Burkina Faso | 24561.03 (21288.84, 28183.65) |
| 87 | Guinea-Bissau | 24557.15 (21284.73, 28211.99) |
| 88 | Ghana | 24555.80 (21395.25, 28244.73) |
| 89 | Timor-Leste | 24554.54 (21029.71, 28179.83) |
| 90 | Guinea | 24553.71 (21370.55, 28213.81) |
| 91 | Benin | 24550.25 (21313.39, 28207.78) |
| 92 | Togo | 24549.89 (21280.59, 28200.06) |
| 93 | Mauritania | 24545.61 (21293.83, 28182.49) |
| 94 | Niger | 24545.47 (21310.64, 28192.14) |
| 95 | Gambia | 24545.08 (21361.16, 28152.06) |
| 96 | Cameroon | 24535.48 (21257.96, 28206.50) |
| 97 | Sierra Leone | 24535.33 (21243.63, 28088.13) |
| 98 | Mali | 24533.94 (21295.62, 28154.45) |
| 99 | Sao Tome and Principe | 24532.89 (21314.61, 28143.98) |
| 100 | Chad | 24531.89 (21286.05, 28145.73) |
| 101 | Cabo Verde | 24530.58 (21224.81, 28129.77) |
| 102 | South Africa | 24528.71 (21597.65, 27670.03) |
| 103 | Senegal | 24528.55 (21253.47, 28163.79) |
| 104 | Malaysia | 24519.06 (21054.43, 28100.40) |
| 105 | Liberia | 24518.93 (21275.98, 28135.12) |
| 106 | Côte d'Ivoire | 24501.69 (21266.52, 28122.42) |
| 107 | Seychelles | 24479.93 (20988.85, 28096.01) |
| 108 | Maldives | 24278.21 (20796.04, 27850.09) |
| 109 | El Salvador | 23763.66 (20315.35, 27732.21) |
| 110 | Venezuela (Bolivarian Republic of) | 23756.85 (20348.51, 27721.57) |
| 111 | Bahamas | 23748.98 (20277.44, 27683.56) |
| 112 | Guatemala | 23746.01 (20330.54, 27721.19) |
| 113 | Honduras | 23745.73 (20303.70, 27662.54) |
| 114 | Costa Rica | 23742.75 (20324.40, 27688.44) |
| 115 | Bermuda | 23741.33 (20314.57, 27681.36) |
| 116 | Puerto Rico | 23740.88 (20293.03, 27699.66) |
| 117 | United States Virgin Islands | 23738.92 (20271.47, 27701.87) |
| 118 | Saint Kitts and Nevis | 23738.23 (20299.17, 27639.28) |
| 119 | Guyana | 23737.03 (20309.99, 27669.00) |
| 120 | Haiti | 23736.29 (20295.01, 27691.16) |
| 121 | Nicaragua | 23736.01 (20304.72, 27668.23) |
| 122 | Barbados | 23735.71 (20308.91, 27698.63) |
| 123 | Suriname | 23735.03 (20316.99, 27674.49) |
| 124 | Colombia | 23732.07 (20281.02, 27695.91) |
| 125 | Antigua and Barbuda | 23728.21 (20304.30, 27670.43) |
| 126 | Dominican Republic | 23723.71 (20297.53, 27676.18) |
| 127 | Trinidad and Tobago | 23723.31 (20288.39, 27647.82) |
| 128 | Jamaica | 23722.86 (20278.59, 27641.13) |
| 129 | Belize | 23720.48 (20282.44, 27677.94) |
| 130 | Saint Lucia | 23718.12 (20280.99, 27604.87) |
| 131 | Cuba | 23717.59 (20266.41, 27686.51) |
| 132 | Panama | 23715.99 (20290.22, 27673.82) |
| 133 | Dominica | 23714.53 (20305.39, 27621.44) |
| 134 | Grenada | 23710.78 (20299.51, 27645.52) |
| 135 | Saint Vincent and the Grenadines | 23704.30 (20282.52, 27651.33) |
| 136 | Oman | 23580.68 (20316.56, 27360.91) |
| 137 | Afghanistan | 23578.38 (20370.41, 27419.47) |
| 138 | Bahrain | 23559.15 (20276.42, 27372.69) |
| 139 | Kuwait | 23548.04 (20333.26, 27100.04) |
| 140 | Iraq | 23545.34 (20329.95, 27325.55) |
| 141 | Libya | 23542.45 (20280.99, 27335.21) |
| 142 | United Arab Emirates | 23538.75 (20260.58, 27285.49) |
| 143 | Lebanon | 23534.28 (20277.28, 27364.41) |
| 144 | Yemen | 23532.90 (20325.51, 27360.79) |
| 145 | Jordan | 23532.88 (20286.44, 27306.80) |
| 146 | Palestine | 23528.29 (20340.14, 27301.35) |
| 147 | Qatar | 23526.77 (20234.10, 27313.55) |
| 148 | Tunisia | 23526.35 (20237.55, 27305.75) |
| 149 | Morocco | 23523.54 (20290.25, 27327.70) |
| 150 | Syrian Arab Republic | 23519.33 (20330.81, 27265.47) |
| 151 | Algeria | 23517.68 (20261.90, 27285.93) |
| 152 | Sudan | 23470.17 (20239.49, 27298.86) |
| 153 | Gabon | 23246.38 (20103.78, 27058.36) |
| 154 | Lesotho | 23245.90 (20101.00, 27116.00) |
| 155 | Zimbabwe | 23244.83 (20099.99, 27100.86) |
| 156 | Angola | 23244.25 (20099.21, 27102.22) |
| 157 | Namibia | 23242.32 (20096.61, 27070.93) |
| 158 | Eswatini | 23237.60 (20087.83, 27057.01) |
| 159 | Central African Republic | 23237.18 (20070.37, 27092.47) |
| 160 | Botswana | 23234.87 (20092.77, 27059.10) |
| 161 | Congo | 23228.55 (20084.17, 27089.59) |
| 162 | Democratic Republic of the Congo | 23228.07 (20075.14, 27082.61) |
| 163 | Equatorial Guinea | 23202.58 (20046.12, 27051.00) |
| 164 | Saudi Arabia | 22434.33 (19416.48, 25712.82) |
| 165 | Kiribati | 22252.77 (19103.63, 25648.91) |
| 166 | Cook Islands | 22226.16 (19076.54, 25677.08) |
| 167 | Tonga | 22214.78 (19083.60, 25655.10) |
| 168 | Nauru | 22210.98 (19041.95, 25678.01) |
| 169 | Niue | 22182.00 (19028.10, 25635.05) |
| 170 | Vanuatu | 22181.20 (19000.24, 25603.16) |
| 171 | Fiji | 22173.81 (19006.87, 25611.05) |
| 172 | Micronesia (Federated States of) | 22170.51 (19030.32, 25572.25) |
| 173 | Solomon Islands | 22166.62 (18998.91, 25630.53) |
| 174 | Tokelau | 22166.23 (19015.06, 25605.67) |
| 175 | Guam | 22159.46 (19016.54, 25596.05) |
| 176 | American Samoa | 22152.00 (19000.43, 25529.97) |
| 177 | Marshall Islands | 22147.43 (18992.73, 25579.00) |
| 178 | Samoa | 22147.15 (19008.41, 25571.11) |
| 179 | Tuvalu | 22139.86 (19005.66, 25560.21) |
| 180 | Papua New Guinea | 22129.08 (18979.41, 25528.06) |
| 181 | Northern Mariana Islands | 22111.12 (18957.51, 25510.09) |
| 182 | Palau | 22029.78 (18930.24, 25399.16) |
| 183 | Peru | 21862.34 (18598.50, 25330.55) |
| 184 | Türkiye | 21659.04 (18704.53, 24951.95) |
| 185 | Zambia | 21241.53 (18033.19, 24636.04) |
| 186 | Kenya | 20758.45 (18405.81, 23390.11) |
| 187 | Bolivia (Plurinational State of) | 20238.68 (17219.44, 23429.02) |
| 188 | Eritrea | 19509.71 (16758.07, 22699.27) |
| 189 | Somalia | 19507.50 (16769.16, 22724.72) |
| 190 | Rwanda | 19506.59 (16777.88, 22734.08) |
| 191 | Comoros | 19504.26 (16758.88, 22700.34) |
| 192 | Mozambique | 19504.07 (16798.44, 22687.27) |
| 193 | Malawi | 19504.05 (16781.33, 22726.61) |
| 194 | Burundi | 19503.76 (16765.40, 22710.83) |
| 195 | Madagascar | 19501.66 (16787.27, 22717.30) |
| 196 | Uganda | 19499.97 (16760.76, 22741.15) |
| 197 | United Republic of Tanzania | 19499.54 (16791.68, 22722.55) |
| 198 | Djibouti | 19489.09 (16726.21, 22664.00) |
| 199 | South Sudan | 19484.86 (16752.15, 22695.16) |
| 200 | Ecuador | 18708.72 (15939.35, 21726.07) |
| 201 | China | 18525.07 (16380.87, 20958.70) |
| 202 | Democratic People's Republic of Korea | 17560.91 (14930.47, 20516.62) |
| 203 | Taiwan (Province of China) | 17557.81 (14915.25, 20515.45) |
| 204 | Ethiopia | 15855.33 (13637.60, 18206.87) |
| *Abbreviations: ASR, Age-Standardized Rate; UI, uncertainty interval. Note: Countries and territories are ranked from highest to lowest based on ASR of Prevalence in 2021. Prevalence rates are presented per 100,000 population.* | | |

# **Table S2. ASR of Incidence in 204 Countries and Territories, 2021**

| **Rank** | **Country** | **Incidence per 100,000 (95% UI)** |
| --- | --- | --- |
| 1 | Norway | 12225.67 (10673.08, 13778.10) |
| 2 | United States of America | 12110.68 (10550.58, 13520.49) |
| 3 | Sweden | 11957.24 (10503.70, 13393.96) |
| 4 | Italy | 11907.30 (10379.71, 13336.30) |
| 5 | United Kingdom | 11812.17 (10329.04, 13276.29) |
| 6 | Ukraine | 11405.07 (9946.67, 12812.49) |
| 7 | Poland | 11365.29 (9873.17, 12708.66) |
| 8 | Russian Federation | 11200.64 (9779.76, 12544.09) |
| 9 | Canada | 11154.29 (9726.08, 12617.51) |
| 10 | Greenland | 11136.98 (9714.67, 12594.05) |
| 11 | Netherlands | 11114.04 (9423.86, 12741.29) |
| 12 | Denmark | 11052.66 (9475.52, 12514.31) |
| 13 | Germany | 11012.92 (9501.26, 12540.99) |
| 14 | Portugal | 11006.84 (9472.07, 12441.46) |
| 15 | San Marino | 11005.36 (9469.08, 12460.00) |
| 16 | Greece | 11001.95 (9465.27, 12446.06) |
| 17 | Cyprus | 11000.77 (9453.23, 12466.52) |
| 18 | Monaco | 11000.46 (9455.21, 12458.07) |
| 19 | Ireland | 11000.32 (9468.17, 12461.84) |
| 20 | Israel | 11000.06 (9471.90, 12441.52) |
| 21 | Belgium | 10998.81 (9469.81, 12445.79) |
| 22 | Finland | 10996.08 (9447.74, 12448.71) |
| 23 | Malta | 10993.94 (9458.99, 12439.10) |
| 24 | Iceland | 10991.29 (9457.04, 12473.55) |
| 25 | Andorra | 10985.72 (9464.72, 12425.65) |
| 26 | Luxembourg | 10969.42 (9428.47, 12453.80) |
| 27 | Austria | 10935.23 (9446.25, 12425.36) |
| 28 | Spain | 10896.83 (9420.95, 12313.98) |
| 29 | France | 10893.20 (9425.25, 12363.67) |
| 30 | Lithuania | 10764.37 (9260.52, 12195.19) |
| 31 | Japan | 10726.09 (9392.75, 12010.89) |
| 32 | Switzerland | 10713.80 (9241.01, 12024.54) |
| 33 | Kazakhstan | 10669.89 (9188.59, 12092.77) |
| 34 | Mongolia | 10668.43 (9174.36, 12071.62) |
| 35 | Kyrgyzstan | 10667.65 (9189.87, 12093.22) |
| 36 | Georgia | 10666.34 (9194.51, 12099.01) |
| 37 | Armenia | 10665.43 (9186.84, 12093.01) |
| 38 | Uzbekistan | 10664.57 (9160.34, 12066.56) |
| 39 | Turkmenistan | 10664.40 (9184.30, 12096.20) |
| 40 | Azerbaijan | 10663.63 (9179.89, 12082.49) |
| 41 | Tajikistan | 10660.56 (9178.00, 12058.03) |
| 42 | Belarus | 10613.19 (9181.14, 11995.05) |
| 43 | Latvia | 10613.17 (9181.93, 11978.28) |
| 44 | Estonia | 10612.76 (9186.97, 11995.59) |
| 45 | Republic of Moldova | 10611.66 (9177.32, 11960.03) |
| 46 | Croatia | 10594.00 (9213.94, 11944.99) |
| 47 | Hungary | 10579.89 (9135.77, 11922.77) |
| 48 | Slovakia | 10576.74 (9135.52, 11920.58) |
| 49 | Romania | 10575.83 (9131.70, 11915.94) |
| 50 | Bosnia and Herzegovina | 10575.51 (9138.10, 11929.93) |
| 51 | Bulgaria | 10575.40 (9134.59, 11930.69) |
| 52 | Montenegro | 10573.96 (9116.30, 11920.21) |
| 53 | Czechia | 10572.78 (9122.21, 11924.83) |
| 54 | Slovenia | 10568.36 (9125.70, 11919.72) |
| 55 | Albania | 10567.46 (9117.97, 11922.52) |
| 56 | North Macedonia | 10567.30 (9129.20, 11916.74) |
| 57 | Serbia | 10566.41 (9126.68, 11909.54) |
| 58 | Brazil | 10349.78 (9086.49, 11557.13) |
| 59 | New Zealand | 10345.43 (9039.55, 11584.93) |
| 60 | Republic of Korea | 10316.74 (8886.98, 11632.65) |
| 61 | Brunei Darussalam | 10119.39 (8824.77, 11441.92) |
| 62 | Singapore | 10098.07 (8749.10, 11429.86) |
| 63 | Australia | 9688.93 (8381.32, 10881.89) |
| 64 | Pakistan | 9677.34 (8501.33, 10819.06) |
| 65 | Iran (Islamic Republic of) | 9497.90 (8336.87, 10642.26) |
| 66 | Uruguay | 9488.89 (8282.52, 10695.83) |
| 67 | Argentina | 9486.56 (8269.25, 10698.64) |
| 68 | Chile | 9478.38 (8267.13, 10675.95) |
| 69 | Paraguay | 9347.45 (8172.81, 10551.99) |
| 70 | Philippines | 9339.72 (8191.73, 10475.69) |
| 71 | Indonesia | 9337.80 (8194.24, 10476.47) |
| 72 | India | 9333.37 (8160.49, 10426.38) |
| 73 | Nigeria | 9322.13 (8144.24, 10453.29) |
| 74 | Mexico | 9118.53 (7947.83, 10211.65) |
| 75 | South Africa | 8928.23 (7739.98, 10058.37) |
| 76 | Bhutan | 8924.36 (7795.91, 10044.27) |
| 77 | Bangladesh | 8912.69 (7778.73, 10033.24) |
| 78 | Nepal | 8883.19 (7746.28, 9966.79) |
| 79 | Egypt | 8804.60 (7688.76, 9907.78) |
| 80 | Myanmar | 8702.37 (7575.45, 9816.76) |
| 81 | Cambodia | 8699.40 (7573.13, 9807.80) |
| 82 | Sri Lanka | 8696.58 (7579.93, 9778.11) |
| 83 | Burkina Faso | 8695.69 (7561.25, 9807.86) |
| 84 | Guinea-Bissau | 8694.21 (7573.14, 9811.01) |
| 85 | Ghana | 8693.43 (7572.97, 9826.66) |
| 86 | Benin | 8693.18 (7575.33, 9810.97) |
| 87 | Guinea | 8693.01 (7570.03, 9811.23) |
| 88 | Thailand | 8692.88 (7581.47, 9793.50) |
| 89 | Gambia | 8692.36 (7582.08, 9799.32) |
| 90 | Togo | 8692.03 (7568.34, 9815.29) |
| 91 | Mauritania | 8691.92 (7575.66, 9810.15) |
| 92 | Niger | 8691.83 (7577.17, 9806.73) |
| 93 | Sierra Leone | 8690.35 (7573.49, 9812.64) |
| 94 | Sao Tome and Principe | 8689.77 (7569.74, 9820.97) |
| 95 | Cabo Verde | 8689.40 (7576.89, 9815.90) |
| 96 | Cameroon | 8689.35 (7579.62, 9817.74) |
| 97 | Chad | 8688.96 (7571.44, 9811.92) |
| 98 | Mali | 8688.86 (7567.37, 9809.35) |
| 99 | Senegal | 8687.01 (7564.44, 9799.78) |
| 100 | Liberia | 8685.74 (7564.59, 9791.25) |
| 101 | Viet Nam | 8684.61 (7554.78, 9778.89) |
| 102 | Mauritius | 8683.30 (7554.43, 9758.84) |
| 103 | Côte d'Ivoire | 8681.40 (7561.54, 9798.26) |
| 104 | Lao People's Democratic Republic | 8681.17 (7574.98, 9766.55) |
| 105 | Timor-Leste | 8679.38 (7552.65, 9791.48) |
| 106 | Malaysia | 8667.01 (7537.01, 9753.18) |
| 107 | Seychelles | 8653.34 (7525.17, 9746.10) |
| 108 | Maldives | 8591.78 (7477.04, 9695.04) |
| 109 | El Salvador | 8536.06 (7400.40, 9640.95) |
| 110 | Venezuela (Bolivarian Republic of) | 8534.61 (7401.66, 9638.73) |
| 111 | Bahamas | 8532.98 (7416.18, 9597.46) |
| 112 | Guatemala | 8531.58 (7429.07, 9634.71) |
| 113 | Oman | 8531.38 (7411.79, 9604.98) |
| 114 | Honduras | 8530.79 (7412.44, 9626.47) |
| 115 | Costa Rica | 8530.68 (7402.44, 9610.72) |
| 116 | Bermuda | 8530.66 (7401.55, 9628.90) |
| 117 | Saint Kitts and Nevis | 8530.01 (7395.13, 9615.52) |
| 118 | Puerto Rico | 8529.73 (7393.48, 9630.41) |
| 119 | Guyana | 8528.98 (7397.39, 9618.82) |
| 120 | Haiti | 8528.88 (7401.42, 9631.73) |
| 121 | Nicaragua | 8528.86 (7407.67, 9614.09) |
| 122 | Barbados | 8528.50 (7420.00, 9622.13) |
| 123 | United States Virgin Islands | 8528.50 (7400.80, 9620.27) |
| 124 | Colombia | 8527.89 (7409.28, 9614.23) |
| 125 | Suriname | 8527.48 (7412.90, 9624.30) |
| 126 | Antigua and Barbuda | 8525.86 (7439.75, 9622.62) |
| 127 | Dominican Republic | 8525.75 (7394.58, 9626.36) |
| 128 | Trinidad and Tobago | 8525.21 (7416.09, 9623.38) |
| 129 | United Arab Emirates | 8525.01 (7395.83, 9616.75) |
| 130 | Jamaica | 8524.37 (7397.21, 9620.14) |
| 131 | Belize | 8524.31 (7397.03, 9617.42) |
| 132 | Saint Lucia | 8523.51 (7394.53, 9620.58) |
| 133 | Cuba | 8523.41 (7388.64, 9602.59) |
| 134 | Panama | 8522.63 (7388.53, 9617.94) |
| 135 | Dominica | 8522.13 (7403.63, 9614.94) |
| 136 | Bahrain | 8521.98 (7395.34, 9609.98) |
| 137 | Grenada | 8521.26 (7393.57, 9608.85) |
| 138 | Afghanistan | 8520.94 (7419.07, 9605.02) |
| 139 | Saint Vincent and the Grenadines | 8518.92 (7402.72, 9619.79) |
| 140 | Qatar | 8512.83 (7375.23, 9608.41) |
| 141 | Kuwait | 8511.29 (7367.94, 9543.88) |
| 142 | Iraq | 8509.36 (7413.70, 9610.86) |
| 143 | Libya | 8509.21 (7420.31, 9575.55) |
| 144 | Lebanon | 8508.61 (7409.89, 9573.11) |
| 145 | Jordan | 8508.47 (7413.41, 9566.32) |
| 146 | Yemen | 8505.13 (7404.02, 9608.76) |
| 147 | Palestine | 8503.56 (7404.43, 9589.80) |
| 148 | Morocco | 8502.99 (7420.04, 9565.79) |
| 149 | Tunisia | 8502.17 (7407.55, 9582.68) |
| 150 | Algeria | 8501.42 (7414.05, 9549.33) |
| 151 | Syrian Arab Republic | 8488.87 (7417.56, 9553.56) |
| 152 | Sudan | 8485.20 (7409.02, 9537.62) |
| 153 | Lesotho | 8351.10 (7239.68, 9396.37) |
| 154 | Gabon | 8350.94 (7228.01, 9399.13) |
| 155 | Angola | 8350.80 (7233.26, 9406.80) |
| 156 | Namibia | 8350.69 (7236.15, 9425.38) |
| 157 | Zimbabwe | 8350.66 (7233.68, 9400.99) |
| 158 | Central African Republic | 8348.66 (7229.75, 9407.49) |
| 159 | Eswatini | 8348.36 (7225.74, 9407.77) |
| 160 | Botswana | 8347.82 (7237.70, 9398.02) |
| 161 | Democratic Republic of the Congo | 8346.57 (7238.13, 9388.10) |
| 162 | Congo | 8346.37 (7232.79, 9392.89) |
| 163 | Equatorial Guinea | 8339.00 (7245.20, 9385.54) |
| 164 | Saudi Arabia | 8335.68 (7222.65, 9386.49) |
| 165 | Türkiye | 8176.28 (7162.75, 9202.95) |
| 166 | Kiribati | 8031.74 (6942.67, 9013.17) |
| 167 | Cook Islands | 8024.80 (6927.91, 9009.63) |
| 168 | Tonga | 8020.27 (6943.91, 9006.66) |
| 169 | Nauru | 8017.98 (6939.21, 9000.08) |
| 170 | Vanuatu | 8009.40 (6932.17, 8999.32) |
| 171 | Niue | 8008.38 (6930.55, 8993.09) |
| 172 | Fiji | 8006.38 (6928.72, 8986.41) |
| 173 | Solomon Islands | 8004.91 (6918.25, 8991.01) |
| 174 | Micronesia (Federated States of) | 8004.74 (6921.21, 8976.73) |
| 175 | Tokelau | 8003.90 (6925.69, 8986.83) |
| 176 | Guam | 8001.53 (6932.61, 8970.16) |
| 177 | American Samoa | 8000.52 (6924.86, 8984.30) |
| 178 | Marshall Islands | 7998.83 (6904.19, 8985.57) |
| 179 | Samoa | 7998.22 (6923.60, 8984.96) |
| 180 | Tuvalu | 7994.48 (6913.53, 8976.37) |
| 181 | Papua New Guinea | 7993.20 (6926.35, 8988.98) |
| 182 | Northern Mariana Islands | 7985.96 (6906.69, 8977.68) |
| 183 | Palau | 7958.90 (6882.70, 8946.73) |
| 184 | Peru | 7756.04 (6700.51, 8690.13) |
| 185 | Kenya | 7660.13 (6695.48, 8658.23) |
| 186 | Bolivia (Plurinational State of) | 7487.86 (6467.41, 8412.55) |
| 187 | Zambia | 7475.24 (6506.11, 8488.04) |
| 188 | Eritrea | 7208.67 (6253.57, 8132.62) |
| 189 | Burundi | 7207.77 (6256.73, 8128.68) |
| 190 | Somalia | 7207.65 (6258.20, 8127.00) |
| 191 | Comoros | 7206.99 (6255.62, 8126.49) |
| 192 | Rwanda | 7206.92 (6251.80, 8142.09) |
| 193 | Malawi | 7206.44 (6251.75, 8132.95) |
| 194 | Madagascar | 7206.09 (6248.15, 8127.40) |
| 195 | Mozambique | 7205.81 (6255.21, 8130.82) |
| 196 | Uganda | 7204.82 (6263.45, 8131.72) |
| 197 | United Republic of Tanzania | 7204.82 (6254.35, 8135.27) |
| 198 | Djibouti | 7203.74 (6256.47, 8134.75) |
| 199 | South Sudan | 7200.00 (6257.34, 8124.61) |
| 200 | Ecuador | 7181.50 (6229.27, 8124.31) |
| 201 | China | 6851.13 (5957.52, 7729.63) |
| 202 | Democratic People's Republic of Korea | 6543.77 (5699.03, 7420.68) |
| 203 | Taiwan (Province of China) | 6542.13 (5702.44, 7447.93) |
| 204 | Ethiopia | 6313.30 (5505.60, 7182.86) |
| *Abbreviations: ASR, Age-Standardized Rate; UI, uncertainty interval. Note: Countries and territories are ranked from highest to lowest based on ASR of Incidence in 2021. Incidence rates are presented per 100,000 population.* | | |

# **Table S3. ASR of YLDs in 204 Countries and Territories, 2021**

| **Rank** | **Country** | **YLDs per 100,000 (95% UI)** |
| --- | --- | --- |
| 1 | Russian Federation | 99.72 (34.79, 288.43) |
| 2 | Ukraine | 91.72 (29.95, 277.45) |
| 3 | Belarus | 88.01 (27.48, 269.39) |
| 4 | Republic of Moldova | 87.90 (28.60, 267.41) |
| 5 | Latvia | 87.86 (28.02, 271.83) |
| 6 | Estonia | 87.75 (28.49, 271.39) |
| 7 | Lithuania | 81.03 (24.53, 266.39) |
| 8 | Poland | 78.06 (23.62, 254.86) |
| 9 | Luxembourg | 74.98 (21.88, 262.84) |
| 10 | France | 74.92 (20.53, 253.03) |
| 11 | Iran (Islamic Republic of) | 74.74 (23.75, 226.59) |
| 12 | Spain | 73.69 (20.11, 267.06) |
| 13 | Slovakia | 73.13 (21.84, 243.89) |
| 14 | Serbia | 73.08 (21.71, 242.74) |
| 15 | Croatia | 73.08 (21.40, 245.11) |
| 16 | Romania | 73.08 (21.09, 241.38) |
| 17 | Hungary | 73.07 (22.02, 241.63) |
| 18 | Montenegro | 73.04 (21.40, 242.42) |
| 19 | Albania | 72.99 (21.94, 242.13) |
| 20 | Slovenia | 72.89 (21.48, 241.72) |
| 21 | Bulgaria | 72.81 (21.03, 240.78) |
| 22 | Czechia | 72.75 (21.07, 244.46) |
| 23 | Bosnia and Herzegovina | 72.69 (21.99, 238.70) |
| 24 | North Macedonia | 72.65 (21.86, 241.46) |
| 25 | United Kingdom | 72.27 (19.01, 266.15) |
| 26 | Germany | 72.21 (19.84, 254.64) |
| 27 | Italy | 71.46 (18.10, 278.39) |
| 28 | Norway | 71.25 (16.88, 282.32) |
| 29 | Austria | 71.22 (19.01, 267.60) |
| 30 | Denmark | 70.36 (16.92, 266.48) |
| 31 | United States of America | 70.32 (18.05, 258.29) |
| 32 | San Marino | 70.07 (18.33, 260.01) |
| 33 | Monaco | 69.94 (17.66, 261.90) |
| 34 | Cyprus | 69.91 (18.09, 259.22) |
| 35 | Israel | 69.80 (17.93, 260.03) |
| 36 | Portugal | 69.80 (17.90, 255.82) |
| 37 | Greece | 69.65 (17.78, 256.03) |
| 38 | Ireland | 69.58 (17.98, 257.28) |
| 39 | Belgium | 69.42 (17.77, 261.14) |
| 40 | Iceland | 69.37 (18.13, 256.86) |
| 41 | Finland | 69.26 (18.66, 256.04) |
| 42 | Malta | 69.25 (17.73, 258.90) |
| 43 | Andorra | 69.04 (17.06, 256.96) |
| 44 | Egypt | 68.90 (21.37, 214.42) |
| 45 | Sweden | 68.31 (17.15, 259.89) |
| 46 | Netherlands | 68.16 (15.73, 292.36) |
| 47 | Canada | 67.31 (16.62, 250.64) |
| 48 | Armenia | 67.18 (17.75, 237.49) |
| 49 | Syrian Arab Republic | 67.11 (22.11, 198.34) |
| 50 | Kyrgyzstan | 67.08 (17.79, 236.20) |
| 51 | Azerbaijan | 67.04 (17.96, 237.05) |
| 52 | Kazakhstan | 67.00 (18.26, 234.79) |
| 53 | Georgia | 66.84 (18.54, 236.28) |
| 54 | Türkiye | 66.80 (22.60, 181.22) |
| 55 | Uzbekistan | 66.79 (18.09, 236.66) |
| 56 | Mongolia | 66.78 (17.86, 234.70) |
| 57 | Tajikistan | 66.73 (17.78, 235.56) |
| 58 | Turkmenistan | 66.70 (18.01, 235.97) |
| 59 | Algeria | 66.65 (22.19, 198.76) |
| 60 | Tunisia | 66.63 (21.45, 197.96) |
| 61 | Lebanon | 66.55 (21.53, 197.91) |
| 62 | Greenland | 66.44 (16.91, 248.99) |
| 63 | Sudan | 66.39 (21.52, 196.51) |
| 64 | Switzerland | 66.36 (18.13, 241.18) |
| 65 | Palestine | 66.30 (21.32, 194.36) |
| 66 | Morocco | 66.29 (21.79, 195.43) |
| 67 | Libya | 66.22 (21.98, 198.78) |
| 68 | Yemen | 66.08 (21.04, 196.21) |
| 69 | Iraq | 65.89 (21.67, 196.45) |
| 70 | Jordan | 65.89 (21.20, 196.51) |
| 71 | Afghanistan | 65.57 (21.59, 192.90) |
| 72 | Oman | 64.81 (20.31, 199.85) |
| 73 | Japan | 64.56 (17.87, 215.33) |
| 74 | Bahrain | 64.36 (20.45, 199.60) |
| 75 | New Zealand | 64.05 (17.98, 203.70) |
| 76 | Saudi Arabia | 63.98 (20.70, 190.64) |
| 77 | United Arab Emirates | 63.46 (19.54, 199.12) |
| 78 | Qatar | 63.20 (19.84, 194.19) |
| 79 | Kuwait | 63.16 (20.37, 199.63) |
| 80 | Republic of Korea | 62.39 (16.05, 220.52) |
| 81 | Brunei Darussalam | 60.40 (15.85, 212.63) |
| 82 | Australia | 60.38 (17.08, 201.33) |
| 83 | Argentina | 60.02 (16.42, 197.26) |
| 84 | Uruguay | 60.02 (17.29, 199.22) |
| 85 | Chile | 59.83 (16.42, 195.09) |
| 86 | Nigeria | 59.81 (18.28, 193.08) |
| 87 | Singapore | 59.68 (16.32, 215.97) |
| 88 | South Africa | 57.93 (17.66, 181.26) |
| 89 | Zambia | 57.44 (18.97, 168.10) |
| 90 | Brazil | 56.12 (14.52, 206.29) |
| 91 | Ghana | 55.95 (16.09, 183.71) |
| 92 | Mauritania | 55.94 (16.54, 186.05) |
| 93 | Togo | 55.90 (16.69, 188.90) |
| 94 | Burkina Faso | 55.77 (16.50, 194.55) |
| 95 | Sao Tome and Principe | 55.73 (16.38, 182.53) |
| 96 | Cabo Verde | 55.63 (15.82, 181.65) |
| 97 | Guinea-Bissau | 55.56 (16.43, 190.31) |
| 98 | Benin | 55.55 (16.36, 192.62) |
| 99 | Guinea | 55.55 (16.14, 191.14) |
| 100 | Cameroon | 55.45 (15.99, 185.59) |
| 101 | Niger | 55.43 (16.85, 200.21) |
| 102 | Gambia | 55.39 (15.93, 185.49) |
| 103 | Senegal | 55.39 (15.65, 186.64) |
| 104 | Sierra Leone | 55.22 (16.62, 190.67) |
| 105 | Mexico | 55.21 (16.31, 185.41) |
| 106 | Mali | 55.09 (15.80, 191.57) |
| 107 | Côte d'Ivoire | 55.02 (16.18, 187.22) |
| 108 | Chad | 54.94 (15.72, 194.73) |
| 109 | Namibia | 54.53 (16.10, 173.96) |
| 110 | Gabon | 54.44 (16.62, 172.33) |
| 111 | Angola | 54.40 (16.16, 175.27) |
| 112 | Zimbabwe | 54.40 (16.06, 173.80) |
| 113 | Liberia | 54.33 (15.77, 187.96) |
| 114 | Equatorial Guinea | 54.15 (16.33, 171.47) |
| 115 | Botswana | 54.15 (16.47, 173.90) |
| 116 | Congo | 54.11 (16.27, 174.12) |
| 117 | Eswatini | 54.04 (16.18, 170.97) |
| 118 | Central African Republic | 53.86 (16.61, 175.81) |
| 119 | Democratic Republic of the Congo | 53.83 (16.16, 175.79) |
| 120 | Lesotho | 53.73 (15.99, 172.20) |
| 121 | Kenya | 53.60 (17.21, 160.48) |
| 122 | Indonesia | 52.56 (14.33, 182.43) |
| 123 | Philippines | 52.38 (14.40, 181.68) |
| 124 | India | 51.88 (14.09, 180.56) |
| 125 | Bermuda | 51.87 (14.67, 177.46) |
| 126 | El Salvador | 51.86 (14.38, 177.90) |
| 127 | Puerto Rico | 51.78 (14.57, 173.42) |
| 128 | Costa Rica | 51.76 (14.73, 176.28) |
| 129 | Bahamas | 51.73 (14.74, 173.66) |
| 130 | Colombia | 51.70 (14.80, 178.16) |
| 131 | Barbados | 51.68 (15.06, 176.23) |
| 132 | United States Virgin Islands | 51.63 (14.56, 176.87) |
| 133 | Jamaica | 51.53 (14.73, 176.65) |
| 134 | Venezuela (Bolivarian Republic of) | 51.53 (14.35, 178.50) |
| 135 | Antigua and Barbuda | 51.47 (14.57, 176.27) |
| 136 | Cuba | 51.43 (14.73, 178.36) |
| 137 | Panama | 51.32 (14.04, 174.90) |
| 138 | Nicaragua | 51.30 (14.12, 178.18) |
| 139 | Saint Kitts and Nevis | 51.29 (14.43, 174.20) |
| 140 | Paraguay | 51.27 (13.48, 189.90) |
| 141 | Belize | 51.26 (14.20, 176.18) |
| 142 | Honduras | 51.26 (15.08, 178.88) |
| 143 | Guatemala | 51.17 (13.68, 178.14) |
| 144 | Dominican Republic | 51.15 (14.43, 176.94) |
| 145 | Trinidad and Tobago | 51.15 (14.38, 176.82) |
| 146 | Dominica | 51.14 (14.24, 177.07) |
| 147 | Saint Lucia | 51.11 (14.44, 175.99) |
| 148 | Suriname | 51.11 (14.78, 175.78) |
| 149 | Grenada | 50.96 (13.85, 175.79) |
| 150 | Saint Vincent and the Grenadines | 50.94 (14.16, 175.74) |
| 151 | Guyana | 50.81 (14.16, 174.47) |
| 152 | Haiti | 50.76 (14.80, 181.30) |
| 153 | Nepal | 50.69 (13.73, 179.81) |
| 154 | Rwanda | 50.12 (16.33, 156.69) |
| 155 | Madagascar | 49.90 (15.70, 155.40) |
| 156 | Uganda | 49.87 (16.11, 156.20) |
| 157 | United Republic of Tanzania | 49.85 (15.66, 155.61) |
| 158 | Comoros | 49.80 (15.90, 152.81) |
| 159 | Malawi | 49.79 (15.95, 156.61) |
| 160 | Eritrea | 49.61 (15.78, 155.01) |
| 161 | Peru | 49.43 (14.39, 177.17) |
| 162 | Viet Nam | 49.38 (13.27, 179.79) |
| 163 | Somalia | 49.29 (16.22, 165.05) |
| 164 | Thailand | 49.25 (13.32, 179.20) |
| 165 | Burundi | 49.23 (15.59, 160.83) |
| 166 | Mozambique | 49.23 (15.92, 154.38) |
| 167 | South Sudan | 49.21 (15.55, 159.30) |
| 168 | Djibouti | 49.20 (15.56, 154.04) |
| 169 | Sri Lanka | 49.12 (12.83, 178.26) |
| 170 | Myanmar | 49.08 (12.90, 179.41) |
| 171 | Malaysia | 48.99 (12.72, 177.09) |
| 172 | Lao People's Democratic Republic | 48.96 (13.18, 178.69) |
| 173 | Cambodia | 48.94 (12.92, 181.13) |
| 174 | Seychelles | 48.90 (12.80, 176.96) |
| 175 | Bangladesh | 48.87 (12.84, 175.36) |
| 176 | Mauritius | 48.84 (13.03, 179.19) |
| 177 | Timor-Leste | 48.69 (12.77, 175.55) |
| 178 | Bhutan | 48.58 (12.90, 178.81) |
| 179 | Maldives | 48.16 (12.91, 175.80) |
| 180 | Bolivia (Plurinational State of) | 47.30 (14.68, 171.92) |
| 181 | Guam | 46.94 (12.88, 162.53) |
| 182 | Cook Islands | 46.86 (13.37, 163.57) |
| 183 | Tonga | 46.81 (13.25, 165.36) |
| 184 | Northern Mariana Islands | 46.70 (13.06, 164.02) |
| 185 | Niue | 46.66 (13.29, 165.53) |
| 186 | Tokelau | 46.52 (12.78, 165.00) |
| 187 | Nauru | 46.50 (12.93, 166.08) |
| 188 | Kiribati | 46.50 (13.22, 162.16) |
| 189 | Micronesia (Federated States of) | 46.47 (13.22, 163.62) |
| 190 | Samoa | 46.47 (12.44, 163.71) |
| 191 | Tuvalu | 46.46 (13.35, 161.11) |
| 192 | Fiji | 46.41 (12.93, 165.20) |
| 193 | Vanuatu | 46.41 (12.80, 163.75) |
| 194 | Solomon Islands | 46.35 (13.34, 165.99) |
| 195 | American Samoa | 46.26 (12.53, 163.86) |
| 196 | Marshall Islands | 46.15 (13.16, 160.76) |
| 197 | Papua New Guinea | 46.12 (13.03, 165.28) |
| 198 | Pakistan | 46.10 (11.06, 181.63) |
| 199 | Palau | 46.09 (13.02, 161.94) |
| 200 | Ecuador | 45.90 (14.76, 166.78) |
| 201 | China | 43.50 (13.07, 141.34) |
| 202 | Taiwan (Province of China) | 41.13 (12.49, 134.34) |
| 203 | Democratic People's Republic of Korea | 40.51 (11.75, 136.01) |
| 204 | Ethiopia | 40.27 (13.00, 128.45) |
| *Abbreviations: ASR, Age-Standardized RateY; UI, uncertainty interval; YLDs, years lived with disability Note: Countries and territories are ranked from highest to lowest based on age-standardized YLDs rates in 2021. YLDs rates are presented per 100,000 population.* | | |

# **Table S4. ASPR, ASIR, APYR and SDI in 204 Countries and Territories, 2021**

| **SDI** | **location** | **ASPR** | **ASIR** | **ASYR** |
| --- | --- | --- | --- | --- |
| 0.077688109 | Somalia | 19507.49741 | 7207.645192 | 49.28692751 |
| 0.168072774 | Niger | 24545.47104 | 8691.828692 | 55.42745217 |
| 0.240436019 | Chad | 24531.89088 | 8688.963621 | 54.93648967 |
| 0.268579941 | Mali | 24533.93754 | 8688.864903 | 55.08984079 |
| 0.278371125 | South Sudan | 19484.85935 | 7199.999656 | 49.21457614 |
| 0.285118402 | Burkina Faso | 24561.02545 | 8695.693675 | 55.77041194 |
| 0.289374365 | Burundi | 19503.76487 | 7207.766042 | 49.22530693 |
| 0.30916769 | Central African Republic | 23237.17999 | 8348.662967 | 53.86395947 |
| 0.326462614 | Mozambique | 19504.07069 | 7205.814432 | 49.23184677 |
| 0.336401293 | Guinea | 24553.7094 | 8693.009939 | 55.55248189 |
| 0.337199998 | Afghanistan | 23578.37724 | 8520.938228 | 65.57413892 |
| 0.352442452 | Liberia | 24518.93141 | 8685.736865 | 54.33161085 |
| 0.353109621 | Guinea-Bissau | 24557.15298 | 8694.212258 | 55.55881691 |
| 0.358665881 | Sierra Leone | 24535.32986 | 8690.349174 | 55.2186509 |
| 0.358823295 | Ethiopia | 15855.3296 | 6313.304922 | 40.27339142 |
| 0.373486574 | Benin | 24550.24517 | 8693.17788 | 55.54721007 |
| 0.383179849 | Democratic Republic of the Congo | 23228.07268 | 8346.566638 | 53.83286301 |
| 0.384553634 | Malawi | 19504.05047 | 7206.438268 | 49.79145964 |
| 0.400246943 | Madagascar | 19501.66315 | 7206.090306 | 49.89703725 |
| 0.403863943 | Eritrea | 19509.71414 | 7208.670374 | 49.60939323 |
| 0.408054193 | Senegal | 24528.5547 | 8687.013487 | 55.38705633 |
| 0.408533695 | Togo | 24549.88925 | 8692.034513 | 55.89534843 |
| 0.40971416 | Gambia | 24545.0774 | 8692.361269 | 55.38538924 |
| 0.417797443 | Papua New Guinea | 22129.08422 | 7993.1977 | 46.12172061 |
| 0.423261181 | Uganda | 19499.97337 | 7204.820167 | 49.87040256 |
| 0.425941883 | Côte d'Ivoire | 24501.68676 | 8681.400915 | 55.01636823 |
| 0.429360316 | Solomon Islands | 22166.62308 | 8004.91308 | 46.3484581 |
| 0.433174635 | Nepal | 24613.26782 | 8883.193866 | 50.68794394 |
| 0.435588706 | Rwanda | 19506.58729 | 7206.917874 | 50.11525724 |
| 0.444667619 | Timor-Leste | 24554.54287 | 8679.382082 | 48.68667588 |
| 0.446568273 | United Republic of Tanzania | 19499.53993 | 7204.816178 | 49.84808973 |
| 0.448278285 | Haiti | 23736.28955 | 8528.881492 | 50.76345432 |
| 0.450376375 | Yemen | 23532.89604 | 8505.133039 | 66.08003506 |
| 0.453721949 | Angola | 23244.25178 | 8350.797609 | 54.40432017 |
| 0.473062378 | Bhutan | 24804.70007 | 8924.364085 | 48.58360291 |
| 0.473100706 | Vanuatu | 22181.19979 | 8009.395766 | 46.40721173 |
| 0.473621491 | Cambodia | 24628.97489 | 8699.400197 | 48.93503533 |
| 0.473819486 | Zimbabwe | 23244.83211 | 8350.660233 | 54.40483395 |
| 0.475978688 | Comoros | 19504.2583 | 7206.988318 | 49.79696333 |
| 0.479691223 | Cameroon | 24535.4766 | 8689.354891 | 55.45167006 |
| 0.487958371 | Djibouti | 19489.08795 | 7203.741438 | 49.19510636 |
| 0.489136091 | Lao People's Democratic Republic | 24566.31247 | 8681.168794 | 48.95875684 |
| 0.492420885 | Bangladesh | 24771.43538 | 8912.687845 | 48.87315942 |
| 0.4989451 | Mauritania | 24545.60973 | 8691.915958 | 55.94142198 |
| 0.503390833 | Nigeria | 25749.89044 | 9322.133576 | 59.80976259 |
| 0.504028689 | Pakistan | 26707.02729 | 9677.339631 | 46.10168545 |
| 0.505413747 | Sao Tome and Principe | 24532.89183 | 8689.768521 | 55.72625899 |
| 0.505948954 | Zambia | 21241.52666 | 7475.239304 | 57.43787696 |
| 0.510393066 | Lesotho | 23245.90362 | 8351.095759 | 53.72735696 |
| 0.513037248 | Honduras | 23745.73498 | 8530.794452 | 51.25934148 |
| 0.523768077 | Kenya | 20758.45189 | 7660.12867 | 53.6035297 |
| 0.523958472 | Nicaragua | 23736.00754 | 8528.864672 | 51.30276993 |
| 0.527186583 | Kiribati | 22252.76869 | 8031.738135 | 46.49905716 |
| 0.533534539 | Cabo Verde | 24530.57898 | 8689.4047 | 55.62816153 |
| 0.53390084 | Myanmar | 24636.81513 | 8702.372836 | 49.08439565 |
| 0.539972424 | Guatemala | 23746.00989 | 8531.577884 | 51.16535771 |
| 0.541511187 | Tajikistan | 30255.25422 | 10660.55522 | 66.72721933 |
| 0.541949735 | Sudan | 23470.17162 | 8485.197609 | 66.3883572 |
| 0.562698301 | Morocco | 23523.54228 | 8502.988115 | 66.29327528 |
| 0.563775188 | El Salvador | 23763.66103 | 8536.063357 | 51.85932128 |
| 0.56493039 | Ghana | 24555.79629 | 8693.425833 | 55.94732995 |
| 0.569854634 | Democratic People's Republic of Korea | 17560.908 | 6543.766382 | 40.50674034 |
| 0.574091128 | Marshall Islands | 22147.43179 | 7998.83211 | 46.14839124 |
| 0.575401649 | India | 25298.03881 | 9333.372626 | 51.87861851 |
| 0.576620529 | Tuvalu | 22139.85743 | 7994.475374 | 46.4586078 |
| 0.583075236 | Congo | 23228.54597 | 8346.370045 | 54.11344256 |
| 0.585459713 | Eswatini | 23237.604 | 8348.356389 | 54.0355349 |
| 0.587534967 | Micronesia (Federated States of) | 22170.51125 | 8004.741183 | 46.4685162 |
| 0.593392769 | Samoa | 22147.1512 | 7998.215501 | 46.4671267 |
| 0.596513059 | Venezuela (Bolivarian Republic of) | 23756.84675 | 8534.605101 | 51.5318469 |
| 0.599010799 | Bolivia (Plurinational State of) | 20238.67986 | 7487.861356 | 47.30467164 |
| 0.603979328 | Kyrgyzstan | 30280.70587 | 10667.6509 | 67.08160299 |
| 0.606787094 | Egypt | 25845.29764 | 8804.59632 | 68.90405181 |
| 0.610229002 | Belize | 23720.47625 | 8524.309641 | 51.2589918 |
| 0.617564872 | Namibia | 23242.32308 | 8350.688729 | 54.52713805 |
| 0.617621565 | Mongolia | 30281.80571 | 10668.43428 | 66.78229105 |
| 0.619388201 | Dominican Republic | 23723.70841 | 8525.749966 | 51.14974509 |
| 0.623004075 | Syrian Arab Republic | 23519.32591 | 8488.869065 | 67.11094924 |
| 0.625177834 | Nauru | 22210.9778 | 8017.975529 | 46.49947396 |
| 0.626349936 | Tonga | 22214.77941 | 8020.273279 | 46.80682549 |
| 0.627933721 | Viet Nam | 24581.86587 | 8684.614264 | 49.37690481 |
| 0.631011665 | Palestine | 23528.29183 | 8503.563076 | 66.29651034 |
| 0.633665739 | Suriname | 23735.03092 | 8527.47527 | 51.10656382 |
| 0.634691393 | Gabon | 23246.37569 | 8350.944358 | 54.43572061 |
| 0.635718099 | Paraguay | 26396.49447 | 9347.447889 | 51.27326687 |
| 0.637195963 | Saint Vincent and the Grenadines | 23704.30242 | 8518.920769 | 50.94322917 |
| 0.642721629 | Botswana | 23234.87218 | 8347.819723 | 54.15017037 |
| 0.650812335 | Guyana | 23737.03171 | 8528.975642 | 50.80818665 |
| 0.650886627 | Maldives | 24278.21184 | 8591.775375 | 48.1640515 |
| 0.651219329 | Philippines | 25783.42505 | 9339.716298 | 52.38215885 |
| 0.653043887 | Brazil | 29095.40852 | 10349.77507 | 56.11831321 |
| 0.655442913 | Colombia | 23732.07197 | 8527.893613 | 51.69872017 |
| 0.656868336 | Indonesia | 25774.92965 | 9337.801093 | 52.56354181 |
| 0.657857456 | Equatorial Guinea | 23202.58164 | 8339.003529 | 54.14860735 |
| 0.659500924 | Algeria | 23517.68057 | 8501.42427 | 66.65326296 |
| 0.661017053 | Ecuador | 18708.71615 | 7181.50051 | 45.90323567 |
| 0.662054037 | Peru | 21862.34015 | 7756.035505 | 49.42707742 |
| 0.662621694 | Uzbekistan | 30269.38575 | 10664.57221 | 66.7886813 |
| 0.662626231 | Iraq | 23545.33709 | 8509.356756 | 65.89094987 |
| 0.664575304 | Mexico | 25068.14567 | 9118.526498 | 55.2145898 |
| 0.668729864 | Cuba | 23717.58622 | 8523.407818 | 51.42896195 |
| 0.668993028 | Grenada | 23710.78173 | 8521.255956 | 50.95620958 |
| 0.672509735 | Saint Lucia | 23718.12403 | 8523.505445 | 51.11466619 |
| 0.675051631 | Fiji | 22173.80912 | 8006.376429 | 46.41029241 |
| 0.679626598 | South Africa | 24528.7092 | 8928.226971 | 57.93150549 |
| 0.682160776 | Turkmenistan | 30262.16356 | 10664.40023 | 66.70252847 |
| 0.682432216 | Tunisia | 23526.34883 | 8502.170521 | 66.62520858 |
| 0.682547933 | Thailand | 24604.28675 | 8692.881773 | 49.24632882 |
| 0.683263064 | Jamaica | 23722.86309 | 8524.371463 | 51.53244959 |
| 0.686425621 | Tokelau | 22166.22673 | 8003.896084 | 46.52285933 |
| 0.694851274 | Azerbaijan | 30266.51998 | 10663.62829 | 67.03563943 |
| 0.697207398 | Iran (Islamic Republic of) | 27244.00066 | 9497.895528 | 74.73656099 |
| 0.700340477 | Costa Rica | 23742.7452 | 8530.68245 | 51.76023273 |
| 0.701534935 | Sri Lanka | 24617.15204 | 8696.583077 | 49.12198341 |
| 0.701833194 | Armenia | 30276.13426 | 10665.43048 | 67.18444048 |
| 0.706849791 | Albania | 29928.9961 | 10567.45952 | 72.98723942 |
| 0.708864828 | Panama | 23715.9857 | 8522.630046 | 51.32075703 |
| 0.712692673 | Türkiye | 21659.03572 | 8176.283976 | 66.80017475 |
| 0.718260446 | Mauritius | 24573.74331 | 8683.300843 | 48.84384673 |
| 0.719283445 | Uruguay | 26281.31928 | 9488.894612 | 60.01656975 |
| 0.72162976 | China | 18525.06694 | 6851.134225 | 43.49815401 |
| 0.723077893 | Bosnia and Herzegovina | 29958.94067 | 10575.5138 | 72.69158637 |
| 0.723122973 | Argentina | 26270.9643 | 9486.562393 | 60.01574319 |
| 0.723727533 | American Samoa | 22152.00425 | 8000.517936 | 46.25614816 |
| 0.725144495 | Kazakhstan | 30289.04112 | 10669.89002 | 67.00482221 |
| 0.725307227 | Jordan | 23532.88494 | 8508.465816 | 65.89025899 |
| 0.725771399 | Libya | 23542.44872 | 8509.205514 | 66.21977837 |
| 0.72622205 | Niue | 22182.0011 | 8008.376811 | 46.66289743 |
| 0.730150775 | Seychelles | 24479.93002 | 8653.336488 | 48.90470734 |
| 0.732214875 | Republic of Moldova | 30334.65068 | 10611.65676 | 87.90130344 |
| 0.732473604 | Georgia | 30274.87533 | 10666.34321 | 66.84364109 |
| 0.742523828 | Malaysia | 24519.05652 | 8667.010246 | 48.99083758 |
| 0.744151851 | Portugal | 32429.22553 | 11006.84077 | 69.80037794 |
| 0.744746351 | Lebanon | 23534.28422 | 8508.61256 | 66.54825291 |
| 0.746748764 | Barbados | 23735.70663 | 8528.501154 | 51.67580112 |
| 0.746967185 | Dominica | 23714.53216 | 8522.134733 | 51.138073 |
| 0.749886887 | Antigua and Barbuda | 23728.21036 | 8525.858927 | 51.47364158 |
| 0.750629703 | North Macedonia | 29923.18935 | 10567.29988 | 72.65112306 |
| 0.753043204 | Bahrain | 23559.14707 | 8521.97697 | 64.35571594 |
| 0.754046931 | Palau | 22029.78497 | 7958.89668 | 46.09209643 |
| 0.754987055 | Saint Kitts and Nevis | 23738.22721 | 8530.007063 | 51.2911184 |
| 0.760773913 | Ukraine | 31872.73639 | 11405.06894 | 91.72499176 |
| 0.768150939 | Bulgaria | 29955.10064 | 10575.39511 | 72.81010609 |
| 0.768453864 | Romania | 29957.4698 | 10575.83485 | 73.08053356 |
| 0.768763254 | Trinidad and Tobago | 23723.31153 | 8525.208819 | 51.15228192 |
| 0.769283698 | Spain | 31535.62581 | 10896.82765 | 73.68685774 |
| 0.771514716 | Chile | 26244.3418 | 9478.382671 | 59.8322663 |
| 0.771535213 | Northern Mariana Islands | 22111.12432 | 7985.96352 | 46.70164859 |
| 0.773391602 | Oman | 23580.67766 | 8531.380302 | 64.81302681 |
| 0.779109955 | Cook Islands | 22226.15825 | 8024.798682 | 46.86220805 |
| 0.784484711 | Belarus | 30340.65438 | 10613.18594 | 88.01138472 |
| 0.790754768 | Hungary | 29976.1579 | 10579.89127 | 73.0745113 |
| 0.791854408 | Greece | 32408.70414 | 11001.94935 | 69.65274998 |
| 0.792416294 | Serbia | 29928.17205 | 10566.40713 | 73.08219324 |
| 0.795800584 | Montenegro | 29955.13032 | 10573.96097 | 73.04381987 |
| 0.798341027 | Croatia | 29953.92444 | 10594.00092 | 73.0782463 |
| 0.801585034 | Malta | 32376.70619 | 10993.93753 | 69.24511395 |
| 0.803982203 | Guam | 22159.46105 | 8001.531184 | 46.94125922 |
| 0.805020668 | Bahamas | 23748.97976 | 8532.978413 | 51.72893048 |
| 0.805773534 | Italy | 33572.3107 | 11907.29912 | 71.46291747 |
| 0.808536005 | Russian Federation | 31147.26967 | 11200.6375 | 99.72339315 |
| 0.809011652 | Israel | 32400.53996 | 11000.05614 | 69.79939189 |
| 0.810234367 | Brunei Darussalam | 28928.89963 | 10119.3926 | 60.39634331 |
| 0.81061053 | Slovakia | 29962.04577 | 10576.73798 | 73.13158984 |
| 0.812042809 | Poland | 31533.24489 | 11365.28527 | 78.06087652 |
| 0.815143493 | Saudi Arabia | 22434.33042 | 8335.678199 | 63.97565495 |
| 0.821365422 | Bermuda | 23741.32872 | 8530.664342 | 51.86543074 |
| 0.821830853 | United States Virgin Islands | 23738.91975 | 8528.495223 | 51.63355031 |
| 0.825525847 | Puerto Rico | 23740.88226 | 8529.727062 | 51.78265639 |
| 0.826210336 | Greenland | 32837.19243 | 11136.97564 | 66.44476014 |
| 0.828450433 | Czechia | 29944.14824 | 10572.78326 | 72.74849529 |
| 0.830663516 | Latvia | 30338.15207 | 10613.16546 | 87.85979139 |
| 0.835630545 | Cyprus | 32411.95373 | 11000.7739 | 69.90824263 |
| 0.838364875 | France | 31418.47948 | 10893.19806 | 74.92087616 |
| 0.842430731 | Slovenia | 29927.39252 | 10568.36216 | 72.89139271 |
| 0.844252814 | Australia | 26964.15066 | 9688.931866 | 60.3757758 |
| 0.844917787 | Estonia | 30335.96941 | 10612.75604 | 87.75240904 |
| 0.846651055 | Kuwait | 23548.03542 | 8511.294805 | 63.15982121 |
| 0.846860584 | Qatar | 23526.77242 | 8512.825457 | 63.19524469 |
| 0.849317734 | United Arab Emirates | 23538.74516 | 8525.009943 | 63.46291796 |
| 0.849442499 | New Zealand | 28452.74216 | 10345.43006 | 64.04770967 |
| 0.853654016 | Belgium | 32396.92106 | 10998.80615 | 69.4154749 |
| 0.853837004 | Austria | 31759.66748 | 10935.22749 | 71.22133615 |
| 0.856097766 | Singapore | 28481.36284 | 10098.06653 | 59.6775206 |
| 0.856484049 | Lithuania | 31729.84489 | 10764.37457 | 81.03148255 |
| 0.859000182 | United Kingdom | 33487.02013 | 11812.17184 | 72.27253605 |
| 0.859831368 | Finland | 32383.19655 | 10996.08048 | 69.26072351 |
| 0.862448354 | United States of America | 34320.58449 | 12110.67566 | 70.31533991 |
| 0.869444113 | Andorra | 32352.91256 | 10985.72494 | 69.04316358 |
| 0.871241813 | Japan | 29705.70179 | 10726.09348 | 64.56326032 |
| 0.87317068 | Canada | 32911.94038 | 11154.29224 | 67.31211955 |
| 0.87375385 | Ireland | 32406.0159 | 11000.32368 | 69.57996299 |
| 0.874747053 | Taiwan (Province of China) | 17557.80816 | 6542.128082 | 41.12945931 |
| 0.87636168 | Iceland | 32368.66517 | 10991.29033 | 69.37158075 |
| 0.884428955 | Luxembourg | 32279.9639 | 10969.41916 | 74.97635596 |
| 0.886675267 | Republic of Korea | 30412.34572 | 10316.73562 | 62.3898416 |
| 0.886880299 | Sweden | 34297.27358 | 11957.24394 | 68.31134226 |
| 0.888005474 | San Marino | 32430.83813 | 11005.35776 | 70.07442167 |
| 0.888464256 | Netherlands | 34984.91391 | 11114.03531 | 68.15914234 |
| 0.896424204 | Denmark | 33764.78298 | 11052.65667 | 70.3597849 |
| 0.902957091 | Germany | 33107.72532 | 11012.92489 | 72.20522955 |
| 0.908262831 | Monaco | 32411.21203 | 11000.464 | 69.9386071 |
| 0.91613281 | Norway | 35492.43834 | 12225.67197 | 71.25076805 |
| 0.933059111 | Switzerland | 29829.1988 | 10713.80299 | 66.36282415 |

# **Table S5. Net Drift values (percent change per year) for prevalence, incidence, and YLDs of tension-type headache by SDI level, 1990-2021.**

| SDI Level | Prevalence | | | Incidence | | | YLDs | | |
| --- | --- | --- | --- | --- | --- | --- | --- | --- | --- |
|  | Net Drift Percent Per Year | CILo | CIHi | Net Drift Percent Per Year | CILo | CIHi | Net Drift Percent Per Year | CILo | CIHi |
| Global | -0.055528078 | -0.067010226 | -0.044044611 | -0.046986227 | -0.059382594 | -0.034588322 | -0.113792097 | -0.123878452 | -0.103704723 |
| High SDI | -0.07486487 | -0.082173654 | -0.067555551 | -0.069121674 | -0.077603152 | -0.060639476 | -0.073579738 | -0.08968682 | -0.057470058 |
| High-middle SDI | -0.079010161 | -0.104416957 | -0.053596903 | -0.079623983 | -0.104263429 | -0.054978459 | -0.224255062 | -0.249603011 | -0.198900671 |
| Middle SDI | 0.10282004 | 0.076109856 | 0.129537352 | 0.098060426 | 0.071976597 | 0.124151054 | 0.090464877 | 0.065436538 | 0.115499476 |
| Low-middle SDI | 0.005670531 | -0.010906883 | 0.022250694 | -0.002210711 | -0.016183506 | 0.011764036 | 0.033548388 | 0.002450325 | 0.064656122 |
| Low SDI | -0.03924201 | -0.051116522 | -0.027366088 | -0.031452852 | -0.04414077 | -0.018763325 | 0.002475037 | -0.059994796 | 0.064983918 |

# **Table S6. Statistical tests for Age-Period-Cohort effects for prevalence, incidence, and YLDs of tension-type headache by SDI level, 1990-2021.**

| **SDI Level** | **Test** | **Prevalence** | | | **Incidence** | | | **YLDs** | | |
| --- | --- | --- | --- | --- | --- | --- | --- | --- | --- | --- |
|  |  | **X2** | **df** | **P-Value** | **X2** | **df** | **P-Value** | **X2** | **df** | **P-Value** |
| **Global** | NetDrift = 0 | 89.7842198 | 1 | 2.66E-21 | 55.157735 | 1 | 1.11E-13 | 488.348057 | 1 | 3.26E-108 |
|  | THETAa = 0 | 12382.5948 | 1 | 0 | 313.039574 | 1 | 4.75E-70 | 96119.9764 | 1 | 0 |
|  | All Higher-Order Age Deviations = 0 | 391580.647 | 16 | 0 | 141183.09 | 16 | 0 | 404222.351 | 16 | 0 |
|  | All Age Deviations = 0 | 541677.209 | 17 | 0 | 196585.143 | 17 | 0 | 1102231.57 | 17 | 0 |
|  | THETAp = 0 | 48.80699 | 1 | 2.82E-12 | 12.3560175 | 1 | 0.000439567 | 8.58093882 | 1 | 0.003397002 |
|  | All Higher-Order Period Deviations = 0 | 52.7350469 | 3 | 2.09E-11 | 31.3399696 | 3 | 7.21E-07 | 13.6752108 | 3 | 0.003382297 |
|  | All Period Deviations = 0 | 101.11231 | 4 | 5.70E-21 | 43.7554284 | 4 | 7.21E-09 | 22.1908187 | 4 | 0.000183631 |
|  | THETAc = 0 | 31.5785238 | 1 | 1.92E-08 | 21.8260562 | 1 | 2.99E-06 | 61.3165844 | 1 | 4.86E-15 |
|  | All Higher-Order Cohort Deviations = 0 | 611.007557 | 21 | 7.73E-116 | 461.695403 | 21 | 1.44E-84 | 1321.53032 | 21 | 5.97E-267 |
|  | All Cohort Deviations = 0 | 942.634376 | 22 | 3.11E-185 | 675.351123 | 22 | 1.22E-128 | 2771.54899 | 22 | 0 |
|  | All Period RR = 1 | 191.932789 | 5 | 1.51E-39 | 99.6761414 | 5 | 6.18E-20 | 511.312682 | 5 | 2.89E-108 |
|  | All Cohort RR = 1 | 944.923414 | 23 | 6.72E-185 | 675.490309 | 23 | 6.42E-128 | 3491.95558 | 23 | 0 |
|  | All Local Drifts = Net Drift | 779.736525 | 19 | 4.35E-153 | 550.555266 | 19 | 1.33E-104 | 2304.74375 | 19 | 0 |
|  | All Gradient Shifts = CAT | 260.094607 | 6 | 2.85E-53 | 227.066231 | 6 | 3.24E-46 | 984.006925 | 6 | 2.57E-209 |
| **High SDI** | NetDrift = 0 | 402.736467 | 1 | 1.40E-89 | 254.953161 | 1 | 2.16E-57 | 80.0949506 | 1 | 3.57E-19 |
|  | THETAa = 0 | 58162.8347 | 1 | 0 | 3712.12951 | 1 | 0 | 47081.8102 | 1 | 0 |
|  | All Higher-Order Age Deviations = 0 | 369801.031 | 16 | 0 | 144930.461 | 16 | 0 | 52697.415 | 16 | 0 |
|  | All Age Deviations = 0 | 559686.747 | 17 | 0 | 206345.09 | 17 | 0 | 202692.885 | 17 | 0 |
|  | THETAp = 0 | 44.4460113 | 1 | 2.61E-11 | 8.28808519 | 1 | 0.003990605 | 2.9445359 | 1 | 0.086168448 |
|  | All Higher-Order Period Deviations = 0 | 60.2304423 | 3 | 5.25E-13 | 24.8233014 | 3 | 1.68E-05 | 2.15428638 | 3 | 0.541008562 |
|  | All Period Deviations = 0 | 108.609787 | 4 | 1.44E-22 | 34.2003663 | 4 | 6.78E-07 | 5.0103253 | 4 | 0.286239694 |
|  | THETAc = 0 | 3.11049634 | 1 | 0.077789252 | 1.03085221 | 1 | 0.309958591 | 9.95854055 | 1 | 0.001601049 |
|  | All Higher-Order Cohort Deviations = 0 | 751.86682 | 21 | 1.43E-145 | 427.343585 | 21 | 2.00E-77 | 29.7655832 | 21 | 0.096800775 |
|  | All Cohort Deviations = 0 | 756.090642 | 22 | 1.11E-145 | 449.415902 | 22 | 2.44E-81 | 86.3256099 | 22 | 1.43E-09 |
|  | All Period RR = 1 | 511.250657 | 5 | 2.98E-108 | 289.884246 | 5 | 1.50E-60 | 84.8136495 | 5 | 8.24E-17 |
|  | All Cohort RR = 1 | 1648.06878 | 23 | 0 | 926.952684 | 23 | 4.39E-181 | 214.1478 | 23 | 6.01E-33 |
|  | All Local Drifts = Net Drift | 751.513915 | 19 | 4.27E-147 | 446.802739 | 19 | 7.68E-83 | 85.5754546 | 19 | 2.00E-10 |
|  | All Gradient Shifts = CAT | 148.196629 | 6 | 1.86E-29 | 108.282245 | 6 | 4.67E-21 | 68.207466 | 6 | 9.53E-13 |
| **High-middle SDI** | NetDrift = 0 | 37.1128658 | 1 | 1.11E-09 | 40.076056 | 1 | 2.44E-10 | 299.933825 | 1 | 3.41E-67 |
|  | THETAa = 0 | 2503.69822 | 1 | 0 | 86.4318373 | 1 | 1.45E-20 | 16635.639 | 1 | 0 |
|  | All Higher-Order Age Deviations = 0 | 56147.2853 | 16 | 0 | 32060.1413 | 16 | 0 | 40593.1387 | 16 | 0 |
|  | All Age Deviations = 0 | 74960.2564 | 17 | 0 | 40366.0391 | 17 | 0 | 148274.882 | 17 | 0 |
|  | THETAp = 0 | 10.048747 | 1 | 0.001524516 | 1.71565925 | 1 | 0.190252755 | 40.0238954 | 1 | 2.51E-10 |
|  | All Higher-Order Period Deviations = 0 | 60.7028873 | 3 | 4.16E-13 | 41.6333368 | 3 | 4.80E-09 | 9.69023957 | 3 | 0.021391323 |
|  | All Period Deviations = 0 | 69.5761516 | 4 | 2.79E-14 | 43.0233681 | 4 | 1.02E-08 | 51.7273461 | 4 | 1.57E-10 |
|  | THETAc = 0 | 24.170395 | 1 | 8.82E-07 | 20.8568778 | 1 | 4.95E-06 | 36.9872569 | 1 | 1.19E-09 |
|  | All Higher-Order Cohort Deviations = 0 | 357.564563 | 21 | 5.27E-63 | 295.705625 | 21 | 2.38E-50 | 1191.58643 | 21 | 3.68E-239 |
|  | All Cohort Deviations = 0 | 663.772079 | 22 | 3.37E-126 | 554.192127 | 22 | 3.48E-103 | 2152.4082 | 22 | 0 |
|  | All Period RR = 1 | 108.028677 | 5 | 1.07E-21 | 84.042756 | 5 | 1.19E-16 | 350.409568 | 5 | 1.43E-73 |
|  | All Cohort RR = 1 | 663.85822 | 23 | 1.80E-125 | 554.610069 | 23 | 1.45E-102 | 2900.80691 | 23 | 0 |
|  | All Local Drifts = Net Drift | 531.376224 | 19 | 1.44E-100 | 432.670649 | 19 | 6.86E-80 | 1578.08042 | 19 | 9.88131291682493e-324 |
|  | All Gradient Shifts = CAT | 221.858655 | 6 | 4.18E-45 | 240.654852 | 6 | 4.07E-49 | 542.424606 | 6 | 6.06E-114 |
| **Middle SDI** | NetDrift = 0 | 56.9697503 | 1 | 4.43E-14 | 54.33372 | 1 | 1.69E-13 | 50.2217841 | 1 | 1.37E-12 |
|  | THETAa = 0 | 1662.74503 | 1 | 0 | 7.48174395 | 1 | 0.006232767 | 14482.7299 | 1 | 0 |
|  | All Higher-Order Age Deviations = 0 | 115432.758 | 16 | 0 | 50957.761 | 16 | 0 | 122655.829 | 16 | 0 |
|  | All Age Deviations = 0 | 151607.561 | 17 | 0 | 65508.9302 | 17 | 0 | 298200.765 | 17 | 0 |
|  | THETAp = 0 | 0.29975865 | 1 | 0.584033764 | 2.73762792 | 1 | 0.098010156 | 25.0479281 | 1 | 5.59E-07 |
|  | All Higher-Order Period Deviations = 0 | 53.0491189 | 3 | 1.79E-11 | 34.7389787 | 3 | 1.38E-07 | 38.8786465 | 3 | 1.84E-08 |
|  | All Period Deviations = 0 | 53.1860774 | 4 | 7.79E-11 | 37.8448597 | 4 | 1.21E-07 | 63.024063 | 4 | 6.71E-13 |
|  | THETAc = 0 | 34.3633285 | 1 | 4.57E-09 | 27.9989835 | 1 | 1.21E-07 | 13.0739746 | 1 | 0.000299427 |
|  | All Higher-Order Cohort Deviations = 0 | 575.870786 | 21 | 1.88E-108 | 475.28516 | 21 | 2.13E-87 | 460.393483 | 21 | 2.70E-84 |
|  | All Cohort Deviations = 0 | 689.679867 | 22 | 1.17E-131 | 570.342698 | 22 | 1.44E-106 | 643.181664 | 22 | 7.27E-122 |
|  | All Period RR = 1 | 109.752769 | 5 | 4.62E-22 | 91.9960138 | 5 | 2.56E-18 | 113.234895 | 5 | 8.48E-23 |
|  | All Cohort RR = 1 | 1879.23099 | 23 | 0 | 1651.45425 | 23 | 0 | 1794.87474 | 23 | 0 |
|  | All Local Drifts = Net Drift | 560.007707 | 19 | 1.36E-106 | 469.495849 | 19 | 1.38E-87 | 508.008171 | 19 | 1.17E-95 |
|  | All Gradient Shifts = CAT | 91.2806691 | 6 | 1.64E-17 | 116.069049 | 6 | 1.09E-22 | 82.9768085 | 6 | 8.66E-16 |
| **Low-middle SDI** | NetDrift = 0 | 0.4494472 | 1 | 0.502597587 | 0.0961478 | 1 | 0.756502335 | 4.47094588 | 1 | 0.034475901 |
|  | THETAa = 0 | 3709.76223 | 1 | 0 | 1.24680073 | 1 | 0.264164401 | 8146.4885 | 1 | 0 |
|  | All Higher-Order Age Deviations = 0 | 425680.056 | 16 | 0 | 206777.407 | 16 | 0 | 117637.139 | 16 | 0 |
|  | All Age Deviations = 0 | 569510.332 | 17 | 0 | 286277.198 | 17 | 0 | 242351.313 | 17 | 0 |
|  | THETAp = 0 | 202.292119 | 1 | 6.60E-46 | 138.459836 | 1 | 5.78E-32 | 103.251156 | 1 | 2.95E-24 |
|  | All Higher-Order Period Deviations = 0 | 15.6408246 | 3 | 0.001343383 | 12.9358227 | 3 | 0.004777468 | 4.46848839 | 3 | 0.215118332 |
|  | All Period Deviations = 0 | 223.927663 | 4 | 2.68E-47 | 157.183433 | 4 | 5.87E-33 | 113.64572 | 4 | 1.21E-23 |
|  | THETAc = 0 | 0.72254824 | 1 | 0.395309309 | 0.69659243 | 1 | 0.403931061 | 0.22273421 | 1 | 0.636964442 |
|  | All Higher-Order Cohort Deviations = 0 | 44.6571668 | 21 | 0.001920542 | 51.0737892 | 21 | 0.000258128 | 9.37631547 | 21 | 0.985930603 |
|  | All Cohort Deviations = 0 | 75.0528723 | 22 | 1.04E-07 | 73.0364598 | 22 | 2.18E-07 | 9.47431323 | 22 | 0.990477449 |
|  | All Period RR = 1 | 224.280162 | 5 | 1.80E-46 | 157.367218 | 5 | 3.60E-32 | 118.140722 | 5 | 7.77E-24 |
|  | All Cohort RR = 1 | 79.208909 | 23 | 4.26E-08 | 94.0257411 | 23 | 1.49E-10 | 33.0182523 | 23 | 0.080749104 |
|  | All Local Drifts = Net Drift | 68.5201388 | 19 | 1.62E-07 | 63.9419024 | 19 | 9.06E-07 | 7.64552842 | 19 | 0.98989581 |
|  | All Gradient Shifts = CAT | 29.1853849 | 6 | 5.61E-05 | 15.0349716 | 6 | 0.019986452 | 1.53101409 | 6 | 0.957407844 |
| **Low SDI** | NetDrift = 0 | 41.9334983 | 1 | 9.44E-11 | 23.5971641 | 1 | 1.19E-06 | 0.00602663 | 1 | 0.938121308 |
|  | THETAa = 0 | 6581.29102 | 1 | 0 | 23.5304934 | 1 | 1.23E-06 | 1834.12615 | 1 | 0 |
|  | All Higher-Order Age Deviations = 0 | 1562762.14 | 16 | 0 | 537631.209 | 16 | 0 | 63690.6153 | 16 | 0 |
|  | All Age Deviations = 0 | 2292034.67 | 17 | 0 | 846446.331 | 17 | 0 | 128961.717 | 17 | 0 |
|  | THETAp = 0 | 451.51927 | 1 | 3.37E-100 | 269.712909 | 1 | 1.31E-60 | 26.333543 | 1 | 2.87E-07 |
|  | All Higher-Order Period Deviations = 0 | 26.8787206 | 3 | 6.24E-06 | 21.1163026 | 3 | 9.96E-05 | 2.34404594 | 3 | 0.504135427 |
|  | All Period Deviations = 0 | 520.661224 | 4 | 2.28E-111 | 318.85656 | 4 | 9.26E-68 | 30.6943303 | 4 | 3.53E-06 |
|  | THETAc = 0 | 1.62017399 | 1 | 0.203067529 | 2.70761931 | 1 | 0.099869932 | 0.016027 | 1 | 0.899258744 |
|  | All Higher-Order Cohort Deviations = 0 | 90.6631402 | 21 | 1.24E-10 | 94.9489575 | 21 | 2.23E-11 | 5.08938112 | 21 | 0.99984705 |
|  | All Cohort Deviations = 0 | 96.059136 | 22 | 3.13E-11 | 106.337909 | 22 | 4.94E-13 | 5.26754915 | 22 | 0.999903046 |
|  | All Period RR = 1 | 564.127838 | 5 | 1.14E-119 | 343.942678 | 5 | 3.52E-72 | 30.6996648 | 5 | 1.07E-05 |
|  | All Cohort RR = 1 | 715.582149 | 23 | 2.31E-136 | 617.23043 | 23 | 1.12E-115 | 5.2781878 | 23 | 0.999953738 |
|  | All Local Drifts = Net Drift | 93.6766176 | 19 | 7.35E-12 | 100.87994 | 19 | 3.71E-13 | 4.98955901 | 19 | 0.999439575 |
|  | All Gradient Shifts = CAT | 17.8622598 | 6 | 0.006585793 | 20.9934334 | 6 | 0.001839606 | 0.49720715 | 6 | 0.997872323 |

# **Table S7. Estimated parameters from Age-Period-Cohort analysis for prevalence, incidence, and YLDs of tension-type headache by SDI level, 1990-2021.**

| Table S7: Estimated parameters from Age-Period-Cohort analysis for prevalence, incidence, and YLDs of tension-type headache by SDI level, 1990-2021. | | | | | | | | | | | |
| --- | --- | --- | --- | --- | --- | --- | --- | --- | --- | --- | --- |
| sdi level | result type | x value | Prevalence | | | Incidence | | | YLDs | | |
|  |  |  | y value | CILo | CIHi | y value | CILo | CIHi | y value | CILo | CIHi |
| Global | longitudinal age curve | 7.5 | 7132.9268 | 7095.4998 | 7170.5513 | 6236.8124 | 6206.8851 | 6266.884 | 8.1936162 | 8.1536311 | 8.2337974 |
|  |  | 12.5 | 24385.688 | 24285.69 | 24486.097 | 10286.736 | 10241.633 | 10332.038 | 31.774747 | 31.669869 | 31.879973 |
|  |  | 17.5 | 28776.591 | 28664.494 | 28889.126 | 10428.109 | 10383.732 | 10472.674 | 51.950437 | 51.798574 | 52.102745 |
|  |  | 22.5 | 30770.36 | 30654.804 | 30886.353 | 10899.442 | 10854.466 | 10944.604 | 62.883411 | 62.711208 | 63.056087 |
|  |  | 27.5 | 32405.637 | 32288.205 | 32523.496 | 11796.368 | 11749.434 | 11843.488 | 65.909732 | 65.737127 | 66.08279 |
|  |  | 32.5 | 34303.87 | 34184.035 | 34424.126 | 11495.037 | 11450.516 | 11539.73 | 74.818183 | 74.63235 | 75.00448 |
|  |  | 37.5 | 33293.702 | 33180.206 | 33407.587 | 10964.07 | 10922.524 | 11005.775 | 83.534897 | 83.337118 | 83.733144 |
|  |  | 42.5 | 32717.685 | 32615.912 | 32819.775 | 10737.328 | 10700.168 | 10774.617 | 89.118445 | 88.92838 | 89.308916 |
|  |  | 47.5 | 30359.572 | 30264.952 | 30454.487 | 9462.8761 | 9429.7602 | 9496.1082 | 90.377726 | 90.188341 | 90.567508 |
|  |  | 52.5 | 28162.04 | 28071.866 | 28252.505 | 9410.9509 | 9377.7012 | 9444.3185 | 88.428351 | 88.240652 | 88.616449 |
|  |  | 57.5 | 28048.665 | 27955.345 | 28142.297 | 8514.1782 | 8482.1009 | 8546.3767 | 85.974049 | 85.783784 | 86.164736 |
|  |  | 62.5 | 25066.711 | 24974.449 | 25159.314 | 8207.5507 | 8174.1099 | 8241.1282 | 83.413125 | 83.214431 | 83.612293 |
|  |  | 67.5 | 27740.724 | 27630.701 | 27851.185 | 10253.818 | 10210.431 | 10297.389 | 79.74329 | 79.529406 | 79.957749 |
|  |  | 72.5 | 29631.288 | 29488.56 | 29774.707 | 9531.5466 | 9480.592 | 9582.7751 | 68.089742 | 67.854111 | 68.326191 |
|  |  | 77.5 | 26320.221 | 26166.87 | 26474.471 | 8168.9539 | 8115.5562 | 8222.703 | 56.530027 | 56.285357 | 56.775761 |
|  |  | 82.5 | 25260.819 | 25077.882 | 25445.09 | 8402.0255 | 8335.0157 | 8469.5741 | 50.856309 | 50.572036 | 51.142181 |
|  |  | 87.5 | 24505.136 | 24263.361 | 24749.32 | 7763.7285 | 7677.9718 | 7850.4429 | 47.268748 | 46.894476 | 47.646008 |
|  |  | 92.5 | 24115.853 | 23742.156 | 24495.432 | 9015.001 | 8868.1448 | 9164.2891 | 38.491592 | 37.962341 | 39.028221 |
|  |  | 97.5 | 31562.097 | 30777.364 | 32366.839 | 11914.066 | 11605.406 | 12230.934 | 34.215947 | 33.306209 | 35.150533 |
|  | period rate ratio | 1994.5 | 1.0092366 | 1.0070605 | 1.0114174 | 1.0059658 | 1.003668 | 1.0082689 | 1.0126864 | 1.0109315 | 1.0144444 |
|  |  | 1999.5 | 0.9987583 | 0.9967718 | 1.0007487 | 0.9985303 | 0.9964352 | 1.0006297 | 1.0042549 | 1.002693 | 1.0058192 |
|  |  | 2004.5 | 1 | 1 | 1 | 1 | 1 | 1 | 1 | 1 | 1 |
|  |  | 2009.5 | 0.9963372 | 0.9944169 | 0.9982612 | 0.9964076 | 0.9943753 | 0.998444 | 0.9942895 | 0.9927937 | 0.9957876 |
|  |  | 2014.5 | 0.9915843 | 0.9895044 | 0.9936685 | 0.9922004 | 0.9899857 | 0.9944201 | 0.9874902 | 0.9858262 | 0.989157 |
|  |  | 2019.5 | 0.9948266 | 0.9924572 | 0.9972017 | 0.9940522 | 0.9915159 | 0.9965949 | 0.9841301 | 0.9821907 | 0.9860733 |
|  | cohort rate ratio | 1897 | 1.0580346 | 0.9695444 | 1.1546013 | 1.0470119 | 0.9561814 | 1.1464706 | 1.0916104 | 0.9922271 | 1.200948 |
|  |  | 1902 | 1.0591704 | 1.0167724 | 1.1033363 | 1.0496125 | 1.0058359 | 1.0952944 | 1.0939498 | 1.0526958 | 1.1368206 |
|  |  | 1907 | 1.0552316 | 1.0322434 | 1.0787318 | 1.047692 | 1.022664 | 1.0733326 | 1.0882822 | 1.0680634 | 1.1088838 |
|  |  | 1912 | 1.0519861 | 1.0373096 | 1.0668703 | 1.04573 | 1.0296843 | 1.0620257 | 1.0801501 | 1.0678473 | 1.0925947 |
|  |  | 1917 | 1.0456324 | 1.0349595 | 1.0564153 | 1.0396207 | 1.0277765 | 1.0516013 | 1.0586731 | 1.0501238 | 1.067292 |
|  |  | 1922 | 1.042802 | 1.034872 | 1.0507927 | 1.0372111 | 1.0284145 | 1.046083 | 1.0566351 | 1.0505174 | 1.0627885 |
|  |  | 1927 | 1.0381214 | 1.0317855 | 1.0444963 | 1.0331023 | 1.0262122 | 1.0400386 | 1.0581641 | 1.0535248 | 1.062824 |
|  |  | 1932 | 1.0272778 | 1.0218856 | 1.0326985 | 1.0267467 | 1.020842 | 1.0326855 | 1.0406262 | 1.0368702 | 1.0443957 |
|  |  | 1937 | 1.0199294 | 1.015258 | 1.0246222 | 1.0198695 | 1.0147026 | 1.0250628 | 1.0362208 | 1.0330183 | 1.0394332 |
|  |  | 1942 | 1.0163468 | 1.0121554 | 1.0205556 | 1.0147978 | 1.0101737 | 1.0194431 | 1.01612 | 1.0133126 | 1.0189351 |
|  |  | 1947 | 1.0113855 | 1.0076667 | 1.015118 | 1.0108888 | 1.0067683 | 1.0150261 | 1.0054567 | 1.0029862 | 1.0079332 |
|  |  | 1952 | 1 | 1 | 1 | 1 | 1 | 1 | 1 | 1 | 1 |
|  |  | 1957 | 1.0058823 | 1.0025887 | 1.0091867 | 1.0045254 | 1.0008578 | 1.0082065 | 0.9988421 | 0.9966453 | 1.0010438 |
|  |  | 1962 | 1.0004741 | 0.9972099 | 1.003749 | 1.0005898 | 0.9969484 | 1.0042446 | 0.985487 | 0.9832997 | 0.9876792 |
|  |  | 1967 | 0.9826043 | 0.9793421 | 0.9858773 | 0.9849598 | 0.9813285 | 0.9886045 | 0.9638355 | 0.9616388 | 0.9660371 |
|  |  | 1972 | 0.9874252 | 0.9840472 | 0.9908147 | 0.989749 | 0.985988 | 0.9935244 | 0.9677265 | 0.9654228 | 0.9700358 |
|  |  | 1977 | 1.0014222 | 0.9978664 | 1.0049907 | 1.0021461 | 0.9982103 | 1.0060974 | 0.980182 | 0.977717 | 0.9826533 |
|  |  | 1982 | 1.0065887 | 1.0028797 | 1.0103114 | 1.0067897 | 1.0027043 | 1.0108917 | 0.9852906 | 0.9826691 | 0.9879191 |
|  |  | 1987 | 0.999227 | 0.9954214 | 1.003047 | 0.9990448 | 0.9948846 | 1.0032224 | 0.9783941 | 0.9756489 | 0.9811471 |
|  |  | 1992 | 1.0062423 | 1.00221 | 1.0102908 | 1.0062625 | 1.0018977 | 1.0106464 | 0.9808833 | 0.9779138 | 0.9838619 |
|  |  | 1997 | 1.0131731 | 1.0088359 | 1.017529 | 1.0138604 | 1.0092224 | 1.0185197 | 0.9864242 | 0.9831294 | 0.9897301 |
|  |  | 2002 | 1.0119713 | 1.0072142 | 1.0167508 | 1.0131931 | 1.0082358 | 1.0181748 | 0.9906528 | 0.9868099 | 0.9945107 |
|  |  | 2007 | 1.0031299 | 0.9975544 | 1.0087367 | 1.006404 | 1.0009339 | 1.0119041 | 0.9882143 | 0.9831899 | 0.9932643 |
|  |  | 2012 | 0.9928612 | 0.9835374 | 1.0022735 | 0.9953961 | 0.9882377 | 1.0026064 | 0.9819345 | 0.9723815 | 0.9915814 |
|  | local drifts | 7.5 | -0.024246 | -0.051645 | 0.0031607 | -0.010588 | -0.030621 | 0.0094482 | 0.0255323 | -0.003649 | 0.0547226 |
|  |  | 12.5 | 0.0158154 | 0.0005272 | 0.031106 | 0.0273146 | 0.0132337 | 0.0413975 | 0.0330352 | 0.0179216 | 0.0481512 |
|  |  | 17.5 | 0.0451251 | 0.0326095 | 0.0576423 | 0.0474451 | 0.034921 | 0.0599707 | 0.0337884 | 0.0225126 | 0.0450654 |
|  |  | 22.5 | 0.0776147 | 0.0661174 | 0.0891133 | 0.0714088 | 0.0594277 | 0.0833913 | 0.0519028 | 0.0421697 | 0.0616369 |
|  |  | 27.5 | 0.091269 | 0.0801023 | 0.102437 | 0.0798348 | 0.0679579 | 0.091713 | 0.0718842 | 0.0628268 | 0.0809423 |
|  |  | 32.5 | 0.0458314 | 0.0345441 | 0.05712 | 0.0402852 | 0.0280401 | 0.0525318 | 0.0244145 | 0.0156518 | 0.0331779 |
|  |  | 37.5 | 0.0064264 | -0.005426 | 0.0182799 | 0.0118696 | -0.001171 | 0.0249125 | -0.045969 | -0.054733 | -0.037205 |
|  |  | 42.5 | -0.037979 | -0.050665 | -0.025291 | -0.028272 | -0.042349 | -0.014193 | -0.124061 | -0.133029 | -0.115093 |
|  |  | 47.5 | -0.101615 | -0.115565 | -0.087662 | -0.088565 | -0.10415 | -0.072978 | -0.179954 | -0.189481 | -0.170426 |
|  |  | 52.5 | -0.111648 | -0.127192 | -0.096102 | -0.100193 | -0.117524 | -0.082859 | -0.185812 | -0.196218 | -0.175404 |
|  |  | 57.5 | -0.079207 | -0.096341 | -0.06207 | -0.078128 | -0.097216 | -0.059036 | -0.175782 | -0.187193 | -0.164371 |
|  |  | 62.5 | -0.096713 | -0.11577 | -0.077654 | -0.098399 | -0.119391 | -0.077404 | -0.183943 | -0.196789 | -0.171095 |
|  |  | 67.5 | -0.13554 | -0.156924 | -0.114152 | -0.122503 | -0.145855 | -0.099147 | -0.231394 | -0.246464 | -0.216322 |
|  |  | 72.5 | -0.127761 | -0.153255 | -0.10226 | -0.107873 | -0.136189 | -0.079549 | -0.21355 | -0.232748 | -0.194349 |
|  |  | 77.5 | -0.125105 | -0.158386 | -0.091812 | -0.101426 | -0.138689 | -0.06415 | -0.16008 | -0.186358 | -0.133796 |
|  |  | 82.5 | -0.121275 | -0.166452 | -0.076078 | -0.095128 | -0.145155 | -0.045076 | -0.147168 | -0.184006 | -0.110317 |
|  |  | 87.5 | -0.10095 | -0.170152 | -0.031699 | -0.079819 | -0.155946 | -0.003634 | -0.164156 | -0.222828 | -0.10545 |
|  |  | 92.5 | -0.081093 | -0.20623 | 0.0441998 | -0.065862 | -0.197276 | 0.0657256 | -0.15697 | -0.273383 | -0.040422 |
|  |  | 97.5 | -0.065225 | -0.327671 | 0.1979115 | -0.04433 | -0.317157 | 0.2292451 | -0.153401 | -0.436347 | 0.1303482 |
| High SDI | longitudinal age curve | 7.5 | 8395.1859 | 8350.9131 | 8439.6934 | 7324.1379 | 7289.3517 | 7359.0901 | 9.6608914 | 9.5319873 | 9.7915386 |
|  |  | 12.5 | 29970.031 | 29860.642 | 30079.82 | 13134.924 | 13081.433 | 13188.633 | 38.329391 | 38.017722 | 38.643615 |
|  |  | 17.5 | 37923.912 | 37798.137 | 38050.106 | 13754.989 | 13702.063 | 13808.119 | 62.665535 | 62.238157 | 63.095848 |
|  |  | 22.5 | 40131.35 | 40006.46 | 40256.629 | 13845.828 | 13795.214 | 13896.628 | 73.471766 | 73.017121 | 73.929241 |
|  |  | 27.5 | 42078.199 | 41954.786 | 42201.974 | 14981.111 | 14929.527 | 15032.872 | 77.399497 | 76.953449 | 77.848131 |
|  |  | 32.5 | 44855.392 | 44731.662 | 44979.463 | 14533.992 | 14486.222 | 14581.918 | 91.423998 | 90.941007 | 91.909554 |
|  |  | 37.5 | 43491.469 | 43376.551 | 43606.691 | 13641.19 | 13597.981 | 13684.535 | 105.73522 | 105.21751 | 106.25548 |
|  |  | 42.5 | 41953.825 | 41854.653 | 42053.232 | 13178.709 | 13141.359 | 13216.165 | 112.90223 | 112.41633 | 113.39023 |
|  |  | 47.5 | 39077.054 | 38985.874 | 39168.448 | 11898.24 | 11864.715 | 11931.859 | 112.37416 | 111.9044 | 112.8459 |
|  |  | 52.5 | 37489.557 | 37401.476 | 37577.845 | 12389.687 | 12355.291 | 12424.18 | 106.84099 | 106.39086 | 107.29303 |
|  |  | 57.5 | 37875.707 | 37784.973 | 37966.66 | 11105.268 | 11072.804 | 11137.827 | 101.4531 | 101.01005 | 101.89809 |
|  |  | 62.5 | 32421.597 | 32335.859 | 32507.562 | 9693.9998 | 9662.8562 | 9725.2437 | 95.351563 | 94.904191 | 95.801044 |
|  |  | 67.5 | 31743.831 | 31651.778 | 31836.151 | 10762.361 | 10725.858 | 10798.988 | 87.830488 | 87.369739 | 88.293667 |
|  |  | 72.5 | 31371.403 | 31261.879 | 31481.311 | 9740.5407 | 9699.7493 | 9781.5036 | 72.693009 | 72.211428 | 73.177801 |
|  |  | 77.5 | 27830.935 | 27716.338 | 27946.006 | 8663.7772 | 8621.0341 | 8706.7322 | 60.003119 | 59.520943 | 60.489201 |
|  |  | 82.5 | 27542.224 | 27408.503 | 27676.598 | 9216.9009 | 9164.3669 | 9269.736 | 54.846907 | 54.309726 | 55.389402 |
|  |  | 87.5 | 27026.114 | 26860.062 | 27193.192 | 8405.3502 | 8343.6222 | 8467.5348 | 51.863295 | 51.201813 | 52.533324 |
|  |  | 92.5 | 25789.195 | 25557.626 | 26022.863 | 9271.5491 | 9177.1994 | 9366.8687 | 42.056162 | 41.204007 | 42.925941 |
|  |  | 97.5 | 32442.731 | 31998.166 | 32893.472 | 11947.818 | 11765.268 | 12133.201 | 36.120271 | 34.787199 | 37.504427 |
|  | period rate ratio | 1994.5 | 1.0093715 | 1.0076559 | 1.0110901 | 1.0066235 | 1.0046579 | 1.0085929 | 1.0051965 | 1.0017182 | 1.0086868 |
|  |  | 1999.5 | 1.0084409 | 1.0067805 | 1.0101041 | 1.0051935 | 1.0032914 | 1.0070992 | 1.0044554 | 1.0011386 | 1.0077833 |
|  |  | 2004.5 | 1 | 1 | 1 | 1 | 1 | 1 | 1 | 1 | 1 |
|  |  | 2009.5 | 0.9942963 | 0.9926847 | 0.9959104 | 0.9942631 | 0.9924083 | 0.9961213 | 0.9957145 | 0.9924919 | 0.9989475 |
|  |  | 2014.5 | 0.9929438 | 0.9912798 | 0.9946107 | 0.9916008 | 0.9896836 | 0.9935216 | 0.9929745 | 0.9896013 | 0.9963591 |
|  |  | 2019.5 | 0.9935721 | 0.9918004 | 0.9953471 | 0.9917544 | 0.9897053 | 0.9938078 | 0.9872589 | 0.9836217 | 0.9909096 |
|  | cohort rate ratio | 1897 | 1.0322711 | 0.9863012 | 1.0803835 | 1.026602 | 0.9760461 | 1.0797766 | 1.0663798 | 0.9384976 | 1.2116875 |
|  |  | 1902 | 1.0275171 | 1.0049938 | 1.0505451 | 1.0243453 | 0.9992571 | 1.0500634 | 1.0627753 | 1.0071865 | 1.1214322 |
|  |  | 1907 | 1.0232812 | 1.0105195 | 1.036204 | 1.0205757 | 1.0056904 | 1.0356813 | 1.0555375 | 1.0265928 | 1.0852984 |
|  |  | 1912 | 1.0203828 | 1.0117665 | 1.0290724 | 1.0198552 | 1.0098045 | 1.0300058 | 1.0459676 | 1.0272398 | 1.0650369 |
|  |  | 1917 | 1.0210765 | 1.0143271 | 1.0278709 | 1.0209185 | 1.0129429 | 1.0289568 | 1.0340102 | 1.0199359 | 1.0482787 |
|  |  | 1922 | 1.0179876 | 1.0127663 | 1.0232358 | 1.0175168 | 1.0113037 | 1.0237681 | 1.0344351 | 1.0239006 | 1.0450781 |
|  |  | 1927 | 1.0111619 | 1.0068277 | 1.0155149 | 1.0109388 | 1.0058177 | 1.0160861 | 1.0259693 | 1.0176088 | 1.0343986 |
|  |  | 1932 | 1.00893 | 1.00515 | 1.0127243 | 1.0075276 | 1.0030228 | 1.0120526 | 1.0193043 | 1.0122516 | 1.0264062 |
|  |  | 1937 | 1.0112279 | 1.0079053 | 1.0145616 | 1.0081829 | 1.0041976 | 1.012184 | 1.0167054 | 1.0105825 | 1.0228655 |
|  |  | 1942 | 1.0149902 | 1.0120003 | 1.0179888 | 1.0119609 | 1.0083926 | 1.0155419 | 1.0130738 | 1.0076526 | 1.0185241 |
|  |  | 1947 | 1.0115231 | 1.0088644 | 1.0141888 | 1.0106592 | 1.0074738 | 1.0138548 | 1.0078509 | 1.0030734 | 1.0126512 |
|  |  | 1952 | 1 | 1 | 1 | 1 | 1 | 1 | 1 | 1 | 1 |
|  |  | 1957 | 0.9963797 | 0.9939647 | 0.9988006 | 0.9947752 | 0.9918733 | 0.9976855 | 0.9942411 | 0.9898846 | 0.9986169 |
|  |  | 1962 | 0.9946272 | 0.9921832 | 0.9970772 | 0.9926819 | 0.9897533 | 0.9956192 | 0.9923603 | 0.9879124 | 0.9968282 |
|  |  | 1967 | 0.9886517 | 0.9861132 | 0.9911967 | 0.9868896 | 0.9838645 | 0.9899241 | 0.9892114 | 0.9845534 | 0.9938914 |
|  |  | 1972 | 0.979929 | 0.977287 | 0.9825781 | 0.9810232 | 0.9778631 | 0.9841934 | 0.9849477 | 0.980017 | 0.9899032 |
|  |  | 1977 | 0.9698172 | 0.9670524 | 0.9725898 | 0.9722109 | 0.9689185 | 0.9755145 | 0.9825529 | 0.9772431 | 0.9878916 |
|  |  | 1982 | 0.9615738 | 0.958683 | 0.9644733 | 0.9649059 | 0.9614837 | 0.9683403 | 0.9799374 | 0.9742073 | 0.9857012 |
|  |  | 1987 | 0.9556401 | 0.952592 | 0.958698 | 0.9600646 | 0.9564952 | 0.9636473 | 0.9774707 | 0.9712225 | 0.983759 |
|  |  | 1992 | 0.9628594 | 0.9595332 | 0.9661971 | 0.9654166 | 0.9615896 | 0.9692588 | 0.9821547 | 0.9751396 | 0.9892204 |
|  |  | 1997 | 0.967736 | 0.9640197 | 0.9714667 | 0.9685251 | 0.9643359 | 0.9727325 | 0.9869792 | 0.978869 | 0.9951567 |
|  |  | 2002 | 0.9720866 | 0.9677629 | 0.9764296 | 0.9736317 | 0.9689403 | 0.9783459 | 0.9936799 | 0.9836781 | 1.0037833 |
|  |  | 2007 | 0.9661289 | 0.9606284 | 0.9716608 | 0.9714426 | 0.965936 | 0.9769805 | 0.9940104 | 0.9799827 | 1.0082388 |
|  |  | 2012 | 0.9559004 | 0.9455471 | 0.966367 | 0.9600497 | 0.9520582 | 0.9681084 | 0.9873511 | 0.9586151 | 1.0169485 |
|  | local drifts | 7.5 | 0.0091528 | -0.02332 | 0.0416365 | 0.0136287 | -0.011127 | 0.0383904 | 0.0531853 | -0.035622 | 0.1420715 |
|  |  | 12.5 | 0.0456516 | 0.0286094 | 0.0626966 | 0.0451932 | 0.0287683 | 0.0616209 | 0.0717602 | 0.0279281 | 0.1156116 |
|  |  | 17.5 | 0.0219319 | 0.00881 | 0.0350556 | 0.0137682 | -8.15E-05 | 0.0276198 | 0.0471917 | 0.0159174 | 0.0784758 |
|  |  | 22.5 | -0.051641 | -0.062996 | -0.040283 | -0.051517 | -0.064081 | -0.038951 | 0.0037521 | -0.021968 | 0.0294791 |
|  |  | 27.5 | -0.123356 | -0.133784 | -0.112927 | -0.104129 | -0.115962 | -0.092295 | -0.035035 | -0.057741 | -0.012324 |
|  |  | 32.5 | -0.167641 | -0.177614 | -0.157666 | -0.139134 | -0.150725 | -0.127542 | -0.060714 | -0.081427 | -0.039997 |
|  |  | 37.5 | -0.149847 | -0.159795 | -0.139898 | -0.126152 | -0.137916 | -0.114387 | -0.060879 | -0.080444 | -0.041311 |
|  |  | 42.5 | -0.119477 | -0.129682 | -0.109271 | -0.107672 | -0.119843 | -0.095499 | -0.068181 | -0.087374 | -0.048984 |
|  |  | 47.5 | -0.111174 | -0.12183 | -0.100516 | -0.108802 | -0.121582 | -0.096021 | -0.085318 | -0.104834 | -0.065798 |
|  |  | 52.5 | -0.106013 | -0.117448 | -0.094576 | -0.105383 | -0.119099 | -0.091665 | -0.097909 | -0.118698 | -0.077116 |
|  |  | 57.5 | -0.085528 | -0.097887 | -0.073167 | -0.07966 | -0.094579 | -0.06474 | -0.105828 | -0.128353 | -0.083297 |
|  |  | 62.5 | -0.056843 | -0.07049 | -0.043195 | -0.051087 | -0.06748 | -0.034692 | -0.102433 | -0.127408 | -0.077452 |
|  |  | 67.5 | -0.025189 | -0.040364 | -0.010012 | -0.023624 | -0.041632 | -0.005613 | -0.094623 | -0.123121 | -0.066116 |
|  |  | 72.5 | -0.010423 | -0.02792 | 0.0070776 | -0.017216 | -0.038029 | 0.0036021 | -0.097481 | -0.131951 | -0.063 |
|  |  | 77.5 | -0.029761 | -0.051606 | -0.007911 | -0.0429 | -0.068787 | -0.017005 | -0.091763 | -0.136758 | -0.046748 |
|  |  | 82.5 | -0.050097 | -0.077759 | -0.022426 | -0.059211 | -0.091623 | -0.02679 | -0.110262 | -0.169206 | -0.051284 |
|  |  | 87.5 | -0.05763 | -0.097323 | -0.017922 | -0.05371 | -0.100163 | -0.007236 | -0.13257 | -0.220307 | -0.044756 |
|  |  | 92.5 | -0.054331 | -0.1225 | 0.0138835 | -0.042182 | -0.11889 | 0.0345841 | -0.141791 | -0.30537 | 0.022056 |
|  |  | 97.5 | -0.052196 | -0.189627 | 0.085423 | -0.031541 | -0.18402 | 0.1211709 | -0.139043 | -0.518833 | 0.242196 |
| High-middle SDI | longitudinal age curve | 7.5 | 6514.8462 | 6425.0543 | 6605.893 | 5758.7374 | 5696.629 | 5821.523 | 8.0869926 | 7.9637965 | 8.2120944 |
|  |  | 12.5 | 22974.781 | 22749.977 | 23201.807 | 10021.391 | 9927.542 | 10116.127 | 32.701861 | 32.404064 | 33.002396 |
|  |  | 17.5 | 27517.376 | 27268.479 | 27768.545 | 9893.4449 | 9804.9435 | 9982.745 | 55.155963 | 54.735953 | 55.579197 |
|  |  | 22.5 | 28389.535 | 28145.327 | 28635.863 | 10134.783 | 10048.134 | 10222.18 | 66.918812 | 66.454587 | 67.38628 |
|  |  | 27.5 | 30505.057 | 30255.744 | 30756.425 | 11417.217 | 11324.751 | 11510.438 | 71.371695 | 70.907396 | 71.839034 |
|  |  | 32.5 | 33135.146 | 32877.632 | 33394.677 | 11330.61 | 11242.298 | 11419.616 | 82.287954 | 81.78967 | 82.789274 |
|  |  | 37.5 | 32245.797 | 32003.141 | 32490.294 | 10599.607 | 10519.193 | 10680.637 | 92.717952 | 92.190295 | 93.248628 |
|  |  | 42.5 | 30958.639 | 30747.321 | 31171.41 | 10112.708 | 10043.008 | 10182.891 | 98.697589 | 98.197221 | 99.200508 |
|  |  | 47.5 | 28367.458 | 28173.7 | 28562.549 | 8897.483 | 8835.6815 | 8959.7167 | 99.981214 | 99.486784 | 100.4781 |
|  |  | 52.5 | 26352.68 | 26169.118 | 26537.528 | 8884.137 | 8822.3633 | 8946.3432 | 99.704245 | 99.211158 | 100.19978 |
|  |  | 57.5 | 26309.021 | 26119.966 | 26499.444 | 8087.0778 | 8027.6239 | 8146.972 | 99.085827 | 98.582182 | 99.592046 |
|  |  | 62.5 | 23774.752 | 23587.031 | 23963.966 | 7921.371 | 7858.9436 | 7984.2942 | 96.478206 | 95.953229 | 97.006054 |
|  |  | 67.5 | 26635.182 | 26410.728 | 26861.544 | 9990.0501 | 9908.9569 | 10071.807 | 87.582205 | 87.040315 | 88.127468 |
|  |  | 72.5 | 28529.553 | 28240.256 | 28821.814 | 9269.1601 | 9174.8909 | 9364.398 | 71.5417 | 70.971015 | 72.116974 |
|  |  | 77.5 | 25235.943 | 24931.093 | 25544.521 | 7882.3673 | 7785.7118 | 7980.2228 | 58.295695 | 57.719416 | 58.877727 |
|  |  | 82.5 | 24030.089 | 23673.267 | 24392.288 | 8002.0537 | 7883.3324 | 8122.5629 | 51.806064 | 51.148593 | 52.471986 |
|  |  | 87.5 | 22898.441 | 22429.527 | 23377.157 | 7269.8101 | 7118.4843 | 7424.3528 | 47.387966 | 46.521517 | 48.270552 |
|  |  | 92.5 | 22365.289 | 21620.436 | 23135.803 | 8564.9265 | 8294.9963 | 8843.6405 | 37.870767 | 36.617369 | 39.167069 |
|  |  | 97.5 | 30155.697 | 28433.769 | 31981.904 | 11647.293 | 11023.566 | 12306.312 | 33.525085 | 31.199748 | 36.02373 |
|  | period rate ratio | 1994.5 | 1.0128428 | 1.0078697 | 1.0178405 | 1.0099954 | 1.0052086 | 1.0148049 | 1.0150209 | 1.0106404 | 1.0194205 |
|  |  | 1999.5 | 0.9910668 | 0.9865108 | 0.9956438 | 0.9929781 | 0.9885714 | 0.9974043 | 1.0041131 | 1.0002195 | 1.0080219 |
|  |  | 2004.5 | 1 | 1 | 1 | 1 | 1 | 1 | 1 | 1 | 1 |
|  |  | 2009.5 | 0.9954132 | 0.9909244 | 0.9999223 | 0.9954913 | 0.9911359 | 0.9998658 | 0.9886575 | 0.9849028 | 0.9924265 |
|  |  | 2014.5 | 0.9868615 | 0.9820539 | 0.9916926 | 0.9874932 | 0.9828242 | 0.9921844 | 0.9751794 | 0.9710179 | 0.9793588 |
|  |  | 2019.5 | 0.9886325 | 0.9832583 | 0.994036 | 0.9863869 | 0.9811729 | 0.9916287 | 0.9571036 | 0.9523408 | 0.9618902 |
|  | cohort rate ratio | 1897 | 1.0995285 | 0.8913038 | 1.3563984 | 1.0913612 | 0.8974629 | 1.3271515 | 1.1855224 | 0.9134743 | 1.5385909 |
|  |  | 1902 | 1.1000761 | 1.0036222 | 1.2057998 | 1.0944357 | 1.0045046 | 1.1924181 | 1.1916383 | 1.0825901 | 1.3116708 |
|  |  | 1907 | 1.0930823 | 1.0428847 | 1.1456961 | 1.0866317 | 1.0369847 | 1.1386556 | 1.1828993 | 1.1318111 | 1.2362936 |
|  |  | 1912 | 1.0930747 | 1.0618842 | 1.1251813 | 1.0825935 | 1.0517118 | 1.114382 | 1.1691767 | 1.1390584 | 1.2000914 |
|  |  | 1917 | 1.0811058 | 1.0582331 | 1.104473 | 1.0709759 | 1.0480187 | 1.0944359 | 1.1287459 | 1.1077995 | 1.1500884 |
|  |  | 1922 | 1.0783279 | 1.0614619 | 1.0954619 | 1.068291 | 1.0514096 | 1.0854435 | 1.1145908 | 1.0999118 | 1.1294657 |
|  |  | 1927 | 1.0806637 | 1.0672819 | 1.0942134 | 1.0692232 | 1.056129 | 1.0824798 | 1.129797 | 1.1188386 | 1.1408628 |
|  |  | 1932 | 1.062154 | 1.0506469 | 1.073787 | 1.0583517 | 1.0470028 | 1.0698235 | 1.0907908 | 1.0820075 | 1.0996454 |
|  |  | 1937 | 1.0526533 | 1.042583 | 1.0628209 | 1.0487317 | 1.0387081 | 1.058852 | 1.0911842 | 1.0836433 | 1.0987775 |
|  |  | 1942 | 1.0345945 | 1.0253789 | 1.043893 | 1.0295697 | 1.0204135 | 1.0388081 | 1.041698 | 1.0350292 | 1.0484099 |
|  |  | 1947 | 1.0152349 | 1.0070999 | 1.0234357 | 1.0137636 | 1.0056297 | 1.0219633 | 1.0068591 | 1.0010682 | 1.0126835 |
|  |  | 1952 | 1 | 1 | 1 | 1 | 1 | 1 | 1 | 1 | 1 |
|  |  | 1957 | 1.0062741 | 0.9991189 | 1.0134805 | 1.0043762 | 0.9971679 | 1.0116365 | 0.997208 | 0.9921037 | 1.0023386 |
|  |  | 1962 | 0.9995113 | 0.9923659 | 1.0067082 | 0.9987613 | 0.991549 | 1.006026 | 0.9707952 | 0.9657074 | 0.9759099 |
|  |  | 1967 | 0.9748049 | 0.9676689 | 0.9819936 | 0.9751565 | 0.9679769 | 0.9823894 | 0.92614 | 0.9210866 | 0.9312211 |
|  |  | 1972 | 0.9897555 | 0.9822335 | 0.997335 | 0.9874903 | 0.9799362 | 0.9951026 | 0.941891 | 0.9364616 | 0.947352 |
|  |  | 1977 | 1.0196954 | 1.0115637 | 1.0278925 | 1.0134895 | 1.0053896 | 1.0216546 | 0.9753456 | 0.9693117 | 0.9814171 |
|  |  | 1982 | 1.0261409 | 1.0175607 | 1.0347933 | 1.0164085 | 1.0079386 | 1.0249495 | 0.9805747 | 0.9740484 | 0.9871447 |
|  |  | 1987 | 1.0118239 | 1.0030129 | 1.0207122 | 1.001543 | 0.9929249 | 1.010236 | 0.9579581 | 0.9511399 | 0.9648253 |
|  |  | 1992 | 1.0177652 | 1.0081396 | 1.0274827 | 1.0080313 | 0.9987386 | 1.0174105 | 0.9573913 | 0.9497045 | 0.9651404 |
|  |  | 1997 | 1.03408 | 1.023132 | 1.0451453 | 1.0233625 | 1.0129766 | 1.0338549 | 0.9724583 | 0.9632831 | 0.9817209 |
|  |  | 2002 | 1.0488288 | 1.0361256 | 1.0616877 | 1.039319 | 1.027639 | 1.0511318 | 0.9930102 | 0.9814646 | 1.0046916 |
|  |  | 2007 | 1.0417058 | 1.0261583 | 1.0574889 | 1.0350765 | 1.0217863 | 1.0485396 | 0.9940813 | 0.9780729 | 1.0103517 |
|  |  | 2012 | 1.0295759 | 1.0015592 | 1.0583763 | 1.0211513 | 1.002596 | 1.0400499 | 0.9876356 | 0.9552277 | 1.0211429 |
|  | local drifts | 7.5 | 0.0976904 | 0.0158838 | 0.179564 | 0.1096855 | 0.0560564 | 0.1633433 | 0.1637238 | 0.0633709 | 0.2641773 |
|  |  | 12.5 | 0.1137414 | 0.0697891 | 0.157713 | 0.124172 | 0.0876758 | 0.1606815 | 0.1096752 | 0.0599084 | 0.1594668 |
|  |  | 17.5 | 0.0970909 | 0.0622922 | 0.1319018 | 0.0873208 | 0.0558477 | 0.1188039 | 0.0367031 | 0.0011468 | 0.072272 |
|  |  | 22.5 | 0.113958 | 0.083723 | 0.1442021 | 0.0843087 | 0.0558764 | 0.1127491 | 0.0460759 | 0.0172935 | 0.0748666 |
|  |  | 27.5 | 0.1647599 | 0.137244 | 0.1922835 | 0.1206728 | 0.0942749 | 0.1470777 | 0.1269517 | 0.1020107 | 0.1518988 |
|  |  | 32.5 | 0.1400905 | 0.1135075 | 0.1666806 | 0.0938683 | 0.067807 | 0.1199364 | 0.079852 | 0.0569392 | 0.10277 |
|  |  | 37.5 | 0.0988782 | 0.0716352 | 0.1261287 | 0.066324 | 0.0391711 | 0.0934843 | -0.030401 | -0.052634 | -0.008163 |
|  |  | 42.5 | 0.0130495 | -0.015494 | 0.0416015 | -0.00445 | -0.033171 | 0.0242799 | -0.195874 | -0.217952 | -0.173792 |
|  |  | 47.5 | -0.120147 | -0.151249 | -0.089036 | -0.121281 | -0.152736 | -0.089815 | -0.336883 | -0.359906 | -0.313855 |
|  |  | 52.5 | -0.193076 | -0.227265 | -0.158875 | -0.178043 | -0.212514 | -0.143559 | -0.399276 | -0.423876 | -0.374671 |
|  |  | 57.5 | -0.20402 | -0.240629 | -0.167397 | -0.18959 | -0.226445 | -0.152722 | -0.41189 | -0.437898 | -0.385875 |
|  |  | 62.5 | -0.252853 | -0.292654 | -0.213036 | -0.239681 | -0.279283 | -0.200064 | -0.424412 | -0.453194 | -0.395621 |
|  |  | 67.5 | -0.308505 | -0.352248 | -0.264743 | -0.275181 | -0.318371 | -0.231973 | -0.511148 | -0.544646 | -0.477639 |
|  |  | 72.5 | -0.251763 | -0.304157 | -0.199341 | -0.219449 | -0.272163 | -0.166707 | -0.428482 | -0.472106 | -0.38484 |
|  |  | 77.5 | -0.176669 | -0.245541 | -0.107749 | -0.15006 | -0.220032 | -0.080038 | -0.285353 | -0.345788 | -0.224882 |
|  |  | 82.5 | -0.136647 | -0.2293 | -0.043908 | -0.110562 | -0.20377 | -0.017267 | -0.247832 | -0.331625 | -0.163968 |
|  |  | 87.5 | -0.103 | -0.249332 | 0.0435465 | -0.098033 | -0.243954 | 0.0481021 | -0.297119 | -0.434111 | -0.159939 |
|  |  | 92.5 | -0.080424 | -0.359137 | 0.1990676 | -0.101885 | -0.364333 | 0.1612544 | -0.273962 | -0.561983 | 0.0148938 |
|  |  | 97.5 | -0.085416 | -0.710826 | 0.5439344 | -0.100268 | -0.683207 | 0.4860927 | -0.275514 | -1.040366 | 0.4952494 |
| Middle SDI | longitudinal age curve | 7.5 | 6382.8556 | 6320.7158 | 6445.6064 | 5601.2582 | 5555.3637 | 5647.5317 | 7.3223983 | 7.2573278 | 7.3880523 |
|  |  | 12.5 | 21430.319 | 21265.995 | 21595.912 | 8990.4491 | 8922.9354 | 9058.4737 | 27.84836 | 27.679865 | 28.017881 |
|  |  | 17.5 | 24677.63 | 24496.993 | 24859.598 | 8939.0136 | 8873.706 | 9004.8018 | 44.715257 | 44.473779 | 44.958047 |
|  |  | 22.5 | 26268.759 | 26082.861 | 26455.981 | 9393.1201 | 9326.5012 | 9460.215 | 54.118546 | 53.843648 | 54.394847 |
|  |  | 27.5 | 27732.928 | 27543.298 | 27923.864 | 10256.862 | 10186.692 | 10327.516 | 56.670994 | 56.395182 | 56.948154 |
|  |  | 32.5 | 29476.27 | 29281.248 | 29672.591 | 10012.556 | 9945.676 | 10079.885 | 64.023983 | 63.726491 | 64.322864 |
|  |  | 37.5 | 28550.289 | 28365.053 | 28736.734 | 9574.0037 | 9511.2018 | 9637.2202 | 70.772065 | 70.455902 | 71.089647 |
|  |  | 42.5 | 28295.259 | 28126.454 | 28465.076 | 9442.9725 | 9385.9688 | 9500.3223 | 75.227065 | 74.921299 | 75.534079 |
|  |  | 47.5 | 26179.54 | 26022.356 | 26337.674 | 8228.3011 | 8177.8159 | 8279.0979 | 75.796654 | 75.491861 | 76.102677 |
|  |  | 52.5 | 23949.103 | 23800.623 | 24098.509 | 8120.0978 | 8069.5861 | 8170.9258 | 73.785132 | 73.483262 | 74.088243 |
|  |  | 57.5 | 23876.608 | 23721.645 | 24032.584 | 7401.6888 | 7352.3433 | 7451.3655 | 71.713661 | 71.405243 | 72.023411 |
|  |  | 62.5 | 21779.776 | 21622.763 | 21937.928 | 7491.5877 | 7437.8668 | 7545.6967 | 70.413707 | 70.085929 | 70.743018 |
|  |  | 67.5 | 26006.217 | 25806.364 | 26207.619 | 10086.15 | 10011.891 | 10160.961 | 70.509024 | 70.140334 | 70.879651 |
|  |  | 72.5 | 29187.471 | 28912.657 | 29464.897 | 9474.3371 | 9384.5551 | 9564.978 | 62.826981 | 62.397772 | 63.259143 |
|  |  | 77.5 | 25484.007 | 25187.977 | 25783.516 | 7741.3017 | 7648.8896 | 7834.8303 | 52.515957 | 52.058506 | 52.977429 |
|  |  | 82.5 | 23632.61 | 23277.594 | 23993.039 | 7843.5559 | 7725.2207 | 7963.7037 | 46.579093 | 46.033046 | 47.131617 |
|  |  | 87.5 | 22912.574 | 22418.389 | 23417.654 | 7429.0832 | 7266.3299 | 7595.4819 | 42.639352 | 41.885135 | 43.407151 |
|  |  | 92.5 | 23260.154 | 22427.544 | 24123.675 | 8954.6312 | 8650.1675 | 9269.8113 | 34.983178 | 33.830265 | 36.175381 |
|  |  | 97.5 | 31407.785 | 29553.821 | 33378.052 | 12053.204 | 11377.832 | 12768.665 | 32.74981 | 30.622628 | 35.024757 |
|  | period rate ratio | 1994.5 | 0.9891013 | 0.9848134 | 0.993408 | 0.9866855 | 0.9825567 | 0.9908316 | 0.9951747 | 0.9915631 | 0.9987995 |
|  |  | 1999.5 | 0.9842613 | 0.9805206 | 0.9880163 | 0.9857253 | 0.9821234 | 0.9893405 | 0.9907838 | 0.9877941 | 0.9937825 |
|  |  | 2004.5 | 1 | 1 | 1 | 1 | 1 | 1 | 1 | 1 | 1 |
|  |  | 2009.5 | 1.003828 | 1.0001427 | 1.0075269 | 1.0031033 | 0.9995444 | 1.0066748 | 1.0046208 | 1.0016992 | 1.007551 |
|  |  | 2014.5 | 1.0032374 | 0.9990097 | 1.007483 | 1.0030455 | 0.9989441 | 1.0071637 | 1.0057512 | 1.0021983 | 1.0093167 |
|  |  | 2019.5 | 1.0128704 | 1.0077859 | 1.0179806 | 1.0098781 | 1.0049397 | 1.0148408 | 1.0170367 | 1.0125561 | 1.0215372 |
|  | cohort rate ratio | 1897 | 1.0530102 | 0.8347047 | 1.3284104 | 1.0384358 | 0.8332811 | 1.2940999 | 1.0213823 | 0.7859729 | 1.3273 |
|  |  | 1902 | 1.0582855 | 0.9535293 | 1.1745504 | 1.044723 | 0.9466243 | 1.1529875 | 1.0231756 | 0.9237828 | 1.1332624 |
|  |  | 1907 | 1.0461606 | 0.9910678 | 1.104316 | 1.0401844 | 0.9856314 | 1.0977568 | 1.0127851 | 0.9657357 | 1.0621268 |
|  |  | 1912 | 1.0354314 | 1.0018921 | 1.0700934 | 1.0273224 | 0.9938462 | 1.0619262 | 1.0071474 | 0.9798501 | 1.0352051 |
|  |  | 1917 | 1.0357032 | 1.0132128 | 1.0586928 | 1.0268026 | 1.0038819 | 1.0502467 | 1.0109124 | 0.9932294 | 1.0289102 |
|  |  | 1922 | 1.0321367 | 1.0159046 | 1.0486281 | 1.0279048 | 1.0114112 | 1.0446674 | 1.0119398 | 0.9995664 | 1.0244664 |
|  |  | 1927 | 1.0237444 | 1.0110636 | 1.0365843 | 1.0252071 | 1.0126544 | 1.0379154 | 1.009293 | 1.0001269 | 1.0185431 |
|  |  | 1932 | 1.0082592 | 0.9976712 | 1.0189595 | 1.0176317 | 1.0070877 | 1.0282862 | 1.0046316 | 0.9972962 | 1.0120209 |
|  |  | 1937 | 0.9935447 | 0.9844621 | 1.002711 | 1.0022926 | 0.9931551 | 1.0115141 | 1.0000125 | 0.9938084 | 1.0062553 |
|  |  | 1942 | 0.9916042 | 0.983508 | 0.999767 | 0.9956222 | 0.9875063 | 1.0038048 | 0.9988332 | 0.9933782 | 1.0043182 |
|  |  | 1947 | 0.9913751 | 0.9842056 | 0.9985969 | 0.9947339 | 0.9875196 | 1.0020009 | 0.9962104 | 0.991412 | 1.0010319 |
|  |  | 1952 | 1 | 1 | 1 | 1 | 1 | 1 | 1 | 1 | 1 |
|  |  | 1957 | 1.0313812 | 1.0248576 | 1.0379463 | 1.0290151 | 1.0224269 | 1.0356458 | 1.0185226 | 1.0142086 | 1.022855 |
|  |  | 1962 | 1.0380087 | 1.0315276 | 1.0445306 | 1.0372142 | 1.0306457 | 1.0438245 | 1.0211853 | 1.0168714 | 1.0255176 |
|  |  | 1967 | 1.0275295 | 1.0210641 | 1.0340358 | 1.0282168 | 1.0216787 | 1.0347967 | 1.0146439 | 1.0102995 | 1.0190069 |
|  |  | 1972 | 1.0444286 | 1.0376638 | 1.0512376 | 1.0425346 | 1.0357056 | 1.0494086 | 1.0280711 | 1.0234823 | 1.0326805 |
|  |  | 1977 | 1.079374 | 1.0721228 | 1.0866742 | 1.0718536 | 1.0645988 | 1.0791579 | 1.0548763 | 1.0499126 | 1.0598634 |
|  |  | 1982 | 1.1017901 | 1.0941257 | 1.1095082 | 1.0921858 | 1.0845581 | 1.099867 | 1.0743364 | 1.0690107 | 1.0796888 |
|  |  | 1987 | 1.0972215 | 1.0893682 | 1.1051313 | 1.0885301 | 1.0807554 | 1.0963607 | 1.0704092 | 1.064854 | 1.0759934 |
|  |  | 1992 | 1.1144078 | 1.1060257 | 1.1228535 | 1.1062947 | 1.098067 | 1.1145841 | 1.0811155 | 1.0750742 | 1.0871907 |
|  |  | 1997 | 1.1353319 | 1.1261886 | 1.1445495 | 1.1276242 | 1.1187539 | 1.1365647 | 1.0963817 | 1.089597 | 1.1032087 |
|  |  | 2002 | 1.1408194 | 1.130719 | 1.15101 | 1.1327597 | 1.1232041 | 1.1423967 | 1.1043687 | 1.0964112 | 1.112384 |
|  |  | 2007 | 1.1331827 | 1.1213529 | 1.1451373 | 1.1285407 | 1.1179713 | 1.1392101 | 1.1033699 | 1.0929741 | 1.1138647 |
|  |  | 2012 | 1.1249688 | 1.1051658 | 1.1451266 | 1.1208661 | 1.1069952 | 1.1349109 | 1.0993429 | 1.0795559 | 1.1194926 |
|  | local drifts | 7.5 | 0.1028038 | 0.0514308 | 0.1542031 | 0.1204374 | 0.0860386 | 0.154848 | 0.1153486 | 0.0612751 | 0.1694514 |
|  |  | 12.5 | 0.1578211 | 0.1293671 | 0.1862832 | 0.1728949 | 0.1487969 | 0.1969987 | 0.1378378 | 0.1099989 | 0.1656843 |
|  |  | 17.5 | 0.2187167 | 0.1954951 | 0.2419438 | 0.222144 | 0.2007976 | 0.243495 | 0.1716561 | 0.1509947 | 0.1923218 |
|  |  | 22.5 | 0.2912496 | 0.2700914 | 0.3124122 | 0.27685 | 0.2566467 | 0.2970573 | 0.2240808 | 0.2064523 | 0.2417124 |
|  |  | 27.5 | 0.3287203 | 0.3083269 | 0.3491179 | 0.2942969 | 0.2744646 | 0.3141331 | 0.2612711 | 0.2450188 | 0.277526 |
|  |  | 32.5 | 0.2973739 | 0.2766033 | 0.3181488 | 0.2576162 | 0.2370393 | 0.2781974 | 0.2475176 | 0.2316538 | 0.2633839 |
|  |  | 37.5 | 0.2653404 | 0.2432 | 0.2874857 | 0.2347185 | 0.2125019 | 0.2569401 | 0.2158184 | 0.1996571 | 0.2319823 |
|  |  | 42.5 | 0.2342585 | 0.2102676 | 0.258255 | 0.2158859 | 0.191633 | 0.2401446 | 0.1650988 | 0.1483165 | 0.1818839 |
|  |  | 47.5 | 0.1993634 | 0.172477 | 0.2262571 | 0.1865101 | 0.1591798 | 0.2138479 | 0.1164276 | 0.0982636 | 0.1345948 |
|  |  | 52.5 | 0.1983344 | 0.1678381 | 0.22884 | 0.1802335 | 0.1493878 | 0.2110886 | 0.0978544 | 0.0776272 | 0.1180857 |
|  |  | 57.5 | 0.197655 | 0.1635089 | 0.2318128 | 0.1575475 | 0.1232033 | 0.1919036 | 0.0955408 | 0.0728458 | 0.1182411 |
|  |  | 62.5 | 0.0757806 | 0.0375932 | 0.1139825 | 0.0273511 | -0.01048 | 0.065197 | 0.0377183 | 0.0117968 | 0.0636466 |
|  |  | 67.5 | -0.097068 | -0.140256 | -0.053862 | -0.113892 | -0.156417 | -0.071349 | -0.041525 | -0.072552 | -0.010488 |
|  |  | 72.5 | -0.178049 | -0.230374 | -0.125696 | -0.152482 | -0.205517 | -0.099419 | -0.06523 | -0.105555 | -0.02489 |
|  |  | 77.5 | -0.19816 | -0.268549 | -0.12772 | -0.135508 | -0.208073 | -0.06289 | -0.057299 | -0.113962 | -0.000605 |
|  |  | 82.5 | -0.168545 | -0.272385 | -0.064597 | -0.087317 | -0.192638 | 0.0181144 | -0.032488 | -0.119101 | 0.0541998 |
|  |  | 87.5 | -0.126783 | -0.295045 | 0.0417624 | -0.065527 | -0.233735 | 0.1029647 | -0.018864 | -0.166065 | 0.1285533 |
|  |  | 92.5 | -0.117726 | -0.434737 | 0.2002953 | -0.074497 | -0.376478 | 0.2284 | -0.038324 | -0.345773 | 0.2700748 |
|  |  | 97.5 | -0.100022 | -0.792823 | 0.5976172 | -0.065872 | -0.722468 | 0.5950672 | -0.050384 | -0.823073 | 0.7283242 |
| Low-middle SDI | longitudinal age curve | 7.5 | 7663.3165 | 7621.6967 | 7705.1636 | 6725.589 | 6697.9329 | 6753.3593 | 8.3008453 | 8.2203315 | 8.3821477 |
|  |  | 12.5 | 25726.537 | 25609.617 | 25843.991 | 10690.949 | 10649.552 | 10732.507 | 31.324786 | 31.099292 | 31.551915 |
|  |  | 17.5 | 29758.665 | 29627.927 | 29889.98 | 10889.819 | 10848.492 | 10931.304 | 50.042056 | 49.710792 | 50.375527 |
|  |  | 22.5 | 32151.016 | 32013.245 | 32289.38 | 11416.184 | 11373.725 | 11458.802 | 60.523715 | 60.140526 | 60.909346 |
|  |  | 27.5 | 32982.998 | 32844.646 | 33121.932 | 11842.163 | 11799.042 | 11885.442 | 62.312715 | 61.928874 | 62.698936 |
|  |  | 32.5 | 33743.285 | 33604.851 | 33882.288 | 11307.103 | 11266.574 | 11347.778 | 68.337462 | 67.930257 | 68.747107 |
|  |  | 37.5 | 32490.008 | 32358.722 | 32621.826 | 10928.412 | 10889.839 | 10967.121 | 73.968158 | 73.540852 | 74.397946 |
|  |  | 42.5 | 32394.592 | 32274.048 | 32515.587 | 10866.031 | 10830.711 | 10901.467 | 78.306191 | 77.892111 | 78.722471 |
|  |  | 47.5 | 30224.767 | 30111.293 | 30338.668 | 9514.3057 | 9482.7698 | 9545.9465 | 80.49613 | 80.074603 | 80.919876 |
|  |  | 52.5 | 27656.874 | 27548.431 | 27765.744 | 9258.7857 | 9227.1993 | 9290.4803 | 79.325659 | 78.899997 | 79.753617 |
|  |  | 57.5 | 27304.22 | 27191.605 | 27417.301 | 8385.4701 | 8354.6702 | 8416.3835 | 77.132356 | 76.696746 | 77.57044 |
|  |  | 62.5 | 24767.337 | 24654.348 | 24880.844 | 8338.5365 | 8305.6003 | 8371.6033 | 75.970107 | 75.508644 | 76.43439 |
|  |  | 67.5 | 28261.792 | 28122.451 | 28401.824 | 10550.87 | 10507.214 | 10594.708 | 76.081521 | 75.562633 | 76.603972 |
|  |  | 72.5 | 30819.77 | 30632.734 | 31007.949 | 10003.708 | 9950.7257 | 10056.973 | 67.42385 | 66.823267 | 68.02983 |
|  |  | 77.5 | 28064.293 | 27852.793 | 28277.399 | 8738.0164 | 8679.7886 | 8796.6348 | 57.002181 | 56.347478 | 57.66449 |
|  |  | 82.5 | 26565.254 | 26301.498 | 26831.655 | 8595.8007 | 8521.0196 | 8671.2381 | 50.995372 | 50.192767 | 51.81081 |
|  |  | 87.5 | 24788.272 | 24423.514 | 25158.477 | 7844.5036 | 7742.3827 | 7947.9715 | 45.663404 | 44.556622 | 46.797679 |
|  |  | 92.5 | 25205.769 | 24595.272 | 25831.42 | 9749.1848 | 9556.308 | 9945.9545 | 36.96392 | 35.314967 | 38.689868 |
|  |  | 97.5 | 34562.384 | 33248.941 | 35927.712 | 13237.247 | 12826.01 | 13661.669 | 35.251267 | 32.346477 | 38.416913 |
|  | period rate ratio | 1994.5 | 1.0093336 | 1.0067225 | 1.0119516 | 1.0066199 | 1.0044616 | 1.0087829 | 1.0081189 | 1.0036197 | 1.0126382 |
|  |  | 1999.5 | 1.0012637 | 0.9990309 | 1.0035014 | 1.0013617 | 0.9995241 | 1.0032026 | 1.0031275 | 0.9994276 | 1.0068412 |
|  |  | 2004.5 | 1 | 1 | 1 | 1 | 1 | 1 | 1 | 1 | 1 |
|  |  | 2009.5 | 1.0004947 | 0.9983472 | 1.0026469 | 0.9997584 | 0.9979868 | 1.0015332 | 1.0022975 | 0.998743 | 1.0058646 |
|  |  | 2014.5 | 1.0012092 | 0.9986959 | 1.0037289 | 0.9998157 | 0.9977262 | 1.0019096 | 1.0055378 | 1.0011789 | 1.0099156 |
|  |  | 2019.5 | 1.0112717 | 1.0081797 | 1.0143733 | 1.0068229 | 1.0042432 | 1.0094092 | 1.0180888 | 1.0125362 | 1.0236719 |
|  | cohort rate ratio | 1897 | 0.9862346 | 0.8609965 | 1.1296894 | 0.9910839 | 0.8878049 | 1.1063774 | 0.9616696 | 0.7051576 | 1.3114919 |
|  |  | 1902 | 0.989996 | 0.9287293 | 1.0553044 | 0.993659 | 0.9436244 | 1.0463466 | 0.9683989 | 0.8529166 | 1.0995171 |
|  |  | 1907 | 0.9934745 | 0.9603971 | 1.0276912 | 0.9984576 | 0.9698244 | 1.0279361 | 0.9749085 | 0.9180688 | 1.0352673 |
|  |  | 1912 | 0.9930922 | 0.9724912 | 1.0141296 | 0.9995145 | 0.9814393 | 1.0179227 | 0.9791346 | 0.9451117 | 1.0143824 |
|  |  | 1917 | 0.995392 | 0.9812249 | 1.0097637 | 0.9989582 | 0.9863931 | 1.0116834 | 0.9865776 | 0.963901 | 1.0097878 |
|  |  | 1922 | 0.9966267 | 0.986305 | 1.0070564 | 1.001554 | 0.9924305 | 1.0107614 | 0.9895907 | 0.9736433 | 1.0057992 |
|  |  | 1927 | 0.9970535 | 0.9890123 | 1.0051602 | 1.001264 | 0.9943303 | 1.008246 | 0.9913727 | 0.9796007 | 1.003286 |
|  |  | 1932 | 0.9979688 | 0.991275 | 1.0047078 | 1.0029814 | 0.997196 | 1.0088004 | 0.994732 | 0.9853314 | 1.0042223 |
|  |  | 1937 | 0.9956921 | 0.9899722 | 1.001445 | 1.0024335 | 0.9974329 | 1.0074591 | 0.9957819 | 0.9878453 | 1.0037823 |
|  |  | 1942 | 0.9949897 | 0.9899327 | 1.0000727 | 1.0001787 | 0.9957674 | 1.0046095 | 0.9955707 | 0.9886153 | 1.002575 |
|  |  | 1947 | 0.9960186 | 0.9915004 | 1.0005574 | 0.9990681 | 0.9951176 | 1.0030342 | 0.9966327 | 0.9904147 | 1.0028897 |
|  |  | 1952 | 1 | 1 | 1 | 1 | 1 | 1 | 1 | 1 | 1 |
|  |  | 1957 | 1.0013104 | 0.9973235 | 1.0053133 | 1.0006543 | 0.9971648 | 1.0041561 | 1.0003201 | 0.9947809 | 1.00589 |
|  |  | 1962 | 1.0009961 | 0.9970635 | 1.0049443 | 1.0004034 | 0.9969561 | 1.0038626 | 1.000221 | 0.9946957 | 1.0057769 |
|  |  | 1967 | 0.9997157 | 0.9957574 | 1.0036898 | 0.9996124 | 0.9961533 | 1.0030835 | 1.0004318 | 0.9948198 | 1.0060754 |
|  |  | 1972 | 1.0000039 | 0.9959752 | 1.0040489 | 0.9998035 | 0.9962825 | 1.0033369 | 1.0018335 | 0.9960534 | 1.0076472 |
|  |  | 1977 | 1.0024295 | 0.9982926 | 1.0065834 | 1.0015155 | 0.9979142 | 1.0051297 | 1.0062908 | 1.0002713 | 1.0123466 |
|  |  | 1982 | 1.0015524 | 0.9973055 | 1.0058174 | 1.0010965 | 0.9974082 | 1.0047985 | 1.0078451 | 1.0015695 | 1.0141599 |
|  |  | 1987 | 1.0013485 | 0.9970014 | 1.0057146 | 0.998748 | 0.9949994 | 1.0025108 | 1.0094819 | 1.0029565 | 1.0160498 |
|  |  | 1992 | 1.0011318 | 0.9966468 | 1.0056369 | 0.9993507 | 0.9955052 | 1.0032111 | 1.0103241 | 1.0034737 | 1.0172211 |
|  |  | 1997 | 0.9996641 | 0.9949953 | 1.0043547 | 0.9990835 | 0.9951128 | 1.0030702 | 1.0111604 | 1.0038478 | 1.0185263 |
|  |  | 2002 | 0.9960546 | 0.9910725 | 1.0010618 | 0.9965051 | 0.9923505 | 1.000677 | 1.0124773 | 1.0042772 | 1.0207443 |
|  |  | 2007 | 0.987802 | 0.98216 | 0.9934765 | 0.9913973 | 0.9869115 | 0.9959034 | 1.0091897 | 0.998976 | 1.0195078 |
|  |  | 2012 | 0.9804855 | 0.9716783 | 0.9893725 | 0.9818961 | 0.9763249 | 0.9874992 | 1.004203 | 0.9859861 | 1.0227564 |
|  | local drifts | 7.5 | -0.085166 | -0.110472 | -0.059854 | -0.063774 | -0.078489 | -0.049058 | -0.016161 | -0.069866 | 0.0375729 |
|  |  | 12.5 | -0.049411 | -0.063766 | -0.035054 | -0.031819 | -0.042296 | -0.02134 | 0.0093618 | -0.019082 | 0.0378136 |
|  |  | 17.5 | -0.021584 | -0.033548 | -0.009619 | -0.017434 | -0.026922 | -0.007944 | 0.0236206 | 0.0019376 | 0.0453083 |
|  |  | 22.5 | -0.003308 | -0.014706 | 0.0080906 | -0.007109 | -0.016543 | 0.0023249 | 0.0342667 | 0.0148316 | 0.0537057 |
|  |  | 27.5 | 0.0058476 | -0.005777 | 0.0174739 | -0.002798 | -0.012616 | 0.0070209 | 0.0420413 | 0.022999 | 0.0610871 |
|  |  | 32.5 | 0.0055368 | -0.006727 | 0.0178017 | -0.001211 | -0.011738 | 0.0093173 | 0.0415338 | 0.0222033 | 0.060868 |
|  |  | 37.5 | 0.0033081 | -0.01 | 0.0166182 | 0.0032761 | -0.008261 | 0.0148141 | 0.0325898 | 0.012471 | 0.0527127 |
|  |  | 42.5 | 0.0039633 | -0.010741 | 0.0186699 | 0.0024164 | -0.010416 | 0.0152507 | 0.0206317 | -0.000769 | 0.0420371 |
|  |  | 47.5 | 0.0107431 | -0.005873 | 0.0273618 | 0.0012945 | -0.013306 | 0.015897 | 0.0155556 | -0.007834 | 0.0389506 |
|  |  | 52.5 | 0.0228353 | 0.0039958 | 0.0416783 | 0.0010454 | -0.015463 | 0.0175563 | 0.0202626 | -0.005695 | 0.0462269 |
|  |  | 57.5 | 0.0283188 | 0.0071226 | 0.0495195 | -0.004444 | -0.022971 | 0.0140859 | 0.022797 | -0.006289 | 0.0518915 |
|  |  | 62.5 | 0.017544 | -0.006534 | 0.0416278 | -0.011438 | -0.03222 | 0.0093488 | 0.0238639 | -0.009541 | 0.0572801 |
|  |  | 67.5 | 0.0046745 | -0.023199 | 0.0325554 | -0.011597 | -0.035466 | 0.0122781 | 0.0279116 | -0.012502 | 0.068341 |
|  |  | 72.5 | -0.006601 | -0.040955 | 0.0277657 | -0.009271 | -0.039293 | 0.0207588 | 0.0281103 | -0.024951 | 0.0811995 |
|  |  | 77.5 | -0.002239 | -0.048501 | 0.0440442 | 0.0059729 | -0.034936 | 0.0468983 | 0.0385583 | -0.036261 | 0.1134335 |
|  |  | 82.5 | 0.0121476 | -0.054822 | 0.0791622 | 0.0150578 | -0.043593 | 0.0737432 | 0.0633278 | -0.049217 | 0.1759993 |
|  |  | 87.5 | 0.0204309 | -0.086177 | 0.1271529 | 0.0173984 | -0.074609 | 0.1094905 | 0.0805821 | -0.107139 | 0.2686562 |
|  |  | 92.5 | 0.0270519 | -0.169232 | 0.2237214 | 0.0267776 | -0.133397 | 0.1872094 | 0.0969889 | -0.28768 | 0.4831414 |
|  |  | 97.5 | 0.0390547 | -0.369877 | 0.4496648 | 0.0397559 | -0.291749 | 0.3723628 | 0.116194 | -0.804482 | 1.0454157 |
| Low SDI | longitudinal age curve | 7.5 | 6421.0289 | 6401.2378 | 6440.8811 | 5619.5798 | 5602.4521 | 5636.7598 | 7.0577008 | 6.955837 | 7.1610564 |
|  |  | 12.5 | 22232.111 | 22172.663 | 22291.719 | 9423.3112 | 9395.9332 | 9450.769 | 28.210832 | 27.895269 | 28.529965 |
|  |  | 17.5 | 26393.35 | 26324.714 | 26462.166 | 9724.7665 | 9696.9333 | 9752.6797 | 47.874567 | 47.376844 | 48.377519 |
|  |  | 22.5 | 29005.108 | 28931.178 | 29079.227 | 10524.497 | 10494.859 | 10554.218 | 58.785058 | 58.196328 | 59.379743 |
|  |  | 27.5 | 31067.968 | 30990.235 | 31145.896 | 11375.213 | 11343.742 | 11406.772 | 61.109228 | 60.510486 | 61.713895 |
|  |  | 32.5 | 32515.211 | 32435.299 | 32595.32 | 11032.068 | 11001.894 | 11062.324 | 67.581987 | 66.937273 | 68.232911 |
|  |  | 37.5 | 31599.427 | 31522.587 | 31676.453 | 10730 | 10700.956 | 10759.123 | 74.105937 | 73.416914 | 74.801426 |
|  |  | 42.5 | 31694.225 | 31622.883 | 31765.728 | 10771.658 | 10744.686 | 10798.698 | 79.222879 | 78.544198 | 79.907423 |
|  |  | 47.5 | 29722.364 | 29654.685 | 29790.197 | 9411.7349 | 9387.6402 | 9435.8915 | 81.918128 | 81.220316 | 82.621935 |
|  |  | 52.5 | 25975.651 | 25912.931 | 26038.523 | 8269.9547 | 8247.5239 | 8292.4466 | 80.358889 | 79.653214 | 81.070816 |
|  |  | 57.5 | 23258.15 | 23196.831 | 23319.632 | 6960.6415 | 6939.6324 | 6981.7142 | 75.938178 | 75.225446 | 76.657662 |
|  |  | 62.5 | 20891.824 | 20829.761 | 20954.072 | 7212.9872 | 7189.5377 | 7236.5133 | 75.006535 | 74.241107 | 75.779855 |
|  |  | 67.5 | 25285.727 | 25205.402 | 25366.309 | 9921.4152 | 9888.2115 | 9954.7305 | 75.873901 | 75.000736 | 76.757232 |
|  |  | 72.5 | 29039 | 28926.672 | 29151.765 | 9617.5586 | 9576.4005 | 9658.8935 | 67.844554 | 66.82304 | 68.881683 |
|  |  | 77.5 | 26411.991 | 26283.393 | 26541.217 | 8277.0015 | 8231.6545 | 8322.5982 | 57.15041 | 56.023254 | 58.300245 |
|  |  | 82.5 | 24979.032 | 24814.758 | 25144.393 | 8164.8553 | 8105.0525 | 8225.0995 | 50.749165 | 49.337411 | 52.201316 |
|  |  | 87.5 | 23407.323 | 23169.566 | 23647.521 | 7511.2099 | 7425.4829 | 7597.9266 | 45.076332 | 43.058714 | 47.188491 |
|  |  | 92.5 | 24072.015 | 23641.629 | 24510.235 | 9418.8583 | 9244.5708 | 9596.4315 | 35.93346 | 32.762848 | 39.410907 |
|  |  | 97.5 | 33261.728 | 32202.939 | 34355.329 | 12861.175 | 12437.26 | 13299.539 | 33.663755 | 27.538425 | 41.151535 |
|  | period rate ratio | 1994.5 | 1.0124779 | 1.0108079 | 1.0141506 | 1.009782 | 1.0080327 | 1.0115345 | 1.0096205 | 1.0015581 | 1.0177479 |
|  |  | 1999.5 | 1.0064425 | 1.0051022 | 1.0077846 | 1.0052865 | 1.0038954 | 1.0066795 | 1.0068888 | 1.0007681 | 1.013047 |
|  |  | 2004.5 | 1 | 1 | 1 | 1 | 1 | 1 | 1 | 1 | 1 |
|  |  | 2009.5 | 0.9984837 | 0.9972168 | 0.9997523 | 0.9986023 | 0.997284 | 0.9999223 | 1.0013798 | 0.9955611 | 1.0072325 |
|  |  | 2014.5 | 0.9979747 | 0.9963828 | 0.9995691 | 0.9981843 | 0.9965067 | 0.9998648 | 1.0046889 | 0.9968878 | 1.0125511 |
|  |  | 2019.5 | 1.0040444 | 1.0019825 | 1.0061106 | 1.0032633 | 1.0010765 | 1.0054548 | 1.0115435 | 1.0011025 | 1.0220934 |
|  | cohort rate ratio | 1897 | 1.0111753 | 0.9092463 | 1.1245308 | 1.0014447 | 0.897144 | 1.1178713 | 0.9936012 | 0.5074737 | 1.9454079 |
|  |  | 1902 | 1.0133941 | 0.9649276 | 1.064295 | 1.0045469 | 0.9550526 | 1.0566062 | 0.995355 | 0.7629518 | 1.2985507 |
|  |  | 1907 | 1.0164597 | 0.9917504 | 1.0417846 | 1.0098463 | 0.9828994 | 1.037532 | 0.993522 | 0.88302 | 1.1178524 |
|  |  | 1912 | 1.0113035 | 0.9970819 | 1.0257278 | 1.0078079 | 0.9920652 | 1.0238004 | 0.9933755 | 0.9315861 | 1.0592631 |
|  |  | 1917 | 1.0076143 | 0.9983919 | 1.0169218 | 1.0041495 | 0.9938004 | 1.0146063 | 0.9961165 | 0.9571488 | 1.0366706 |
|  |  | 1922 | 1.0077518 | 1.0011429 | 1.0144043 | 1.0051274 | 0.9977651 | 1.0125441 | 0.9984532 | 0.9714712 | 1.0261846 |
|  |  | 1927 | 1.0082425 | 1.0030268 | 1.0134854 | 1.0049685 | 0.9993391 | 1.0106296 | 1.0001648 | 0.9801166 | 1.020623 |
|  |  | 1932 | 1.0120525 | 1.0076764 | 1.0164476 | 1.0091861 | 1.004447 | 1.0139476 | 1.0032712 | 0.9873047 | 1.0194959 |
|  |  | 1937 | 1.0120912 | 1.0083513 | 1.015845 | 1.0127467 | 1.0086194 | 1.0168908 | 1.0055018 | 0.9920678 | 1.0191178 |
|  |  | 1942 | 1.0073961 | 1.0041488 | 1.0106539 | 1.0092337 | 1.0056286 | 1.0128517 | 1.0039779 | 0.992331 | 1.0157616 |
|  |  | 1947 | 1.0030844 | 1.0002311 | 1.0059458 | 1.0042716 | 1.0010947 | 1.0074587 | 1.0004182 | 0.9900844 | 1.0108598 |
|  |  | 1952 | 1 | 1 | 1 | 1 | 1 | 1 | 1 | 1 | 1 |
|  |  | 1957 | 0.9983854 | 0.9959273 | 1.0008496 | 0.9978957 | 0.9951507 | 1.0006482 | 0.9989242 | 0.9898227 | 1.0081093 |
|  |  | 1962 | 0.9981805 | 0.995779 | 1.0005879 | 0.9981153 | 0.9954266 | 1.0008112 | 0.9982976 | 0.9892795 | 1.0073979 |
|  |  | 1967 | 0.9966993 | 0.9943113 | 0.999093 | 0.997295 | 0.9946304 | 0.9999668 | 1.0001824 | 0.9910965 | 1.0093516 |
|  |  | 1972 | 0.9936411 | 0.9912399 | 0.996048 | 0.9945887 | 0.9919129 | 0.9972718 | 1.0025748 | 0.9932994 | 1.0119367 |
|  |  | 1977 | 0.9894103 | 0.9869729 | 0.9918538 | 0.9914636 | 0.9887528 | 0.9941818 | 0.9999505 | 0.9904198 | 1.0095728 |
|  |  | 1982 | 0.9859936 | 0.9835049 | 0.9884886 | 0.9884823 | 0.9857208 | 0.9912516 | 0.9983218 | 0.9884612 | 1.0082808 |
|  |  | 1987 | 0.9839568 | 0.9814251 | 0.9864951 | 0.9854444 | 0.9826504 | 0.9882464 | 0.9988847 | 0.9887063 | 1.0091679 |
|  |  | 1992 | 0.9812685 | 0.9786822 | 0.9838617 | 0.9827953 | 0.9799574 | 0.9856415 | 0.9992783 | 0.9887152 | 1.0099542 |
|  |  | 1997 | 0.9805376 | 0.9778748 | 0.9832076 | 0.9820346 | 0.9791348 | 0.984943 | 0.9999486 | 0.9888645 | 1.0111569 |
|  |  | 2002 | 0.9792621 | 0.9764737 | 0.9820585 | 0.9809319 | 0.9779428 | 0.9839303 | 1.0029056 | 0.9908144 | 1.0151444 |
|  |  | 2007 | 0.9733646 | 0.970308 | 0.9764307 | 0.9771881 | 0.9740349 | 0.9803515 | 1.0008468 | 0.9864179 | 1.0154867 |
|  |  | 2012 | 0.9650621 | 0.9607027 | 0.9694413 | 0.9678852 | 0.9641768 | 0.9716078 | 0.9946149 | 0.9707535 | 1.0190628 |
|  | local drifts | 7.5 | -0.069982 | -0.082224 | -0.057739 | -0.061801 | -0.071042 | -0.052559 | -0.007863 | -0.078159 | 0.0624825 |
|  |  | 12.5 | -0.045446 | -0.052798 | -0.038094 | -0.041135 | -0.047975 | -0.034295 | 0.0144883 | -0.024423 | 0.0534145 |
|  |  | 17.5 | -0.040524 | -0.046889 | -0.034159 | -0.043259 | -0.049669 | -0.036849 | 0.0114482 | -0.019255 | 0.0421613 |
|  |  | 22.5 | -0.053261 | -0.05952 | -0.047003 | -0.053092 | -0.059649 | -0.046535 | -0.008324 | -0.036826 | 0.0201856 |
|  |  | 27.5 | -0.063326 | -0.069854 | -0.056799 | -0.059382 | -0.066363 | -0.052402 | -0.009836 | -0.038595 | 0.0189312 |
|  |  | 32.5 | -0.061938 | -0.068949 | -0.054927 | -0.053503 | -0.061135 | -0.04587 | -0.00301 | -0.032873 | 0.0268622 |
|  |  | 37.5 | -0.052555 | -0.060283 | -0.044827 | -0.040087 | -0.048594 | -0.031579 | 0.0024778 | -0.029096 | 0.0340611 |
|  |  | 42.5 | -0.039424 | -0.048107 | -0.030741 | -0.03065 | -0.040298 | -0.021001 | 0.00719 | -0.02686 | 0.0412518 |
|  |  | 47.5 | -0.032805 | -0.042852 | -0.022757 | -0.032194 | -0.04349 | -0.020896 | 0.0061067 | -0.031729 | 0.0439571 |
|  |  | 52.5 | -0.039817 | -0.051517 | -0.028116 | -0.045734 | -0.058896 | -0.032571 | -0.015074 | -0.057688 | 0.0275584 |
|  |  | 57.5 | -0.056689 | -0.070186 | -0.04319 | -0.063362 | -0.078437 | -0.048285 | -0.02943 | -0.077911 | 0.0190737 |
|  |  | 62.5 | -0.061882 | -0.077381 | -0.046381 | -0.056659 | -0.073618 | -0.039697 | -0.023839 | -0.08006 | 0.0324134 |
|  |  | 67.5 | -0.04136 | -0.059253 | -0.023465 | -0.024512 | -0.043839 | -0.00518 | -0.006219 | -0.074585 | 0.0621939 |
|  |  | 72.5 | -0.01468 | -0.03645 | 0.0070943 | 0.0068393 | -0.017267 | 0.0309514 | 0.0134109 | -0.076121 | 0.103023 |
|  |  | 77.5 | 0.0089028 | -0.020666 | 0.0384807 | 0.0297732 | -0.00354 | 0.063098 | 0.0362987 | -0.091707 | 0.1644682 |
|  |  | 82.5 | 0.0100376 | -0.034777 | 0.0548729 | 0.0224565 | -0.027621 | 0.0725593 | 0.0479259 | -0.154419 | 0.2506805 |
|  |  | 87.5 | -0.017532 | -0.094059 | 0.0590537 | -0.006149 | -0.090552 | 0.0783257 | 0.0409235 | -0.322639 | 0.4058118 |
|  |  | 92.5 | -0.031394 | -0.180805 | 0.1182404 | -0.008908 | -0.16428 | 0.1467052 | 0.0238378 | -0.773414 | 0.827495 |
|  |  | 97.5 | -0.022399 | -0.34198 | 0.2982073 | 0.008655 | -0.322171 | 0.3405788 | 0.0151459 | -1.962256 | 2.032432 |

# **Table S8. Age-Standardized Rates and Frontier Analysis Results for Tension-Type Headache by Country, 2021**

| **location** | **SDI** | **Prevalence** | | | | **Incidence** | | | | **YLDs** | | | |
| --- | --- | --- | --- | --- | --- | --- | --- | --- | --- | --- | --- | --- | --- |
|  |  | **ASR** | **frontier** | **Effective difference** | **Effective difference rank(ASR rank)** | **ASR** | **frontier** | **Effective difference** | **Effective difference rank(ASR rank)** | **ASR** | **frontier** | **Effective difference** | **Effective difference rank(ASR rank)** |
| Afghanistan | 0.337199998 | 23578.37724 | 15838.40884 | 7739.9684 | 136 (137) | 8520.938228 | 6306.463609 | 2214.474619 | 68 (67) | 65.57413892 | 40.0007628 | 25.57337611 | 134 (134) |
| Albania | 0.706849791 | 29928.9961 | 15838.18291 | 14090.81319 | 53 (53) | 10567.45952 | 6306.364381 | 4261.095142 | 150 (150) | 72.98723942 | 40.00727289 | 32.97996653 | 186 (186) |
| Algeria | 0.659500924 | 23517.68057 | 15837.57412 | 7680.106454 | 150 (151) | 8501.42427 | 6306.392886 | 2195.031384 | 56 (55) | 66.65326296 | 40.0061401 | 26.64712285 | 146 (146) |
| American Samoa | 0.723727533 | 22152.00425 | 15838.30999 | 6313.694265 | 176 (176) | 8000.517936 | 6306.691483 | 1693.826453 | 28 (28) | 46.25614816 | 40.00255855 | 6.253589615 | 11 (10) |
| Andorra | 0.869444113 | 32352.91256 | 15837.81142 | 16515.10114 | 22 (22) | 10985.72494 | 6306.469562 | 4679.255378 | 180 (180) | 69.04316358 | 40.00307094 | 29.04009264 | 162 (162) |
| Angola | 0.453721949 | 23244.25178 | 15839.59847 | 7404.65331 | 155 (156) | 8350.797609 | 6306.268115 | 2044.529495 | 51 (50) | 54.40432017 | 40.00489014 | 14.39943003 | 94 (93) |
| Antigua and Barbuda | 0.749886887 | 23728.21036 | 15837.50582 | 7890.704543 | 124 (125) | 8525.858927 | 6306.425919 | 2219.433008 | 79 (79) | 51.47364158 | 40.00021899 | 11.4734226 | 71 (70) |
| Argentina | 0.723122973 | 26270.9643 | 15838.6169 | 10432.3474 | 68 (68) | 9486.562393 | 6306.384882 | 3180.177511 | 138 (138) | 60.01574319 | 40.00279963 | 20.01294356 | 121 (121) |
| Armenia | 0.701833194 | 30276.13426 | 15840.32285 | 14435.81141 | 40 (39) | 10665.43048 | 6306.276433 | 4359.154046 | 168 (168) | 67.18444048 | 40.00539227 | 27.17904821 | 157 (157) |
| Australia | 0.844252814 | 26964.15066 | 15837.64465 | 11126.50601 | 64 (64) | 9688.931866 | 6307.082061 | 3381.849805 | 142 (142) | 60.3757758 | 39.99679772 | 20.37897808 | 123 (123) |
| Austria | 0.853837004 | 31759.66748 | 15837.07314 | 15922.59434 | 25 (25) | 10935.22749 | 6306.161148 | 4629.066344 | 178 (178) | 71.22133615 | 40.00075836 | 31.22057779 | 176 (176) |
| Azerbaijan | 0.694851274 | 30266.51998 | 15837.24924 | 14429.27074 | 42 (42) | 10663.62829 | 6306.550796 | 4357.077497 | 165 (165) | 67.03563943 | 39.99581797 | 27.03982146 | 154 (154) |
| Bahamas | 0.805020668 | 23748.97976 | 15836.73932 | 7912.24044 | 110 (111) | 8532.978413 | 6306.539716 | 2226.438697 | 95 (94) | 51.72893048 | 39.99961339 | 11.72931709 | 77 (76) |
| Bahrain | 0.753043204 | 23559.14707 | 15839.68579 | 7719.461283 | 137 (138) | 8521.97697 | 6306.944639 | 2215.032331 | 70 (69) | 64.35571594 | 40.00219023 | 24.3535257 | 131 (131) |
| Bangladesh | 0.492420885 | 24771.43538 | 15839.04225 | 8932.39313 | 77 (77) | 8912.687845 | 6306.603572 | 2606.084273 | 128 (128) | 48.87315942 | 40.00272992 | 8.870429494 | 31 (30) |
| Barbados | 0.746748764 | 23735.70663 | 15838.41011 | 7897.296525 | 120 (122) | 8528.501154 | 6306.345083 | 2222.156071 | 83 (83) | 51.67580112 | 40.00118978 | 11.67461134 | 75 (74) |
| Belarus | 0.784484711 | 30340.65438 | 15837.71752 | 14502.93687 | 32 (32) | 10613.18594 | 6306.499757 | 4306.686183 | 162 (163) | 88.01138472 | 40.00128666 | 48.01009806 | 202 (202) |
| Belgium | 0.853654016 | 32396.92106 | 15839.01325 | 16557.90781 | 18 (18) | 10998.80615 | 6306.703845 | 4692.102304 | 184 (184) | 69.4154749 | 40.00321161 | 29.41226328 | 166 (166) |
| Belize | 0.610229002 | 23720.47625 | 15837.52272 | 7882.953523 | 128 (129) | 8524.309641 | 6306.559864 | 2217.749777 | 75 (74) | 51.2589918 | 39.99854686 | 11.26044494 | 64 (63) |
| Benin | 0.373486574 | 24550.24517 | 15838.65897 | 8711.5862 | 92 (91) | 8693.17788 | 6306.567164 | 2386.610715 | 118 (119) | 55.54721007 | 40.00030229 | 15.54690778 | 106 (106) |
| Bermuda | 0.821365422 | 23741.32872 | 15837.77261 | 7903.556116 | 114 (115) | 8530.664342 | 6306.716266 | 2223.948075 | 90 (89) | 51.86543074 | 40.00227085 | 11.86315989 | 81 (80) |
| Bhutan | 0.473062378 | 24804.70007 | 15836.79267 | 8967.907402 | 76 (76) | 8924.364085 | 6306.245263 | 2618.118822 | 129 (129) | 48.58360291 | 39.9986683 | 8.584934614 | 28 (27) |
| Bolivia (Plurinational State of) | 0.599010799 | 20238.67986 | 15837.74215 | 4400.937709 | 187 (187) | 7487.861356 | 6306.339902 | 1181.521454 | 19 (19) | 47.30467164 | 40.00273935 | 7.301932287 | 26 (25) |
| Bosnia and Herzegovina | 0.723077893 | 29958.94067 | 15839.57465 | 14119.36601 | 48 (47) | 10575.5138 | 6306.6005 | 4268.913304 | 154 (155) | 72.69158637 | 40.0042678 | 32.68731858 | 182 (182) |
| Botswana | 0.642721629 | 23234.87218 | 15836.70721 | 7398.164976 | 159 (160) | 8347.819723 | 6306.774209 | 2041.045515 | 46 (45) | 54.15017037 | 40.00174611 | 14.14842426 | 92 (91) |
| Brazil | 0.653043887 | 29095.40852 | 15837.50203 | 13257.9065 | 59 (59) | 10349.77507 | 6307.018318 | 4042.756757 | 147 (147) | 56.11831321 | 40.00181343 | 16.11649978 | 115 (115) |
| Brunei Darussalam | 0.810234367 | 28928.89963 | 15837.34076 | 13091.55887 | 60 (60) | 10119.3926 | 6306.537009 | 3812.85559 | 144 (144) | 60.39634331 | 40.00417537 | 20.39216794 | 124 (124) |
| Bulgaria | 0.768150939 | 29955.10064 | 15837.84271 | 14117.25794 | 49 (50) | 10575.39511 | 6306.437816 | 4268.957298 | 155 (154) | 72.81010609 | 39.99980781 | 32.81029828 | 184 (184) |
| Burkina Faso | 0.285118402 | 24561.02545 | 15838.2204 | 8722.805055 | 86 (86) | 8695.693675 | 6306.635979 | 2389.057696 | 122 (122) | 55.77041194 | 39.9967525 | 15.77365944 | 111 (111) |
| Burundi | 0.289374365 | 19503.76487 | 15838.65293 | 3665.111942 | 193 (194) | 7207.766042 | 6306.835902 | 900.9301397 | 16 (16) | 49.22530693 | 39.99884223 | 9.226464692 | 40 (39) |
| Cabo Verde | 0.533534539 | 24530.57898 | 15838.17021 | 8692.408779 | 100 (101) | 8689.4047 | 6306.54072 | 2382.86398 | 111 (110) | 55.62816153 | 40.00584883 | 15.62231269 | 109 (109) |
| Cambodia | 0.473621491 | 24628.97489 | 15839.65786 | 8789.317028 | 79 (79) | 8699.400197 | 6306.209325 | 2393.190872 | 124 (124) | 48.93503533 | 40.00370681 | 8.931328517 | 33 (32) |
| Cameroon | 0.479691223 | 24535.4766 | 15836.81172 | 8698.664883 | 95 (96) | 8689.354891 | 6306.661435 | 2382.693456 | 110 (109) | 55.45167006 | 40.00498629 | 15.44668377 | 105 (105) |
| Canada | 0.87317068 | 32911.94038 | 15836.94297 | 17074.99741 | 9 (9) | 11154.29224 | 6306.424271 | 4847.867972 | 196 (196) | 67.31211955 | 40.00172162 | 27.31039793 | 158 (158) |
| Central African Republic | 0.30916769 | 23237.17999 | 15837.69682 | 7399.483163 | 158 (159) | 8348.662967 | 6306.42493 | 2042.238037 | 48 (47) | 53.86395947 | 40.00066592 | 13.86329355 | 88 (87) |
| Chad | 0.240436019 | 24531.89088 | 15836.71608 | 8695.174794 | 99 (100) | 8688.963621 | 6308.593066 | 2380.370554 | 107 (108) | 54.93648967 | 39.99982392 | 14.93666575 | 98 (97) |
| Chile | 0.771514716 | 26244.3418 | 15836.92127 | 10407.42053 | 69 (69) | 9478.382671 | 6306.297867 | 3172.084804 | 137 (137) | 59.8322663 | 40.00004596 | 19.83222035 | 120 (120) |
| China | 0.72162976 | 18525.06694 | 15838.2474 | 2686.819547 | 200 (201) | 6851.134225 | 6306.604118 | 544.5301068 | 5 (4) | 43.49815401 | 39.99561072 | 3.502543293 | 5 (4) |
| Colombia | 0.655442913 | 23732.07197 | 15837.24354 | 7894.828428 | 123 (124) | 8527.893613 | 6306.589047 | 2221.304566 | 82 (81) | 51.69872017 | 40.00021827 | 11.6985019 | 76 (75) |
| Comoros | 0.475978688 | 19504.2583 | 15838.50313 | 3665.755171 | 191 (191) | 7206.988318 | 6306.2382 | 900.7501188 | 15 (14) | 49.79696333 | 40.00209488 | 9.794868446 | 47 (47) |
| Congo | 0.583075236 | 23228.54597 | 15838.4805 | 7390.065473 | 161 (161) | 8346.370045 | 6306.4712 | 2039.898845 | 44 (43) | 54.11344256 | 40.00056898 | 14.11287358 | 90 (89) |
| Cook Islands | 0.779109955 | 22226.15825 | 15837.2886 | 6388.869652 | 166 (166) | 8024.798682 | 6306.384235 | 1718.414448 | 38 (38) | 46.86220805 | 40.00270647 | 6.859501586 | 24 (23) |
| Costa Rica | 0.700340477 | 23742.7452 | 15837.70384 | 7905.041355 | 113 (114) | 8530.68245 | 6306.712132 | 2223.970319 | 91 (90) | 51.76023273 | 40.00008882 | 11.76014391 | 78 (77) |
| Croatia | 0.798341027 | 29953.92444 | 15839.1368 | 14114.78764 | 51 (51) | 10594.00092 | 6306.305655 | 4287.695268 | 159 (159) | 73.0782463 | 40.00535068 | 33.07289562 | 189 (189) |
| Cuba | 0.668729864 | 23717.58622 | 15838.57609 | 7879.010136 | 131 (131) | 8523.407818 | 6306.509241 | 2216.898577 | 74 (72) | 51.42896195 | 40.00395614 | 11.42500581 | 70 (69) |
| Cyprus | 0.835630545 | 32411.95373 | 15837.93932 | 16574.01442 | 13 (13) | 11000.7739 | 6306.762372 | 4694.011525 | 188 (188) | 69.90824263 | 40.00214394 | 29.90609869 | 171 (171) |
| Czechia | 0.828450433 | 29944.14824 | 15838.40934 | 14105.7389 | 52 (52) | 10572.78326 | 6306.346732 | 4266.436524 | 152 (152) | 72.74849529 | 39.99920914 | 32.74928615 | 183 (183) |
| Côte d'Ivoire | 0.425941883 | 24501.68676 | 15840.14212 | 8661.544637 | 105 (106) | 8681.400915 | 6306.371259 | 2375.029655 | 102 (102) | 55.01636823 | 39.9998218 | 15.01654643 | 99 (98) |
| Democratic People's Republic of Korea | 0.569854634 | 17560.908 | 15838.654 | 1722.253993 | 201 (202) | 6543.766382 | 6306.135221 | 237.6311613 | 4 (3) | 40.50674034 | 40.00204053 | 0.50469981 | 3 (2) |
| Democratic Republic of the Congo | 0.383179849 | 23228.07268 | 15837.96792 | 7390.104758 | 160 (162) | 8346.566638 | 6306.583305 | 2039.983334 | 45 (44) | 53.83286301 | 40.00335518 | 13.82950783 | 87 (86) |
| Denmark | 0.896424204 | 33764.78298 | 15837.4016 | 17927.38137 | 5 (5) | 11052.65667 | 6306.387066 | 4746.269606 | 193 (193) | 70.3597849 | 40.00434571 | 30.3554392 | 175 (175) |
| Djibouti | 0.487958371 | 19489.08795 | 15836.98969 | 3652.098258 | 197 (198) | 7203.741438 | 6306.528112 | 897.2133257 | 8 (7) | 49.19510636 | 40.00357128 | 9.191535085 | 38 (37) |
| Dominica | 0.746967185 | 23714.53216 | 15838.61843 | 7875.913727 | 132 (133) | 8522.134733 | 6306.792398 | 2215.342335 | 71 (70) | 51.138073 | 39.99741834 | 11.14065466 | 59 (59) |
| Dominican Republic | 0.619388201 | 23723.70841 | 15838.85722 | 7884.851184 | 127 (126) | 8525.749966 | 6306.065468 | 2219.684498 | 80 (78) | 51.14974509 | 40.00046056 | 11.14928453 | 60 (60) |
| Ecuador | 0.661017053 | 18708.71615 | 15839.46433 | 2869.251821 | 199 (200) | 7181.50051 | 6306.574203 | 874.9263073 | 6 (5) | 45.90323567 | 40.00344385 | 5.899791813 | 6 (5) |
| Egypt | 0.606787094 | 25845.29764 | 15838.95017 | 10006.34747 | 70 (70) | 8804.59632 | 6306.84401 | 2497.75231 | 126 (126) | 68.90405181 | 40.00021827 | 28.90383354 | 161 (161) |
| El Salvador | 0.563775188 | 23763.66103 | 15838.62407 | 7925.036961 | 108 (109) | 8536.063357 | 6306.372139 | 2229.691219 | 97 (96) | 51.85932128 | 39.99877429 | 11.86054699 | 80 (79) |
| Equatorial Guinea | 0.657857456 | 23202.58164 | 15838.52943 | 7364.052209 | 162 (163) | 8339.003529 | 6306.702202 | 2032.301326 | 43 (42) | 54.14860735 | 40.00195126 | 14.14665609 | 91 (90) |
| Eritrea | 0.403863943 | 19509.71414 | 15836.52257 | 3673.191575 | 188 (188) | 7208.670374 | 6306.705353 | 901.9650209 | 17 (17) | 49.60939323 | 39.99969203 | 9.609701196 | 45 (45) |
| Estonia | 0.844917787 | 30335.96941 | 15839.04158 | 14496.92784 | 34 (34) | 10612.75604 | 6306.576159 | 4306.179882 | 161 (161) | 87.75240904 | 40.000536 | 47.75187304 | 199 (199) |
| Eswatini | 0.585459713 | 23237.604 | 15837.49833 | 7400.105669 | 157 (158) | 8348.356389 | 6306.387773 | 2041.968616 | 47 (46) | 54.0355349 | 40.00035228 | 14.03518262 | 89 (88) |
| Ethiopia | 0.358823295 | 15855.3296 | 15840.75346 | 14.57613349 | 203 (204) | 6313.304922 | 6306.294871 | 7.010050739 | 2 (1) | 40.27339142 | 39.99779527 | 0.275596157 | 2 (1) |
| Fiji | 0.675051631 | 22173.80912 | 15837.59499 | 6336.214133 | 171 (171) | 8006.376429 | 6306.490174 | 1699.886255 | 33 (33) | 46.41029241 | 39.99759081 | 6.412701604 | 14 (13) |
| Finland | 0.859831368 | 32383.19655 | 15839.05221 | 16544.14434 | 19 (19) | 10996.08048 | 6306.35218 | 4689.728298 | 183 (183) | 69.26072351 | 40.00083889 | 29.25988462 | 164 (164) |
| France | 0.838364875 | 31418.47948 | 15838.05955 | 15580.41992 | 29 (29) | 10893.19806 | 6306.325994 | 4586.87207 | 176 (176) | 74.92087616 | 40.00021702 | 34.92065913 | 195 (195) |
| Gabon | 0.634691393 | 23246.37569 | 15838.13759 | 7408.238097 | 153 (153) | 8350.944358 | 6306.359872 | 2044.584486 | 52 (51) | 54.43572061 | 39.99950731 | 14.4362133 | 96 (95) |
| Gambia | 0.40971416 | 24545.0774 | 15837.3548 | 8707.722599 | 93 (95) | 8692.361269 | 6306.640487 | 2385.720782 | 116 (116) | 55.38538924 | 40.00977768 | 15.37561155 | 103 (102) |
| Georgia | 0.732473604 | 30274.87533 | 15837.96143 | 14436.9139 | 39 (40) | 10666.34321 | 6306.451668 | 4359.891547 | 169 (169) | 66.84364109 | 40.00103872 | 26.84260237 | 152 (152) |
| Germany | 0.902957091 | 33107.72532 | 15837.1196 | 17270.60572 | 8 (8) | 11012.92489 | 6306.638526 | 4706.286367 | 192 (192) | 72.20522955 | 39.99986334 | 32.20536621 | 179 (179) |
| Ghana | 0.56493039 | 24555.79629 | 15837.42357 | 8718.37272 | 88 (88) | 8693.425833 | 6306.811669 | 2386.614164 | 119 (120) | 55.94732995 | 39.99651196 | 15.95081798 | 114 (114) |
| Greece | 0.791854408 | 32408.70414 | 15837.00207 | 16571.70208 | 15 (15) | 11001.94935 | 6306.941285 | 4695.008066 | 189 (189) | 69.65274998 | 39.99850745 | 29.65424254 | 168 (168) |
| Greenland | 0.826210336 | 32837.19243 | 15837.87987 | 16999.31256 | 10 (10) | 11136.97564 | 6306.428925 | 4830.546711 | 195 (195) | 66.44476014 | 40.0028587 | 26.44190144 | 143 (143) |
| Grenada | 0.668993028 | 23710.78173 | 15838.00733 | 7872.774392 | 133 (134) | 8521.255956 | 6306.514088 | 2214.741867 | 69 (68) | 50.95620958 | 39.99971718 | 10.9564924 | 56 (56) |
| Guam | 0.803982203 | 22159.46105 | 15837.98037 | 6321.480682 | 175 (175) | 8001.531184 | 6306.583102 | 1694.948082 | 29 (29) | 46.94125922 | 40.00076586 | 6.940493361 | 25 (24) |
| Guatemala | 0.539972424 | 23746.00989 | 15836.91589 | 7909.094003 | 111 (112) | 8531.577884 | 6306.534574 | 2225.04331 | 94 (93) | 51.16535771 | 40.00257912 | 11.16277859 | 62 (62) |
| Guinea | 0.336401293 | 24553.7094 | 15837.43518 | 8716.274229 | 89 (90) | 8693.009939 | 6306.160847 | 2386.849092 | 120 (118) | 55.55248189 | 39.99974384 | 15.55273805 | 107 (107) |
| Guinea-Bissau | 0.353109621 | 24557.15298 | 15838.24792 | 8718.905061 | 87 (87) | 8694.212258 | 6306.455331 | 2387.756927 | 121 (121) | 55.55881691 | 40.00058415 | 15.55823276 | 108 (108) |
| Guyana | 0.650812335 | 23737.03171 | 15837.319 | 7899.712709 | 118 (119) | 8528.975642 | 6306.173394 | 2222.802248 | 87 (86) | 50.80818665 | 39.9995668 | 10.80861985 | 54 (54) |
| Haiti | 0.448278285 | 23736.28955 | 15839.06143 | 7897.228118 | 121 (120) | 8528.881492 | 6306.203206 | 2222.678286 | 86 (85) | 50.76345432 | 40.00371732 | 10.75973701 | 53 (53) |
| Honduras | 0.513037248 | 23745.73498 | 15838.96231 | 7906.772678 | 112 (113) | 8530.794452 | 6306.373194 | 2224.421259 | 92 (91) | 51.25934148 | 40.00040457 | 11.25893691 | 63 (64) |
| Hungary | 0.790754768 | 29976.1579 | 15838.53341 | 14137.62448 | 45 (45) | 10579.89127 | 6306.375951 | 4273.515314 | 158 (158) | 73.0745113 | 40.0036866 | 33.0708247 | 188 (188) |
| Iceland | 0.87636168 | 32368.66517 | 15838.74574 | 16529.91943 | 21 (21) | 10991.29033 | 6306.57723 | 4684.713102 | 181 (181) | 69.37158075 | 40.00153484 | 29.37004591 | 165 (165) |
| India | 0.575401649 | 25298.03881 | 15837.03395 | 9461.004863 | 74 (74) | 9333.372626 | 6306.60458 | 3026.768046 | 133 (133) | 51.87861851 | 40.0028647 | 11.87575381 | 82 (81) |
| Indonesia | 0.656868336 | 25774.92965 | 15837.68383 | 9937.245827 | 72 (72) | 9337.801093 | 6306.360724 | 3031.440369 | 134 (134) | 52.56354181 | 39.99975239 | 12.56378942 | 84 (83) |
| Iran (Islamic Republic of) | 0.697207398 | 27244.00066 | 15836.87356 | 11407.1271 | 63 (63) | 9497.895528 | 6306.862606 | 3191.032922 | 140 (140) | 74.73656099 | 40.00224724 | 34.73431375 | 194 (194) |
| Iraq | 0.662626231 | 23545.33709 | 15837.36696 | 7707.970124 | 139 (140) | 8509.356756 | 6306.849113 | 2202.507643 | 64 (63) | 65.89094987 | 40.0057239 | 25.88522597 | 135 (136) |
| Ireland | 0.87375385 | 32406.0159 | 15838.03958 | 16567.97632 | 16 (16) | 11000.32368 | 6306.48796 | 4693.835724 | 187 (186) | 69.57996299 | 40.00342094 | 29.57654205 | 167 (167) |
| Israel | 0.809011652 | 32400.53996 | 15837.812 | 16562.72796 | 17 (17) | 11000.05614 | 6306.445197 | 4693.610943 | 186 (185) | 69.79939189 | 40.00463689 | 29.794755 | 169 (169) |
| Italy | 0.805773534 | 33572.3107 | 15836.31394 | 17735.99676 | 6 (6) | 11907.29912 | 6306.483 | 5600.81612 | 201 (201) | 71.46291747 | 39.99945249 | 31.46346497 | 178 (178) |
| Jamaica | 0.683263064 | 23722.86309 | 15837.3756 | 7885.487493 | 125 (128) | 8524.371463 | 6306.245263 | 2218.1262 | 76 (75) | 51.53244959 | 40.00076204 | 11.53168755 | 72 (72) |
| Japan | 0.871241813 | 29705.70179 | 15837.4959 | 13868.20589 | 58 (58) | 10726.09348 | 6306.345083 | 4419.748397 | 174 (174) | 64.56326032 | 40.00165133 | 24.561609 | 132 (132) |
| Jordan | 0.725307227 | 23532.88494 | 15839.31352 | 7693.571415 | 144 (145) | 8508.465816 | 6306.679129 | 2201.786687 | 61 (60) | 65.89025899 | 40.00002586 | 25.89023313 | 136 (135) |
| Kazakhstan | 0.725144495 | 30289.04112 | 15838.09886 | 14450.94226 | 36 (36) | 10669.89002 | 6306.571991 | 4363.318027 | 172 (172) | 67.00482221 | 39.99936171 | 27.0054605 | 153 (153) |
| Kenya | 0.523768077 | 20758.45189 | 15836.23304 | 4922.218847 | 186 (186) | 7660.12867 | 6306.394825 | 1353.733846 | 20 (20) | 53.6035297 | 40.00102063 | 13.60250908 | 85 (84) |
| Kiribati | 0.527186583 | 22252.76869 | 15838.36346 | 6414.40523 | 165 (165) | 8031.738135 | 6306.386491 | 1725.351645 | 39 (39) | 46.49905716 | 39.99765276 | 6.5014044 | 19 (17) |
| Kuwait | 0.846651055 | 23548.03542 | 15837.73789 | 7710.297534 | 138 (139) | 8511.294805 | 6306.822185 | 2204.47262 | 65 (64) | 63.15982121 | 39.99866739 | 23.16115381 | 126 (126) |
| Kyrgyzstan | 0.603979328 | 30280.70587 | 15837.83042 | 14442.87546 | 38 (38) | 10667.6509 | 6306.50999 | 4361.140914 | 170 (170) | 67.08160299 | 39.99946721 | 27.08213578 | 155 (155) |
| Lao People's Democratic Republic | 0.489136091 | 24566.31247 | 15837.04278 | 8729.269681 | 85 (85) | 8681.168794 | 6306.098244 | 2375.07055 | 103 (101) | 48.95875684 | 40.00246173 | 8.956295112 | 34 (33) |
| Latvia | 0.830663516 | 30338.15207 | 15837.93509 | 14500.21698 | 33 (33) | 10613.16546 | 6306.381302 | 4306.784155 | 163 (162) | 87.85979139 | 39.9991096 | 47.86068178 | 200 (200) |
| Lebanon | 0.744746351 | 23534.28422 | 15837.51549 | 7696.768732 | 142 (143) | 8508.61256 | 6306.549963 | 2202.062597 | 62 (61) | 66.54825291 | 40.00530048 | 26.54295243 | 144 (144) |
| Lesotho | 0.510393066 | 23245.90362 | 15837.38656 | 7408.517061 | 152 (154) | 8351.095759 | 6306.215432 | 2044.880327 | 53 (52) | 53.72735696 | 39.99753908 | 13.72981787 | 86 (85) |
| Liberia | 0.352442452 | 24518.93141 | 15836.43621 | 8682.495196 | 103 (105) | 8685.736865 | 6306.306644 | 2379.430221 | 106 (105) | 54.33161085 | 40.00340898 | 14.32820187 | 93 (92) |
| Libya | 0.725771399 | 23542.44872 | 15837.21565 | 7705.233072 | 140 (141) | 8509.205514 | 6306.823266 | 2202.382249 | 63 (62) | 66.21977837 | 40.00474714 | 26.21503124 | 138 (138) |
| Lithuania | 0.856484049 | 31729.84489 | 15836.07408 | 15893.7708 | 26 (26) | 10764.37457 | 6306.115755 | 4458.258814 | 175 (175) | 81.03148255 | 39.99702942 | 41.03445313 | 198 (198) |
| Luxembourg | 0.884428955 | 32279.9639 | 15837.46552 | 16442.49837 | 23 (23) | 10969.41916 | 6306.396557 | 4663.022599 | 179 (179) | 74.97635596 | 39.99887311 | 34.97748285 | 196 (196) |
| Madagascar | 0.400246943 | 19501.66315 | 15838.37955 | 3663.283595 | 194 (195) | 7206.090306 | 6306.514501 | 899.575805 | 12 (11) | 49.89703725 | 40.00021717 | 9.896820078 | 50 (50) |
| Malawi | 0.384553634 | 19504.05047 | 15837.54177 | 3666.508701 | 190 (193) | 7206.438268 | 6306.371191 | 900.0670765 | 13 (12) | 49.79145964 | 40.00048337 | 9.790976265 | 46 (46) |
| Malaysia | 0.742523828 | 24519.05652 | 15837.16816 | 8681.888368 | 104 (104) | 8667.010246 | 6306.410532 | 2360.599713 | 100 (99) | 48.99083758 | 40.00479119 | 8.986046398 | 35 (34) |
| Maldives | 0.650886627 | 24278.21184 | 15838.53982 | 8439.672025 | 107 (108) | 8591.775375 | 6306.601405 | 2285.173971 | 98 (97) | 48.1640515 | 40.0005211 | 8.163530404 | 27 (26) |
| Mali | 0.268579941 | 24533.93754 | 15837.26792 | 8696.669613 | 97 (98) | 8688.864903 | 6306.360803 | 2382.5041 | 109 (107) | 55.08984079 | 40.00308195 | 15.08675884 | 100 (99) |
| Malta | 0.801585034 | 32376.70619 | 15838.01497 | 16538.69122 | 20 (20) | 10993.93753 | 6306.355851 | 4687.581679 | 182 (182) | 69.24511395 | 40.00150681 | 29.24360713 | 163 (163) |
| Marshall Islands | 0.574091128 | 22147.43179 | 15837.25848 | 6310.173309 | 178 (177) | 7998.83211 | 6306.474195 | 1692.357916 | 27 (27) | 46.14839124 | 40.0039855 | 6.144405741 | 10 (9) |
| Mauritania | 0.4989451 | 24545.60973 | 15840.56739 | 8705.042344 | 94 (93) | 8691.915958 | 6306.7448 | 2385.171158 | 114 (114) | 55.94142198 | 40.00482337 | 15.93659861 | 113 (113) |
| Mauritius | 0.718260446 | 24573.74331 | 15837.59494 | 8736.148369 | 84 (84) | 8683.300843 | 6306.235276 | 2377.065567 | 104 (103) | 48.84384673 | 40.00141634 | 8.842430389 | 30 (29) |
| Mexico | 0.664575304 | 25068.14567 | 15839.73705 | 9228.408626 | 75 (75) | 9118.526498 | 6306.791772 | 2811.734726 | 131 (131) | 55.2145898 | 40.00113526 | 15.21345454 | 101 (100) |
| Micronesia (Federated States of) | 0.587534967 | 22170.51125 | 15837.70032 | 6332.810927 | 172 (172) | 8004.741183 | 6306.550462 | 1698.190721 | 31 (31) | 46.4685162 | 40.0010443 | 6.467471894 | 17 (16) |
| Monaco | 0.908262831 | 32411.21203 | 15837.67854 | 16573.53349 | 14 (14) | 11000.464 | 6306.863195 | 4693.600806 | 185 (187) | 69.9386071 | 40.00037128 | 29.93823582 | 172 (172) |
| Mongolia | 0.617621565 | 30281.80571 | 15836.86762 | 14444.93809 | 37 (37) | 10668.43428 | 6306.597457 | 4361.836819 | 171 (171) | 66.78229105 | 40.00124301 | 26.78104804 | 149 (149) |
| Montenegro | 0.795800584 | 29955.13032 | 15838.05244 | 14117.07788 | 50 (49) | 10573.96097 | 6306.359872 | 4267.601099 | 153 (153) | 73.04381987 | 40.00058753 | 33.04323234 | 187 (187) |
| Morocco | 0.562698301 | 23523.54228 | 15837.50776 | 7686.034525 | 148 (149) | 8502.988115 | 6306.282671 | 2196.705444 | 58 (57) | 66.29327528 | 40.00861521 | 26.28466008 | 139 (139) |
| Mozambique | 0.326462614 | 19504.07069 | 15838.88734 | 3665.18335 | 192 (192) | 7205.814432 | 6306.845537 | 898.9688948 | 11 (10) | 49.23184677 | 39.99902037 | 9.232826408 | 41 (40) |
| Myanmar | 0.53390084 | 24636.81513 | 15838.60827 | 8798.206858 | 78 (78) | 8702.372836 | 6306.472989 | 2395.899847 | 125 (125) | 49.08439565 | 40.00196873 | 9.082426919 | 36 (35) |
| Namibia | 0.617564872 | 23242.32308 | 15838.03217 | 7404.290916 | 156 (157) | 8350.688729 | 6306.464737 | 2044.223992 | 50 (49) | 54.52713805 | 40.00088751 | 14.52625054 | 97 (96) |
| Nauru | 0.625177834 | 22210.9778 | 15838.60585 | 6372.371951 | 168 (168) | 8017.975529 | 6306.380002 | 1711.595528 | 36 (36) | 46.49947396 | 39.99875049 | 6.500723473 | 18 (18) |
| Nepal | 0.433174635 | 24613.26782 | 15837.17461 | 8776.093206 | 81 (81) | 8883.193866 | 6306.690016 | 2576.50385 | 127 (127) | 50.68794394 | 39.99771006 | 10.69023388 | 52 (52) |
| Netherlands | 0.888464256 | 34984.91391 | 15836.09415 | 19148.81976 | 2 (2) | 11114.03531 | 6306.488482 | 4807.546828 | 194 (194) | 68.15914234 | 39.9993335 | 28.15980884 | 159 (159) |
| New Zealand | 0.849442499 | 28452.74216 | 15837.75737 | 12614.98478 | 62 (62) | 10345.43006 | 6306.345859 | 4039.084205 | 146 (146) | 64.04770967 | 39.99807148 | 24.04963818 | 130 (130) |
| Nicaragua | 0.523958472 | 23736.00754 | 15836.8888 | 7899.118744 | 119 (121) | 8528.864672 | 6306.319787 | 2222.544885 | 85 (84) | 51.30276993 | 40.00290892 | 11.29986101 | 67 (67) |
| Niger | 0.168072774 | 24545.47104 | 17417.49777 | 7127.973269 | 163 (94) | 8691.828692 | 6786.157196 | 1905.671496 | 41 (113) | 55.42745217 | 44.01120025 | 11.41625192 | 69 (104) |
| Nigeria | 0.503390833 | 25749.89044 | 15836.35278 | 9913.537663 | 73 (73) | 9322.133576 | 6306.658804 | 3015.474772 | 132 (132) | 59.80976259 | 40.00021977 | 19.80954282 | 119 (119) |
| Niue | 0.72622205 | 22182.0011 | 15837.10789 | 6344.893207 | 169 (169) | 8008.376811 | 6306.491448 | 1701.885363 | 34 (34) | 46.66289743 | 40.0000954 | 6.662802031 | 21 (20) |
| North Macedonia | 0.750629703 | 29923.18935 | 15837.67253 | 14085.51681 | 56 (56) | 10567.29988 | 6306.488919 | 4260.810962 | 149 (149) | 72.65112306 | 40.00039367 | 32.65072939 | 181 (181) |
| Northern Mariana Islands | 0.771535213 | 22111.12432 | 15838.30328 | 6272.821042 | 181 (181) | 7985.96352 | 6306.316112 | 1679.647408 | 23 (23) | 46.70164859 | 39.99879281 | 6.702855785 | 22 (21) |
| Norway | 0.91613281 | 35492.43834 | 15838.23063 | 19654.20771 | 1 (1) | 12225.67197 | 6306.770464 | 5918.901502 | 204 (204) | 71.25076805 | 39.99800245 | 31.2527656 | 177 (177) |
| Oman | 0.773391602 | 23580.67766 | 15837.15628 | 7743.521384 | 135 (136) | 8531.380302 | 6306.499718 | 2224.880584 | 93 (92) | 64.81302681 | 39.99843364 | 24.81459317 | 133 (133) |
| Pakistan | 0.504028689 | 26707.02729 | 15837.75393 | 10869.27336 | 65 (65) | 9677.339631 | 6306.324746 | 3371.014885 | 141 (141) | 46.10168545 | 40.00170801 | 6.099977445 | 8 (7) |
| Palau | 0.754046931 | 22029.78497 | 15838.73833 | 6191.04664 | 182 (182) | 7958.89668 | 6306.2382 | 1652.65848 | 22 (22) | 46.09209643 | 40.00382603 | 6.088270392 | 7 (6) |
| Palestine | 0.631011665 | 23528.29183 | 15837.81891 | 7690.472919 | 145 (146) | 8503.563076 | 6306.510101 | 2197.052975 | 59 (58) | 66.29651034 | 40.00334138 | 26.29316896 | 140 (140) |
| Panama | 0.708864828 | 23715.9857 | 15836.60872 | 7879.376983 | 130 (132) | 8522.630046 | 6306.600089 | 2216.029957 | 72 (71) | 51.32075703 | 39.99984128 | 11.32091575 | 68 (68) |
| Papua New Guinea | 0.417797443 | 22129.08422 | 15838.41968 | 6290.664549 | 180 (180) | 7993.1977 | 6306.925301 | 1686.272399 | 24 (24) | 46.12172061 | 39.99907961 | 6.122640993 | 9 (8) |
| Paraguay | 0.635718099 | 26396.49447 | 15839.45004 | 10557.04444 | 66 (66) | 9347.447889 | 6306.666416 | 3040.781473 | 136 (136) | 51.27326687 | 40.00408046 | 11.26918641 | 65 (65) |
| Peru | 0.662054037 | 21862.34015 | 15838.49266 | 6023.847496 | 183 (183) | 7756.035505 | 6306.45766 | 1449.577845 | 21 (21) | 49.42707742 | 39.99952411 | 9.427553308 | 44 (44) |
| Philippines | 0.651219329 | 25783.42505 | 15837.83934 | 9945.585709 | 71 (71) | 9339.716298 | 6306.235732 | 3033.480566 | 135 (135) | 52.38215885 | 39.99787275 | 12.38428609 | 83 (82) |
| Poland | 0.812042809 | 31533.24489 | 15837.15492 | 15696.08998 | 28 (28) | 11365.28527 | 6306.835783 | 5058.449483 | 198 (198) | 78.06087652 | 40.00518305 | 38.05569347 | 197 (197) |
| Portugal | 0.744151851 | 32429.22553 | 15837.69043 | 16591.5351 | 12 (12) | 11006.84077 | 6306.208605 | 4700.632167 | 191 (191) | 69.80037794 | 40.00065431 | 29.79972362 | 170 (170) |
| Puerto Rico | 0.825525847 | 23740.88226 | 15838.9505 | 7901.931764 | 115 (116) | 8529.727062 | 6306.324822 | 2223.40224 | 89 (87) | 51.78265639 | 39.99827254 | 11.78438385 | 79 (78) |
| Qatar | 0.846860584 | 23526.77242 | 15838.17021 | 7688.602217 | 147 (147) | 8512.825457 | 6306.472143 | 2206.353314 | 66 (65) | 63.19524469 | 39.99907682 | 23.19616787 | 127 (127) |
| Republic of Korea | 0.886675267 | 30412.34572 | 15838.50527 | 14573.84045 | 31 (31) | 10316.73562 | 6306.610433 | 4010.12519 | 145 (145) | 62.3898416 | 40.00295443 | 22.38688717 | 125 (125) |
| Republic of Moldova | 0.732214875 | 30334.65068 | 15837.78539 | 14496.86528 | 35 (35) | 10611.65676 | 6306.536736 | 4305.120025 | 160 (160) | 87.90130344 | 40.0002192 | 47.90108424 | 201 (201) |
| Romania | 0.768453864 | 29957.4698 | 15837.24008 | 14120.22972 | 47 (48) | 10575.83485 | 6306.346073 | 4269.488782 | 156 (156) | 73.08053356 | 40.00028304 | 33.08025052 | 190 (190) |
| Russian Federation | 0.808536005 | 31147.26967 | 15837.90201 | 15309.36766 | 30 (30) | 11200.6375 | 6306.488412 | 4894.149092 | 197 (197) | 99.72339315 | 40.00099933 | 59.72239382 | 204 (204) |
| Rwanda | 0.435588706 | 19506.58729 | 15838.69357 | 3667.893718 | 189 (190) | 7206.917874 | 6306.538862 | 900.3790124 | 14 (13) | 50.11525724 | 40.00035228 | 10.11490496 | 51 (51) |
| Saint Kitts and Nevis | 0.754987055 | 23738.22721 | 15838.45235 | 7899.774859 | 117 (118) | 8530.007063 | 6306.712115 | 2223.294948 | 88 (88) | 51.2911184 | 40.00272396 | 11.28839444 | 66 (66) |
| Saint Lucia | 0.672509735 | 23718.12403 | 15836.22251 | 7881.901519 | 129 (130) | 8523.505445 | 6306.751678 | 2216.753767 | 73 (73) | 51.11466619 | 40.00203481 | 11.11263137 | 58 (58) |
| Saint Vincent and the Grenadines | 0.637195963 | 23704.30242 | 15839.17459 | 7865.127823 | 134 (135) | 8518.920769 | 6306.259087 | 2212.661682 | 67 (66) | 50.94322917 | 40.00178543 | 10.94144374 | 55 (55) |
| Samoa | 0.593392769 | 22147.1512 | 15835.96897 | 6311.182229 | 177 (178) | 7998.215501 | 6307.203611 | 1691.01189 | 26 (26) | 46.4671267 | 40.00238247 | 6.464744229 | 16 (15) |
| San Marino | 0.888005474 | 32430.83813 | 15837.21298 | 16593.62515 | 11 (11) | 11005.35776 | 6306.28732 | 4699.070437 | 190 (190) | 70.07442167 | 40.00329629 | 30.07112538 | 173 (173) |
| Sao Tome and Principe | 0.505413747 | 24532.89183 | 15836.88683 | 8696.004999 | 98 (99) | 8689.768521 | 6306.838287 | 2382.930235 | 112 (111) | 55.72625899 | 39.99713794 | 15.72912105 | 110 (110) |
| Saudi Arabia | 0.815143493 | 22434.33042 | 15837.27517 | 6597.055251 | 164 (164) | 8335.678199 | 6307.531249 | 2028.14695 | 42 (41) | 63.97565495 | 40.00057183 | 23.97508312 | 129 (129) |
| Senegal | 0.408054193 | 24528.5547 | 15837.07487 | 8691.479829 | 101 (103) | 8687.013487 | 6306.397913 | 2380.615574 | 108 (106) | 55.38705633 | 39.99600168 | 15.39105466 | 104 (103) |
| Serbia | 0.792416294 | 29928.17205 | 15838.39789 | 14089.77416 | 55 (54) | 10566.40713 | 6306.247613 | 4260.15952 | 148 (148) | 73.08219324 | 39.99891492 | 33.08327833 | 191 (191) |
| Seychelles | 0.730150775 | 24479.93002 | 15837.76666 | 8642.163356 | 106 (107) | 8653.336488 | 6306.577698 | 2346.75879 | 99 (98) | 48.90470734 | 40.00363942 | 8.901067916 | 32 (31) |
| Sierra Leone | 0.358665881 | 24535.32986 | 15838.01108 | 8697.318778 | 96 (97) | 8690.349174 | 6306.545273 | 2383.803901 | 113 (112) | 55.2186509 | 39.99701204 | 15.22163886 | 102 (101) |
| Singapore | 0.856097766 | 28481.36284 | 15837.17966 | 12644.18318 | 61 (61) | 10098.06653 | 6306.160847 | 3791.905687 | 143 (143) | 59.6775206 | 40.00071717 | 19.67680343 | 118 (118) |
| Slovakia | 0.81061053 | 29962.04577 | 15838.50475 | 14123.54103 | 46 (46) | 10576.73798 | 6306.650084 | 4270.087896 | 157 (157) | 73.13158984 | 40.00140176 | 33.13018808 | 192 (192) |
| Slovenia | 0.842430731 | 29927.39252 | 15837.34503 | 14090.04749 | 54 (55) | 10568.36216 | 6306.615025 | 4261.747136 | 151 (151) | 72.89139271 | 39.99980214 | 32.89159056 | 185 (185) |
| Solomon Islands | 0.429360316 | 22166.62308 | 15836.3893 | 6330.233779 | 173 (173) | 8004.91308 | 6306.394574 | 1698.518506 | 32 (32) | 46.3484581 | 40.00094278 | 6.347515323 | 12 (11) |
| Somalia | 0.077688109 | 19507.49741 | 19494.37047 | 13.12693865 | 204 (189) | 7207.645192 | 7203.036896 | 4.608296413 | 1 (15) | 49.28692751 | 49.04964105 | 0.237286459 | 1 (42) |
| South Africa | 0.679626598 | 24528.7092 | 15839.29497 | 8689.41423 | 102 (102) | 8928.226971 | 6306.98415 | 2621.242821 | 130 (130) | 57.93150549 | 40.00237558 | 17.92912991 | 117 (117) |
| South Sudan | 0.278371125 | 19484.85935 | 15838.8867 | 3645.97265 | 198 (199) | 7199.999656 | 6306.348156 | 893.6515003 | 7 (6) | 49.21457614 | 40.00214993 | 9.212426209 | 39 (38) |
| Spain | 0.769283698 | 31535.62581 | 15838.45648 | 15697.16933 | 27 (27) | 10896.82765 | 6306.373018 | 4590.454627 | 177 (177) | 73.68685774 | 40.00877136 | 33.67808638 | 193 (193) |
| Sri Lanka | 0.701534935 | 24617.15204 | 15838.81098 | 8778.34106 | 80 (80) | 8696.583077 | 6306.682007 | 2389.901069 | 123 (123) | 49.12198341 | 39.99942826 | 9.122555151 | 37 (36) |
| Sudan | 0.541949735 | 23470.17162 | 15836.81709 | 7633.354532 | 151 (152) | 8485.197609 | 6306.562188 | 2178.635422 | 54 (53) | 66.3883572 | 40.00159536 | 26.38676184 | 142 (142) |
| Suriname | 0.633665739 | 23735.03092 | 15838.37931 | 7896.651616 | 122 (123) | 8527.47527 | 6306.732711 | 2220.742559 | 81 (80) | 51.10656382 | 40.00413492 | 11.10242891 | 57 (57) |
| Sweden | 0.886880299 | 34297.27358 | 15837.83718 | 18459.4364 | 4 (4) | 11957.24394 | 6306.619933 | 5650.624007 | 202 (202) | 68.31134226 | 39.99636908 | 28.31497318 | 160 (160) |
| Switzerland | 0.933059111 | 29829.1988 | 15839.95901 | 13989.23979 | 57 (57) | 10713.80299 | 6306.659821 | 4407.143173 | 173 (173) | 66.36282415 | 40.00109354 | 26.36173061 | 141 (141) |
| Syrian Arab Republic | 0.623004075 | 23519.32591 | 15839.1852 | 7680.140705 | 149 (150) | 8488.869065 | 6306.708933 | 2182.160132 | 55 (54) | 67.11094924 | 40.00222489 | 27.10872434 | 156 (156) |
| Taiwan (Province of China) | 0.874747053 | 17557.80816 | 15837.56098 | 1720.24718 | 202 (203) | 6542.128082 | 6306.783553 | 235.3445289 | 3 (2) | 41.12945931 | 40.0017843 | 1.127675002 | 4 (3) |
| Tajikistan | 0.541511187 | 30255.25422 | 15837.39944 | 14417.85478 | 44 (44) | 10660.55522 | 6306.257567 | 4354.297656 | 164 (164) | 66.72721933 | 40.00297212 | 26.72424721 | 148 (148) |
| Thailand | 0.682547933 | 24604.28675 | 15837.88707 | 8766.399678 | 82 (82) | 8692.881773 | 6306.7191 | 2386.162673 | 117 (117) | 49.24632882 | 40.00106125 | 9.245267565 | 42 (41) |
| Timor-Leste | 0.444667619 | 24554.54287 | 15839.65161 | 8714.891263 | 90 (89) | 8679.382082 | 6306.585943 | 2372.796139 | 101 (100) | 48.68667588 | 40.00166172 | 8.685014162 | 29 (28) |
| Togo | 0.408533695 | 24549.88925 | 15838.0828 | 8711.806451 | 91 (92) | 8692.034513 | 6306.346073 | 2385.68844 | 115 (115) | 55.89534843 | 40.00161681 | 15.89373162 | 112 (112) |
| Tokelau | 0.686425621 | 22166.22673 | 15838.47629 | 6327.750437 | 174 (174) | 8003.896084 | 6306.481628 | 1697.414456 | 30 (30) | 46.52285933 | 39.99975067 | 6.523108661 | 20 (19) |
| Tonga | 0.626349936 | 22214.77941 | 15837.18759 | 6377.591822 | 167 (167) | 8020.273279 | 6306.259445 | 1714.013834 | 37 (37) | 46.80682549 | 40.00253242 | 6.804293075 | 23 (22) |
| Trinidad and Tobago | 0.768763254 | 23723.31153 | 15838.17817 | 7885.133357 | 126 (127) | 8525.208819 | 6306.48796 | 2218.720859 | 78 (77) | 51.15228192 | 40.00170801 | 11.15057391 | 61 (61) |
| Tunisia | 0.682432216 | 23526.34883 | 15837.30872 | 7689.040113 | 146 (148) | 8502.170521 | 6306.474503 | 2195.696018 | 57 (56) | 66.62520858 | 39.99936328 | 26.6258453 | 145 (145) |
| Turkmenistan | 0.682160776 | 30262.16356 | 15837.80605 | 14424.35751 | 43 (43) | 10664.40023 | 6306.710461 | 4357.689771 | 166 (166) | 66.70252847 | 40.00452603 | 26.69800245 | 147 (147) |
| Tuvalu | 0.576620529 | 22139.85743 | 15839.42409 | 6300.433338 | 179 (179) | 7994.475374 | 6306.601995 | 1687.873379 | 25 (25) | 46.4586078 | 40.00676273 | 6.45184507 | 15 (14) |
| Türkiye | 0.712692673 | 21659.03572 | 15836.98517 | 5822.050544 | 184 (184) | 8176.283976 | 6306.36224 | 1869.921736 | 40 (40) | 66.80017475 | 39.99940009 | 26.80077467 | 151 (151) |
| Uganda | 0.423261181 | 19499.97337 | 15839.71047 | 3660.262903 | 196 (196) | 7204.820167 | 6306.922999 | 897.8971677 | 9 (9) | 49.87040256 | 39.99961288 | 9.870789679 | 49 (49) |
| Ukraine | 0.760773913 | 31872.73639 | 15838.95208 | 16033.78432 | 24 (24) | 11405.06894 | 6306.567894 | 5098.501051 | 199 (199) | 91.72499176 | 40.00363882 | 51.72135294 | 203 (203) |
| United Arab Emirates | 0.849317734 | 23538.74516 | 15838.0892 | 7700.655969 | 141 (142) | 8525.009943 | 6306.406558 | 2218.603385 | 77 (76) | 63.46291796 | 40.00291107 | 23.46000689 | 128 (128) |
| United Kingdom | 0.859000182 | 33487.02013 | 15838.00831 | 17649.01182 | 7 (7) | 11812.17184 | 6306.479162 | 5505.692678 | 200 (200) | 72.27253605 | 39.99829981 | 32.27423625 | 180 (180) |
| United Republic of Tanzania | 0.446568273 | 19499.53993 | 15837.37015 | 3662.169777 | 195 (197) | 7204.816178 | 6306.424271 | 898.3919073 | 10 (8) | 49.84808973 | 39.99850705 | 9.849582679 | 48 (48) |
| United States Virgin Islands | 0.821830853 | 23738.91975 | 15837.43547 | 7901.484274 | 116 (117) | 8528.495223 | 6306.136285 | 2222.358938 | 84 (82) | 51.63355031 | 40.00313094 | 11.63041936 | 74 (73) |
| United States of America | 0.862448354 | 34320.58449 | 15837.57574 | 18483.00874 | 3 (3) | 12110.67566 | 6306.801975 | 5803.873687 | 203 (203) | 70.31533991 | 40.00309081 | 30.31224911 | 174 (174) |
| Uruguay | 0.719283445 | 26281.31928 | 15836.77866 | 10444.54062 | 67 (67) | 9488.894612 | 6306.50251 | 3182.392103 | 139 (139) | 60.01656975 | 40.00043318 | 20.01613656 | 122 (122) |
| Uzbekistan | 0.662621694 | 30269.38575 | 15836.7918 | 14432.59395 | 41 (41) | 10664.57221 | 6306.435022 | 4358.137185 | 167 (167) | 66.7886813 | 40.00010065 | 26.78858065 | 150 (150) |
| Vanuatu | 0.473100706 | 22181.19979 | 15836.81856 | 6344.381225 | 170 (170) | 8009.395766 | 6306.585524 | 1702.810242 | 35 (35) | 46.40721173 | 40.00042287 | 6.406788866 | 13 (12) |
| Venezuela (Bolivarian Republic of) | 0.596513059 | 23756.84675 | 15838.72342 | 7918.123334 | 109 (110) | 8534.605101 | 6306.805779 | 2227.799322 | 96 (95) | 51.5318469 | 39.99792324 | 11.53392366 | 73 (71) |
| Viet Nam | 0.627933721 | 24581.86587 | 15837.41402 | 8744.451846 | 83 (83) | 8684.614264 | 6306.203733 | 2378.410531 | 105 (104) | 49.37690481 | 39.99821 | 9.378694811 | 43 (43) |
| Yemen | 0.450376375 | 23532.89604 | 15836.99127 | 7695.90477 | 143 (144) | 8505.133039 | 6306.389484 | 2198.743555 | 60 (59) | 66.08003506 | 40.00679758 | 26.07323748 | 137 (137) |
| Zambia | 0.505948954 | 21241.52666 | 15836.65135 | 5404.875307 | 185 (185) | 7475.239304 | 6306.545195 | 1168.694109 | 18 (18) | 57.43787696 | 40.00501881 | 17.43285814 | 116 (116) |
| Zimbabwe | 0.473819486 | 23244.83211 | 15836.94031 | 7407.891801 | 154 (155) | 8350.660233 | 6306.587858 | 2044.072375 | 49 (48) | 54.40483395 | 40.00025907 | 14.40457488 | 95 (94) |
