# Supplementary material for: Global burden trends of tension-type headache, 1990–2021: socio-demographic patterns, age-period-cohort effects, and frontier analysis from the GBD 2021 study
Source: Front Neurol. 2025 Jul 16;16:1629025. doi: 10.3389/fneur.2025.1629025 (PMC12308845; doi:10.3389/fneur.2025.1629025)
Supplement: Supplementary file 2 [file Table_2.DOCX]

**Glossary of Key Terms**

**Age-Period-Cohort (APC) Model:** A statistical model used to disentangle three time-related effects on health trends: the effect of aging (Age), the influence of a specific time period (Period), and the unique experiences of a group of people born in the same era (Cohort).

**Local Drift:** The age-specific annual percentage change in incidence or prevalence rates. "Positive Drift" indicates that the rate for that age group is increasing over time.

**Net Drift:** The overall average annual percentage change across all age groups over the study period. A negative net drift indicates a general decreasing trend.

**Frontier Analysis:** An analytical method, adapted from economics, used to identify the best possible outcome theoretically achievable at a given level of a specific factor, in this case, the Socio-demographic Index (SDI).

**Improvement Potential**: In frontier analysis, this refers to the gap between a country's observed disease burden and the "frontier" (the minimum achievable burden at its SDI level). It quantifies the potential reduction in a country's disease burden if it were to match the performance of the best-performing countries at a similar stage of development.

**Uncertainty Interval (UI)**: A Bayesian credible interval representing the 2.5th and 97.5th percentiles of the posterior distribution of an estimate. According to the Institute for Health Metrics and Evaluation (IHME), a UI is "a range of values that reflects the certainty of an estimate. In GBD, every estimate is calculated 1,000 times, each time sampling from distributions rather than point estimates for data inputs, data transformations and model choice." In essence, it reflects the total uncertainty from all sources in the GBD's complex modeling process (including measurement error, systematic biases, and modeling choices).

**Confidence Interval (CI):** A frequentist interval designed to quantify the uncertainty attributable to sampling error for a sample statistic or model parameter. In principle, if the analysis were repeated on numerous samples, 95% of the calculated intervals would contain the true parameter value. It reflects the sampling variability inherent in the statistical estimates generated for this study.
